# Through the Looking Glass: A Systematic Review of Longitudinal Evidence, Providing New Insight for Motor Competence and Health

**DOI:** 10.1007/s40279-021-01516-8

**Published:** 2021-08-31

**Authors:** Lisa M. Barnett, E. Kipling Webster, Ryan M. Hulteen, An De Meester, Nadia C. Valentini, Matthieu Lenoir, Caterina Pesce, Nancy Getchell, Vitor P. Lopes, Leah E. Robinson, Ali Brian, Luis P. Rodrigues

**Affiliations:** 1 Institute for Physical Activity and Nutrition, School of Health and Social Development, Deakin University, Melbourne, VIC Australia; 2 Institute of Public and Preventive Health, Augusta University, Augusta, GA USA; 3 School of Kinesiology, Louisiana State University, Baton Rouge, LA USA; 4 Department of Physical Education, University of South Carolina, Columbia, SC USA; 5 Department of Physical Education, Physiotherapy, and Dance, Universidade Federal Do Rio Grande Do Sul, Porto Alegre, Brazil; 6 Department of Movement and Sports Sciences, Ghent University, Gent, Belgium; 7 Department of Movement, University of Rome “Foro Italico”, Human and Health Sciences, Rome, Italy; 8 Department of Kinesiology and Applied Physiology, University of Delaware, Newark, DE USA; 9 Research Center in Sports Sciences, Health Sciences and Human Development (CIDESD), Vila Real, Portugal; 10 Instituto Politécnico de Bragança, Campus de Santa Apolónia, 5300-223 Bragança, Portugal; 11 School of Kinesiology, University of Michigan, Ann Arbor, MI USA; 12 Instituto Politécnico de Viana do Castelo, Escola Superior Desporto e Lazer de Melgaço, Viana do Castelo, Portugal

## Abstract

**Introduction:**

In 2008, a conceptual model explaining the role of motor competence (MC) in children’s physical activity (PA), weight status, perceived MC and health-related fitness was published.

**Objective:**

The purpose of the current review was to systematically compile mediation, longitudinal and experimental evidence in support of this conceptual model.

**Methods:**

This systematic review (registered with PROSPERO on 28 April 2020) was conducted in accordance with the Preferred Reporting Items for Systematic Review and Meta-Analysis (PRISMA) statement. Separate searches were undertaken for each pathway of interest (final search 8 November 2019) using CINAHL Complete, ERIC, Medline (OVID), PsycINFO, Web of Science Core Collection, Scopus and SportDiscus. Potential articles were initially identified through abstract and title checking (*N* = 585) then screened further and combined into one review (*n* = 152), with 43 articles identified for extraction. Studies needed to be original and peer reviewed, include typically developing children and adolescents first assessed between 2 and 18 years and objective assessment of gross MC and at least one other variable (i.e., PA, weight status, perceived MC, health-related fitness). PA included sport participation, but sport-specific samples were excluded. Longitudinal or experimental designs and cross-sectional mediated models were sought. Strength of evidence was calculated for each pathway in both directions for each domain (i.e., skill composite, object control and locomotor/coordination/stability) by dividing the proportion of studies indicating a significantly positive pathway in the hypothesised direction by the total associations examined for that pathway. Classifications were no association (0–33%), indeterminate/inconsistent (34–59%), or a positive ‘+’ or negative ‘ − ’ association (≥ 60%). The latter category was classified as strong evidence (i.e., ++or −−) when four or more studies found an association. If the total number of studies in a domain of interest was three or fewer, this was considered insufficient evidence to make a determination.

**Results:**

There was strong evidence in both directions for a negative association between MC and weight status. There was strong positive evidence for a pathway from MC to fitness and indeterminate evidence for the reverse. There was indeterminate evidence for a pathway from MC to PA and no evidence for the reverse pathway. There was insufficient evidence for the MC to perceived MC pathway. There was strong positive evidence for the fitness-mediated MC/PA pathway in both directions. There was indeterminate evidence for the perceived MC-mediated pathway from PA to MC and no evidence for the reverse.

**Conclusion:**

Bidirectional longitudinal associations of MC with weight status are consistent with the model authored by Stodden et al. (Quest 2008;60(2):290–306, 2008). However, to test the whole model, the field needs robust longitudinal studies across childhood and adolescence that include all variables in the model, have multiple time points and account for potential confounding factors. Furthermore, experimental studies that examine change in MC relative to change in the other constructs are needed.

**Trial Registrations:**

PROSPERO ID# CRD42020155799.

**Supplementary Information:**

The online version contains supplementary material available at 10.1007/s40279-021-01516-8.

## Key Points

**Table:** 

In terms of pathways, our study found strong evidence for a negative association between weight status and motor competence (MC). There was strong positive evidence for a pathway from MC to fitness and indeterminate evidence for the reverse. There was indeterminate evidence for a MC to physical activity (PA) pathway and no evidence for the reverse. There was insufficient evidence between MC and perceived MC.	
Conclusions on mediation outcomes are weakened by the predominantly cross-sectional nature of the available evidence and the limited studies, although there was strong positive evidence for the fitness-mediating MC/PA pathway in both directions. There was indeterminate evidence for the perceived MC-mediated pathway from PA to MC and no evidence for the reverse.	
The field needs more robust longitudinal and experimental studies to test the Stodden et al. model.	

## Introduction

**Fig. 1:**
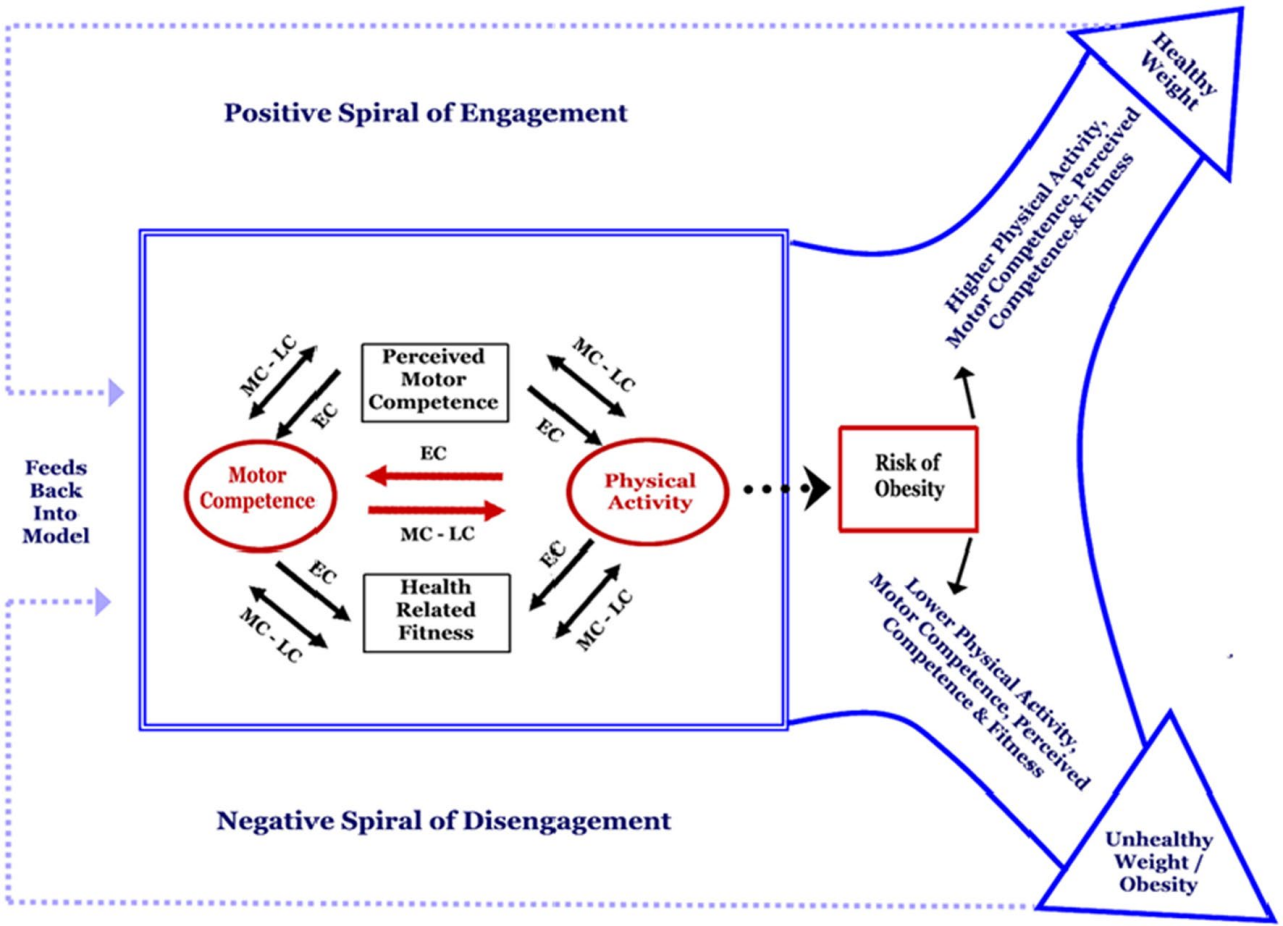
Conceptual model of motor development posed by Stodden et al. [2]. *EC* early childhood, *LC* late childhood, *MC* middle childhood. Reproduced from Stodden et al. [2] with permission

Motor development research has recently increased its focus on public health [1], largely triggered by the conceptual model developed by Stodden et al. [2], designed to explain the role of motor competence (MC) in multiple health-related aspects of child development, including children’s physical activity (PA) (any bodily movement resulting in energy expenditure [3]), weight status, perceived MC (perception of one’s own MC [4]) and health-related fitness (functional to health, e.g., endurance, flexibility [3]). Figure 1 shows the original model with a direct pathway proposed between MC and PA and mediating pathways via perceived MC and health-related physical fitness. The directions of these pathways were proposed to change as a function of developmental time. That is, PA drives MC in early childhood; however, in middle and late childhood, MC drives PA. The interaction among all these variables was also proposed to inversely relate to bodyweight status. At the time, weight status was not only positioned as an outcome of the model but was also noted to reciprocally influence the continued development of the other variables within the model. The original model was based upon piecemeal evidence requiring more systematic empirical evidence to determine support for and the strength of the proposed pathways.

**Fig. 2:**
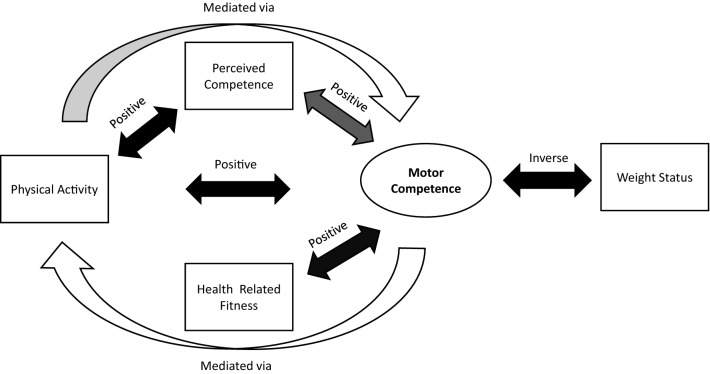
Research consensus on motor competence and health-related variables. *Black arrow* indicates extensively tested: consistent relationship; *dark grey arrow* indicates moderately tested: variable relationship; *partial grey arrow* indicates partially tested: some evidence; *white arrow* indicates limited testing. The direction of the relationship is indicated above the arrows. Reproduced from Robinson et al. [5] with permission

In 2015, a narrative review was published to synthesise evidence related to the original model, and a figure was produced to summarise the available evidence at this time (see Fig. 2) [5]. The results of this review provided convincing evidence that MC was positively associated with PA, cardiorespiratory fitness, muscular strength, muscular endurance and healthy weight status (denoted by black arrows in Fig. 2). However, this review was limited methodologically as it was not systematically conducted, and, whilst evidence for a positive association between MC and both PA and health-related fitness was reported [5], the causal directionality of this relationship was not clear because of the limited number of longitudinal or experimental studies [6–10]. In Fig. 2, grey and white arrows indicate the pathways with insufficient or inconclusive support to confirm the proposed hypotheses (e.g., mediating effects of health-related fitness and perceived MC [5, 11]). In that narrative review [5], the operational definition of MC was expanded to encompass both motor skills (locomotor, object control/manipulative, and balance skills) and their underlying mechanisms (capabilities such as motor coordination). This overarching term is used in the current review, and we focus on objectively assessed gross MC incorporating fundamental motor/movement skills or motor coordination.

Since 2015, the number of publications focusing on childhood MC has continued to grow exponentially [12], with many systematic reviews regarding MC and its links with PA, healthy weight status, fitness and perceived MC [13–23]. However, the evidence base remains limited. Only four of these 11 reviews had an expansive definition of MC [16, 17, 21, 24]. When considering the PA pathway, authors of a previous review suggested that it is important to investigate the relationship between different types and intensities of PA with skill domains (e.g., locomotor, manipulative and stability) as the relationship between PA and gross MC is not straightforward [24]. Yet, many reviews regarding PA and MC have not systematically addressed associations by skill domain [5, 13, 16, 25]. Only four reviews examined evidence for the role of the different components of MC on PA [26, 27], perceived MC [21] and all variables in the model [24]. Furthermore, only four reviews focused on longitudinal and/or experimental evidence [13–15, 23]. Only one review article [24] captured all the relevant variables pertaining to MC [2] but only investigated MC as the outcome variable, thus not exploring many of the model pathways or mediating mechanisms. Finally, no review comprehensively addressed the mediating pathways. To date, no review article has provided a full picture of current evidence for this model.

To advance knowledge on the relationship between children’s and adolescents’ MC development and PA, health-related fitness, weight status and perceived MC, a synthesis is required that uses a broad definition of MC and considers the role of different types of MC, prioritises high-level evidence (i.e., longitudinal and experimental research) and considers all variables in the model that relate to MC. Therefore, the purpose of the current review was to systematically compile longitudinal, experimental and mediation (both longitudinal and cross sectional) evidence from late 2014 relevant to the Stodden et al. [2] model and provide an update of the Robinson et al. [5] review. MC is central to the model by Stodden et al. [2]. As such, all pathways to and from MC are considered. The pathways in the model that do not directly link to MC (i.e., ‘perceived MC and PA’ and ‘fitness and PA’) are beyond the scope of this review and are not considered.

## Methods

### Identification of Studies and Search Strategy

The review was submitted to PROSPERO on 22 January 2020 (ID CRD42020155799) and registered on 28 April 2020. The searches were performed on 30/31 October and 8 November 2019. Searches focused on articles published from 2014 to 2019 (i.e., articles published since the narrative review by Robinson et al. [5]). Searches (using single and combined terms) were initially conducted for each of the following databases separately: CINAHL Complete, ERIC, Medline (OVID), PsycINFO, Web of Science Core Collection, Scopus and SportDiscus. See Table 1 in the electronic supplementary material (ESM) for specific search terms.

### Inclusion and Exclusion Criteria

Two screeners at each stage determined whether the article met the initial inclusion criteria then ascertained whether the studies assessed variables relevant to the Stodden et al. [2] model. Finally, screeners assessed whether the study met the study design and analysis criteria. See Table 1 for specifics.

**Table 1:** Inclusion and exclusion criteria

Inclusion	Exclusion
***Step 1: Assess study meets following initial criteria***	
Human studies	Animal studies
Original, peer-reviewed research	Abstracts, reviews, protocols, commentaries, methods/validity studies
Published in English or languages of the author group: Dutch, German, Italian, Portuguese, Spanish	Published in a language the author group could not read
Age ≥ 2 and ≤ 18 years	Infants or aged > 18 years
Typically developing	Non-typically developing
Non-special population (except if it is a tracking study that has typically developing children and analysis has been done on this group). Children from low socioeconomic areas are included	Special population (e.g., disability, cancer, athletes, obese) without a comparator group
***Step 2: Assess variables relevant to Stodden *et al*. ***[2]*** model***	
Measure of gross MC (assessed objectively not via self-report) that can include fundamental motor/movement skills, motor coordination or other goal-directed movement. Combined measures that include fine and gross motor skills can be included if relationships with two or more gross motor skills can be extracted	Manual dexterity (on its own) not considered gross MC. Context-/sport-specific skills (e.g., judo, soccer skills). Single skill assessments (e.g., developmental sequences). Measures that are termed motor skills but primarily assess attributes of fitness (e.g., agility, power, aerobic capacity)
Studies need to examine more than one variable within the model [2] by testing at least one pathway. Pathways must include the variable MC	Pathways that do not include MC, i.e., perceived MC to PA and fitness to PA
If fitness, studies that report on health-related fitness [3], i.e., overall fitness measures or cardiovascular endurance, muscular strength/endurance, flexibility. Standing long jump was considered a measure of muscular strength rather than skill	Agility (short shuttle runs, e.g., 4 × 10 m) and speed tests (e.g., dash)
If physical activity, studies that report on PA intensity or type, both objective and subjective measures. Sport participation can be included as a form of PA participation	Sport-specific population studies (e.g., football participants)
If perceived MC, studies that define the construct as perceived MC, perceived sport competence, physical self-perception or physical self-confidence	Self-esteem, self-efficacy, self-concept, global self-worth. Note: These terms were searched for in case authors used these terms when assessing the narrower concept of perceived MC
***Step 3a: Assess study design and analysis***	
Longitudinal or experimental studies (including quasi- experimental)	Qualitative, case study. Cross-sectional studies re-considered for step 3b
Longitudinal studies: Measures of MC AND at least one other variable relevant to the model. At least two different time points, but this does not mean each variable needs to be assessed twice. Analysis can answer the question of how MC is associated with at least one other variable in the model	Measures of MC but not measures of another relevant variable. Analysis does not answer the question of how MC is associated with another variable in the model (e.g., tracking study of typically developing children and children with a disability and analysis compares how groups differ according to fitness and skill rather than examining how these variables are associated)
Experimental studies: Measures of MC at least at two different time points (e.g., pre and post). Analysis can answer the question of whether manipulating MC contributes to change in the other variable (or vice versa)	Analysis cannot determine a causal relationship between MC and the other variable(s) (or vice versa)
***Step 3b: If cross sectional, assess additional criteria***	
Investigates comprehensive—mediated and moderated—models linking MC to more than one target variable within the model	Does not address mediation in fitness and/or perceived MC in the way the variables are operationalised in the model [2]

*MC* motor competence, *PA* physical activity

### Search Process

In stage 1, each search was imported into Covidence systematic review software (Veritas Health Innovation, Melbourne, Australia; www.covidence.org) as separate reviews (i.e., PA, weight status, perceived MC and fitness, each coupled with the MC construct). Four authors carried out the initial abstract and title screening for each of the four reviews independently as ‘the first screener’. An additional two authors (with particular subject expertise) assisted in each review as the ‘second screener’. The purpose of this first screening was to identify any potentially relevant article. A combined total of 585 articles were located for full-text screening. Two authors then independently screened each full-text article. Any conflicts were resolved in meetings with at least three authors to reach consensus. A total of 336 articles proceeded to stage 2 (249 excluded). These 336 articles were the result of included studies for each review (i.e., PA articles, weight status articles, etc.). These were then combined into one file, and duplicates (*n* = 177) and any references that had been included in the narrative review [5] (*n* = 7) were removed, leaving 152 studies. In this final review, the core methods team (and two other authors for non-English papers) completed independent full-text screening to identify the 42 articles eventually extracted. One additional article was identified as a reviewer suggestion during the review process, making a total of 43 (see Table 2).

**Table 2:**
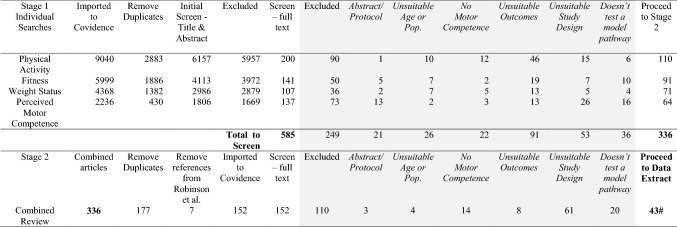
Prisma search results

Grey shading indicates the papers excluded at each stage

^#^One additional paper was identified during the review process

### Risk-of-Bias Assessment

Risk of bias was assessed via the National Institutes of Health’s quality assessment tools for controlled intervention studies and for observational cohort and cross-sectional studies (also used for mediation studies), respectively [28]. Both quality assessment tools include 14 criteria to assess a study’s risk of bias. All the criteria were scored with a ‘yes’ (i.e., criterion was met; low risk of bias), a ‘no’ (i.e., criterion was not met; high risk of bias), or a ‘could not be determined (CD)’. The ‘CD’ items were also considered as potentially high risk when reporting results. The complete risk-of-bias assessment procedure consisted of three steps. First, three authors independently assessed the 14 criteria of the same three papers and sent their results to a fourth author who compiled an overview of the assessments. This overview was subsequently discussed with the four above-mentioned authors and two other authors. Differences in assessment were resolved to ensure consistency in assessing the remaining papers. Second, 30 papers were each assessed by two of three authors (i.e., each of the three authors assessed 20 papers), and any inconsistencies in assessments were checked by the respective third author. In a random sample of 11 papers (14 items each paper), a high level of agreement was obtained [29], i.e., two raters assigned to each paper agreed with 76% (117/154) of the quality items. As such, in the third and final step, the remaining papers (*n* = 10) were assessed by one author of the team of three; in case of doubt, they consulted the two other authors to come to an agreement.

### Data Extraction and Results Syntheses

Associations between MC and other variables (PA, weight status, health-related fitness, perceived MC) were extracted and sorted into tables based upon the relevant pathway and the study type (longitudinal, experimental, mediation; see Tables 2–7 in the ESM).

Effect sizes were calculated where possible using relevant freely available effect size calculators (https://www.danielsoper.com/statcalc/default.aspx) appropriate to the analyses and data provided in the articles (i.e., effect size calculator for standardized regression coefficient or unstandardized beta, multiple regression, correlation, *F* test, *T* test). If authors reported odds ratios, these were recorded without interpretation. For mediation analysis, conventional guidelines for interpretation of the size of the indirect effect have not been established [30, 31], so the calculated indirect effect was reported (where it could be calculated). The information used to calculate effect sizes is available from the authors on request.

Summary tables were produced for PA, health-related fitness, perceived MC, and weight status by associations for each pathway (both directions) and each skill domain (total, object control, locomotor/coordination/stability/balance). The study design (longitudinal/experimental/ cross-sectional mediation), MC assessment type (product/process), fitness domain (e.g., endurance), PA measure (measurement [objective/subjective], time period [e.g., weekday], intensity [e.g., moderate]), perceived MC measure (instrument and whether it was aligned [or not] with the actual MC assessment [4]) was highlighted in the respective tables. Calculated effect sizes were interpreted according to commonly used conventions (e.g., Cohen’s d) and described as small, medium or large effects in the summary tables. These were not formally summarised in the summary tables because of the lack of data but are referred to in the results where relevant.

Results syntheses were performed qualitatively to determine the level of evidence. A ‘final’ result for level of evidence in support of each pathway and each pathway direction (and each skill domain if there were at least three studies) was obtained in accordance with methodology used in previous correlates reviews [24, 32]. In accordance with this methodology, percentages in parentheses refer to the number of analyses finding a significant association in the hypothesised direction divided by the total number of analyses, including those that found either a null effect or an association not in the direction of the hypothesised pathway. This was done overall for all studies testing a pathway (e.g., weight status) and for skill subdomains within that pathway (e.g., object control). Based on the percentage of findings supporting the respective association, the variable was classified as either no association (0–33%), written as ‘0’; indeterminate/inconsistent (34–59%), written as ‘?’; or a positive ‘+’ or negative ‘−’ association (≥ 60%). When four or more studies found an association, and the association was > 60%, it was classified as ‘++’ or ‘−−’ accordingly. If there were a total of three or fewer studies in the domain of interest, the strength of evidence was considered insufficient (I) to classify.

In cases where domains of interest included studies with multiple analyses in one study (studies with more than eight analyses), an additional calculation was provided excluding such studies so as not to skew results. For example, if one study reported ten analyses examining object control skill and aspects of PA (e.g., total PA, light PA, moderate PA, and vigorous PA, etc.) and only one of the analyses was significant, the ratio would be 1/10. Five other studies in the skill domain of object control may have reported one significant PA–object control analysis each. In total for this domain, there would be 15 results with six being significant (i.e., 40% [6/15]), which would be considered indeterminate evidence (‘?’). If we excluded the study with the ten analyses, the overall result for that domain of object control skill and PA would become positive (i.e., 100% [5/5]) and therefore classified as strong positive (‘++’) evidence. In this example, the study with ten analyses contributed more ‘weight’ to the summary score, potentially biasing results.

## Results

### Risk-of-Bias Assessment

Risk of bias was based on 14 criteria (see Table 8 in the ESM). With regard to intervention studies (*n* = 4), the most frequently biased item (100% of studies either being at high risk of bias or not providing sufficient information to determine the risk of bias) related to blinding (i.e., of participants and treatment providers [item 4] and of outcome assessors [item 5]). Blinding of providers is impractical for interventions targeted to improve MC that require specific teaching expertise and are often led in an ecological school learning context either by external specialist teachers or by trained generalist teachers. The majority of studies (75%) had no clear statement regarding the adherence of the treatment groups to the intervention protocols (potential bias of item 9). Other items that were biased in 50% of the intervention studies included concealed treatment allocation (item 3), similarity between the intervention and control group at baseline (item 6) and sufficient sample size (item 12).

For mediation studies (*n* = 10), six items were considered ‘high’ risk in most of the studies. Given the nature of the study design (e.g., seven of the ten mediation studies were cross-sectional), the exposures of interest could not be measured before the outcomes (item 6) or more than once over time (item 10) with a sufficient timeframe, so that one could reasonably expect to see a longitudinal association between exposure and outcome if it existed (item 7). In eight papers, the loss to follow-up after baseline was either more than 20% or could not be determined (item 13). Similarly, in nine papers, it was either unclear whether the outcome assessors were blinded to the exposure status of participants or it was stated that they were not (item 12). In seven of the ten papers, the participation rate of eligible people could not be determined or was less than 50% (item 3).

With respect to longitudinal studies (*n* = 32), of which three were also included in the overview of the mediation studies, the most frequently biased items were item 12 (i.e., blinding of the outcome assessors to the exposure status of participants [81%]), item 3 (i.e., the participation rate of eligible people being at least 50% [59%]), item 13 (i.e., the loss to follow-up after baseline being ≤ 20% [59%]) and item 5 (i.e., provision of a sample size justification, power description, or variance and effect estimates [50%]).

### The Pathway from Physical Activity (PA) to Motor Competence (MC) or the Reverse

#### PA to MC

Eleven studies [33–43] with 98 different analyses (many studies had more than one analysis) investigated the pathway from PA to MC (see Table 3 for the number of analyses within each study). With no evidence to support a positive association, with only 8/98 analyses significant (8%), this was rated as no association (‘0’). For many studies, effect sizes could not be calculated. In studies where effects could be calculated, most effects were small. Antunes et al. [42] reported only one significant result (with a large effect size) from 72 analyses; as such, results from this study skewed the overall picture. However, even after excluding Antunes et al. [42], there was still no evidence to support a positive association for a pathway from PA to MC (27% [7/26], rated as no association [‘0’]; Table 3).

**Table 3:**
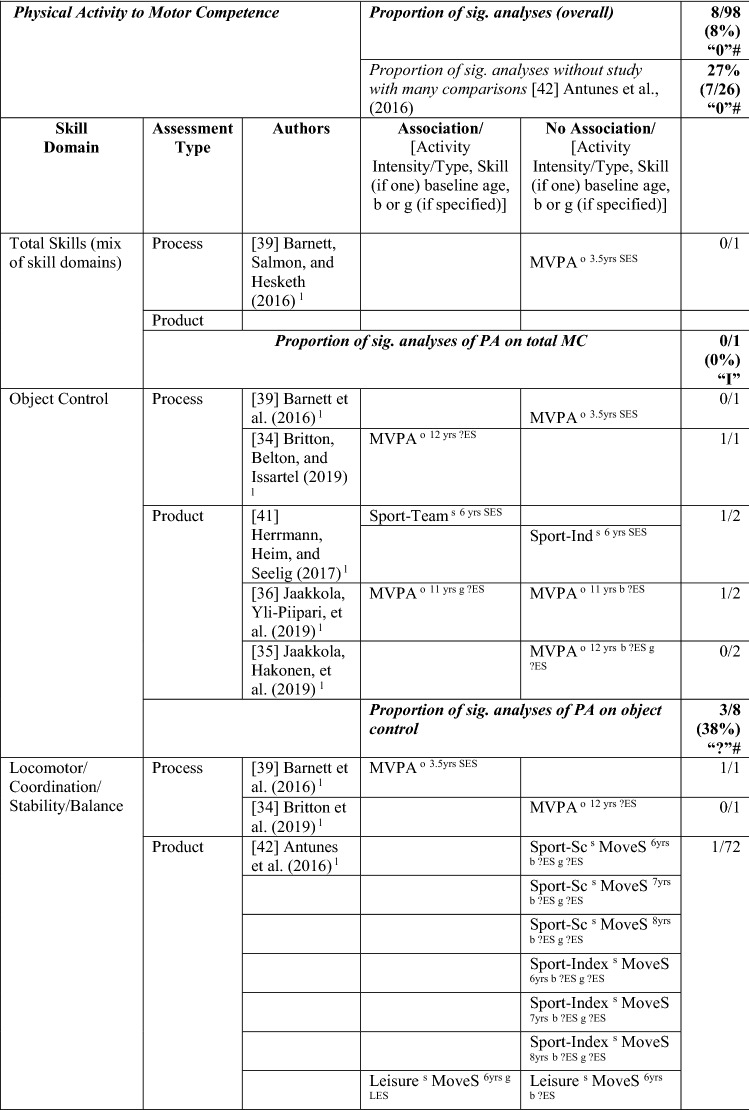

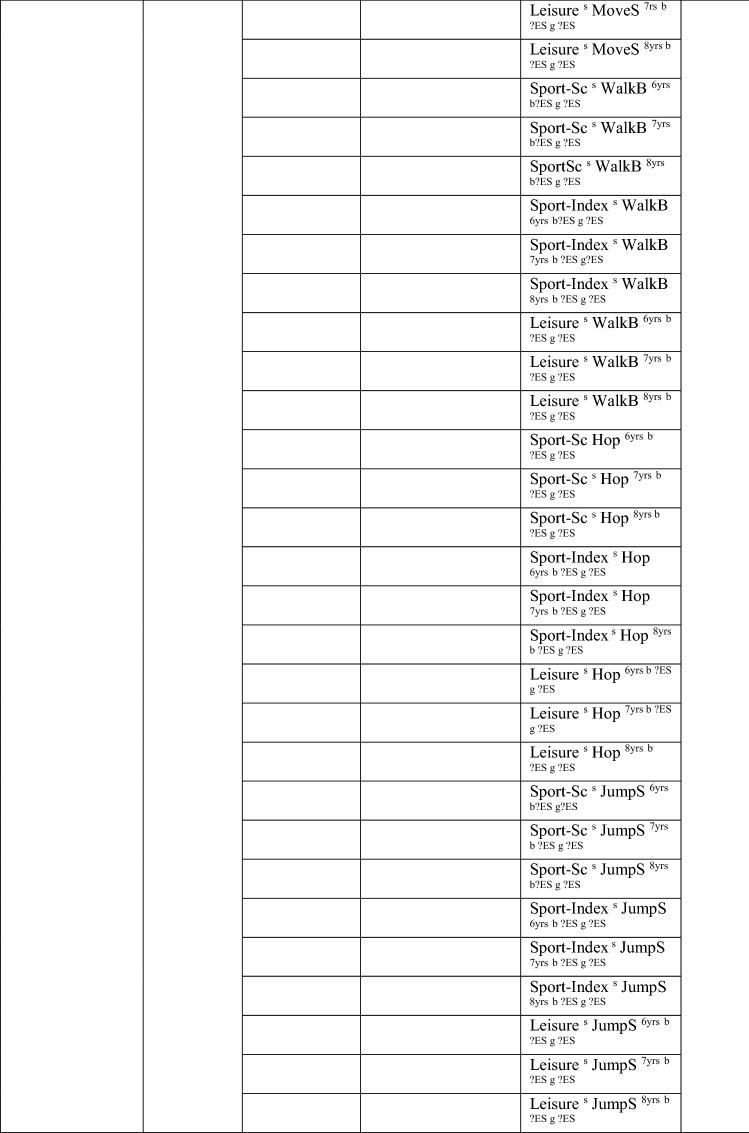

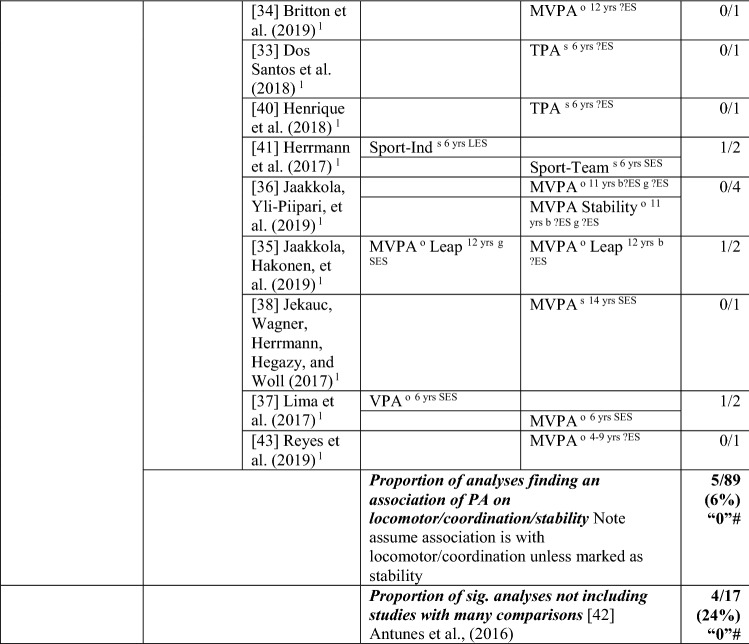
Summary of studies according to the pathway physical activity to motor competence

If significant for whole sample, gender differences are not presented; adjusted values are used to report significance when they are reported; if only one skill is tested, it is identified

*boys* boys only, *girls* girls only, *JumpS* jump sideways, *L* longitudinal, *LES* large effect size, *MoveS* move sideways, *MVPA* moderate to vigorous PA, *OPA* objective PA, *PA* physical activity, *SES* small effect size, *SPA* subjective PA, *Sport-Ind* sport individual, *Sport-Index* sum of sport score divided by four, *Sport-Sc* sport score, *Sport-Team* sport team, *TPA* total PA, *VPA* vigorous PA, *WalkB* walk backwards, *?ES* effect size could not be calculated due to lack of information

^a^Based on the percentage of findings supporting the association, the variable was classified as either no association (0–33%), written as ‘0’; indeterminate/inconsistent (34–59%), written as ‘?’; or a positive ‘+’ or negative ‘−’ association (≥ 60%). When four or more studies found an association, it was classified as ‘++’ or ‘−−’ accordingly. If there were three or fewer studies in the domain, the strength of evidence was considered insufficient (I) to classify

Only one study investigated PA as a predictor of total MC. In this study, the objective measure of moderate to vigorous physical activity (MVPA) at 3.5 years was not predictive of total MC (process-oriented assessment) in 5-year-old Australian children [39].

Eight analyses (five studies) assessed PA as a predictor of object control skills. Evidence was not sufficient to support this pathway (38% [3/8], rated as indeterminate [‘?’]). Three of eight analyses showed a positive association, although these varied by sex and activity intensity/type. Two studies reported MVPA (objectively measured) as a predictor [34, 36], yet one study reported an association for girls but not boys [36]. Furthermore, one study reported team-based sport was a predictor but individual-based sport was not [41]. The study of young Australian children did not report an association between MVPA and object control skills [39]. Another study in Finnish children reported no association between MVPA (objectively measured) and object control skills after 1 year in either girls or boys (aged 12 years) [35].

Many analyses examined locomotor/coordination/stability skills but failed to support that pathway, as only 6% (or 24% without the study examining 72 potential associations [42]) reported an association. MVPA was a significant predictor of locomotor skills (process assessment) at the age of 5 for Australian children aged 3.5 years at the initial measurement [39]. In 6-year-old German children followed up after 8 months, subjectively assessed PA (participation in individual sports training and frequency of practice, rather than team-based sports) predicted locomotor and stability skills [41]. In 6-year-old Danish children, a longitudinal study over three time points (ending when children were aged 13 years) reported that objectively measured (accelerometer) vigorous PA was directly associated with coordination (product assessment) [37]. However, no direct association between MVPA and coordination was observed in the same study [37]. Other studies in 6-year-old Portuguese children reported no associations between PA and MC. Leisure-time PA predicted moving to the side (a product assessment) at the age of 12 years for girls, whereas it did not predict the other tested coordination skills for boys or girls, and the sport score or sport index did not predict any of the four tested coordination skills [42]. Two studies using subjective assessments of total PA reported no association between PA and MC 3 years later at the age of 9 years [40] or association with change over 4 years in MC [33]. Another study in Portuguese children (aged 4–9 years at baseline), this time using an objective measurement of MVPA, also reported PA was not a significant predictor of motor coordination in boys and girls across a 3-year period [43].

In older children and youth, two studies using objectively measured MVPA reported subsequent associations with specific skills after 1 year, but these varied according to sex and MC measure. In Finnish grade six children, PA was positively associated with leaping at grade seven for girls but not for boys [35]. In Finnish children of a similar age (11 years), MVPA was not predictive of coordination (product assessment) for either sex 1 year later [36]. For 12-year-old Irish children, MVPA was not associated with the process or product assessment of locomotor/coordination skills 1 year later [34].

Overall, we found no evidence for the pathway from PA to MC. When considering skill domains, there was no evidence for a pathway from PA to total skills (insufficient studies) or locomotor, coordination and stability skills, but there was indeterminate evidence for object control skills.

#### MC to PA

The pathway from MC to PA [34–38, 44–58] was investigated in 20 studies, with 26% (32/123) of analyses positive, from at least one domain of MC to PA. Bryant et al. [48] analysed 60 potential associations and found only six to be significant; as such, results from this study skewed the overall picture. Without considering this study, the overall proportion of analyses that were statistically significant was 41% (26/63), so the level of evidence was indeterminate (‘?’). For analyses where an effect size could be calculated, the effects ranged from small to large (see Table 4).

**Table 4:**
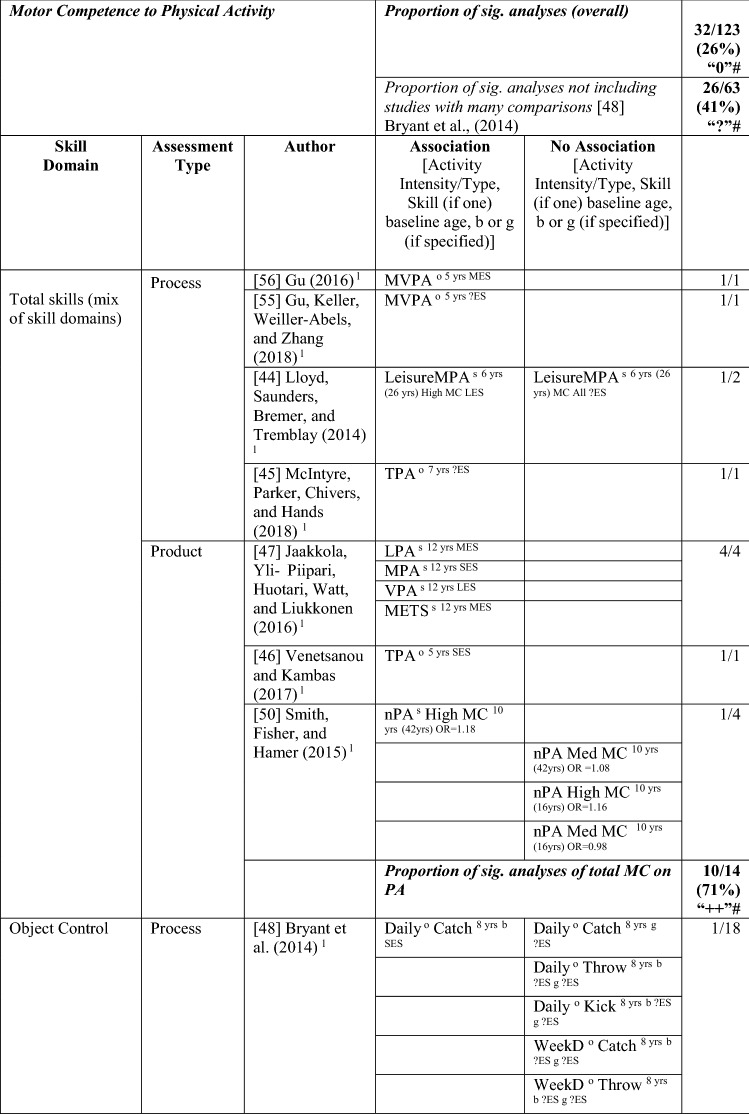

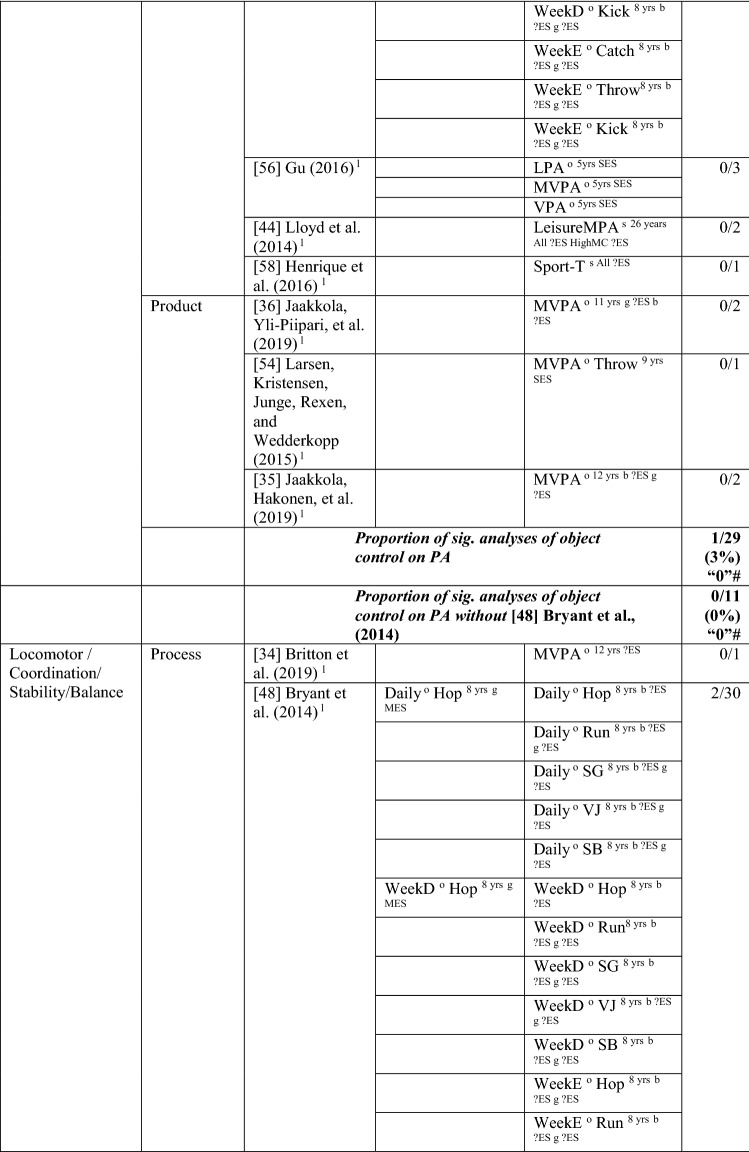

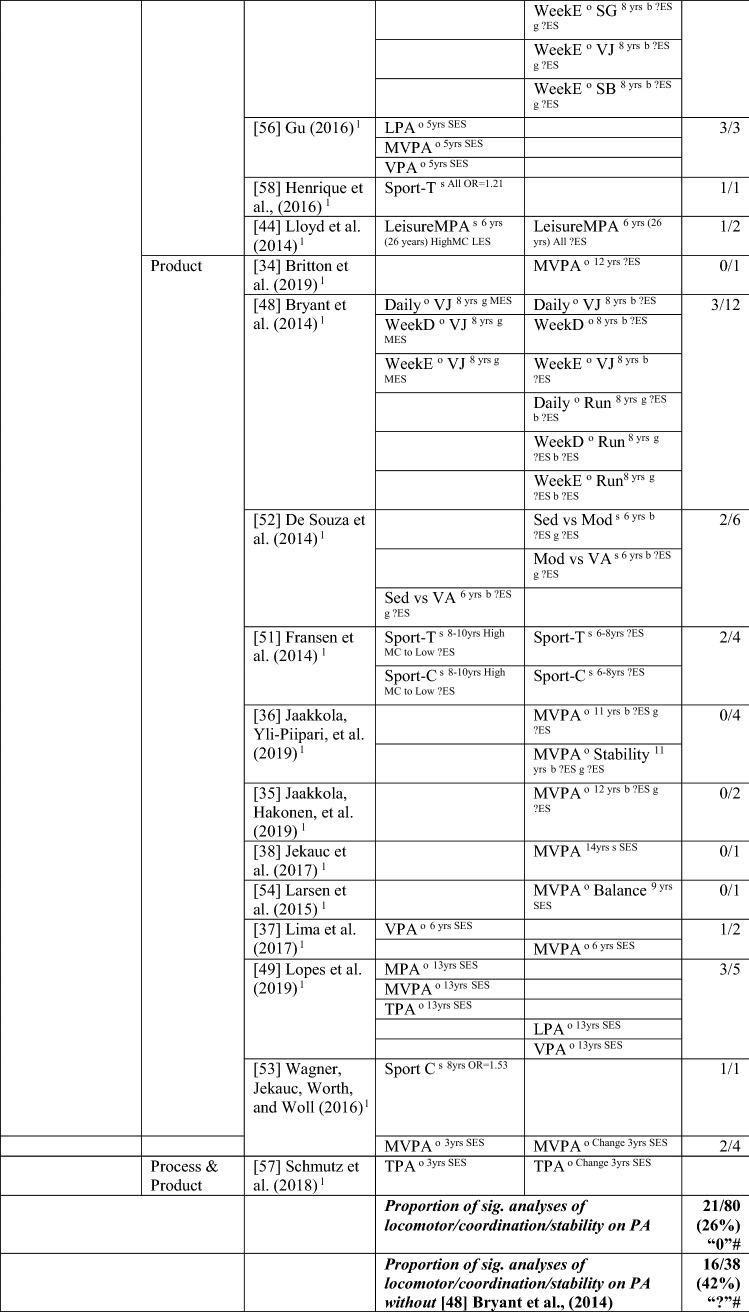
Summary of studies according to the pathway motor competence to physical activity

If significant for whole sample, gender differences are not presented; adjusted values are used to report significance when they are reported; if only one skill is tested, it is identified

*HMC* high MC, *L* longitudinal, *LES* large effect size, *LPA* light PA, *MC* motor competence, *MES* medium effect size, *METS* metabolic equivalent, *MMC* medium MC, *Mod* moderate, *MPA* moderate PA, *MVPA* moderate to vigorous PA, *nPA* number of PAs, *OPA* objective PA, *OR* odds ratio as reported by authors, *PA* physical activity, *SB* static balance, *Sed* sedentary, *SES* small effect size, *SG* side gallop, *SPA* subjective PA, *Sport-C* sport club, *Sport-T* sport total, *TPA* total PA, *VA* very active, *VJ* vertical jump, *VPA* vigorous PA, *WeekD* weekday, *WeekE* weekend, *?ES* effect size could not be calculated because of lack of information

^a^Based on the percentage of findings supporting the association, the variable was classified as either no association (0–33%), written as ‘0’; indeterminate/inconsistent (34–59%), written as ‘?’; or a positive ‘+’ or negative ‘−’ association (≥ 60%). When four or more studies found an association, it was classified as ‘++’ or ‘−−’ accordingly. If there were three or fewer studies in the domain, the strength of evidence was considered insufficient (I) to classify

Ten of 14 analyses (seven studies) reported positive associations between total MC and PA (71%); as such, the level of evidence for this pathway was strong (‘++’). Around half of these studies used process measures [45, 55, 56] such as the Test of Gross Motor Development [44], and half used product measures [47, 50] such as the Bruininks-Oseretsky Test of Motor Proficiency [46]. Total MC was a predictor of different PA intensities, such as light [47], moderate [47] (measured subjectively) MVPA [55, 56] (measured objectively) and vigorous PA [47]. In the youngest children of these analyses, Gu et al. [55] and Gu [56] reported that total MC (process assessment) in American 5-year-olds predicted objectively measured MVPA 1 year later.

Four studies assessed total MC as a predictor of either total PA [45, 46] or number of PAs/leisure [44, 50], with four of the eight analyses significant and positive. In Greek children, total MC (product assessment) at the age of 5 years predicted total activity (step count) 10 years later at 14.5 years [46]. In Australian children, MC (process assessment) assessed at age 7 years was a predictor of total activity (step count) 18 months later [45]. In a Canadian study with four time points, total MC at the age of 6 years (process assessment) was a predictor of total activity at the age of 26 years for those with high MC [44]. In a study from the UK with three time points, MC (product assessment) at the age of 10 years was associated with PA (subjectively reported total activity) at the age of 42 years for those with high MC at baseline [50] but not for those with medium levels of MC. MC at age 10 years was not predictive of total activity at age 16 years.

A total of 29 analyses, with a mix of process/product assessments conducted as part of seven studies [35, 36, 44, 48, 54, 56, 58], investigated object control skills as a predictor of PA, with only one significant analysis (using a process assessment). As such, there was no evidence (3%) for this pathway, even without the study by Bryant et al. [48], which had multiple analyses (0% [0/11], rated as no association [‘0’]). Bryant et al. [48] reported that, for 8-year-old boys, the catch was predictive of daily PA 1 year later, with no relationships reported for the other analyses (throw or kick for boys or girls on weekday or weekend activity). In other studies using process measures, object control skills at the age of 5 years did not predict light PA, MVPA or vigorous PA 1 year later in American children [56], and object control skills at the age of 6 years was not a predictor of leisure time moderate activity at the age of 26 years for those with high MC [44]. In older children, throwing (product assessment) did not predict MVPA (accelerometer) at the age of 9 years over 3 years [54] or 1 year later in 11- and 12-year-old Finnish children [35, 36].

In total, 15 studies with 80 analyses reported 21 significant associations for a pathway from locomotor/coordination/stability skills to PA, which did not provide evidence for this pathway (26%). Without the study by Bryant et al. [48], the level of evidence can be considered as indeterminate (42% [16/38]). This skill domain was investigated as a predictor of total PA by five studies. In 3-year-old Swiss children, a composite (process and product) locomotor score was associated with total PA 1 year later; however, these skills were not a predictor for the change in total PA shown across this 1-year period [57]. In Brazilian 4-year-olds, locomotor skill predicted organised PA (described as sport participation) 2 years later [58]. In a Canadian study with four time points, locomotor skills at the age of 6 years (process assessment) predicted total PA at the age of 26 years [44]. In 9-year-old children from the UK, Bryant et al. [48] conducted several analyses in boys and girls separately. In girls, hopping (process assessment) and jumping (product assessment) were predictive of total PA (measured using a pedometer) 1 year later [48], whereas catching was a predictor in boys [48]. There were no reported relationships for other skills in either boys or girls (process assessments: run, side gallop, vertical jump, throw, kick; product assessment: static balance) [48].

In terms of pathways from locomotor/coordination/stability to PA intensity, no clear pattern existed. In 3-year-old Swiss children, a composite locomotor score was associated with MVPA (objective) 1 year later; however these skills were not a predictor for change in MVPA levels [57]. In 5-year-old American children, locomotor skills (process assessment) predicted objectively measured light PA, MVPA and vigorous PA 1 year later [56]. In 6-year-old Danish children, locomotor (product assessment) skills predicted vigorous PA (objectively measured) at the age of 13 years [37]. In Portuguese children, MC (product assessment) at 13 years predicted objectively measured MVPA, moderate PA, and total physical activities (but not light PA or vigorous PA), 1 year later [49]. In contrast, five other analyses of MVPA (mostly objectively assessed [34, 35, 37, 54]), did not report a direct association [34, 35, 37, 38, 54], although Lima et al. [37] did report a mediated effect. The starting age at baseline and the follow-up period varied (i.e., coordination at 6 years and MVPA at 13 years [37], balance at 9 years and MVPA at 12 years [54], coordination at 11 years and MVPA at 12 years [35], coordination at 12 years and MVPA at 13 years [34], coordination at 14 years and MVPA at 20 years [38]). Finally, in Portuguese children, locomotor skills (product assessment) were lower in 6-year-olds for both boys and girls who were classified as sedentary (vs. very active) at 10 years old [52].

In addition, there was no clear pattern between MC and sport participation. Locomotor skills (product assessment) in Brazilian 4-year-olds and German 8-year-olds were a predictor of, respectively, organised PA (described as sport participation) 2 years later [58] and sport (subjective measure of club sports participation) at 14 years old [53]. Instead, in a Belgian study, an association with sport (either total or club only) PA at 14 years was reported only for those aged 8–10 years and not for those aged 6–8 years.[51].

In summary, the evidence was indeterminate for the pathway from MC to PA. In terms of skill domains, there was strong positive evidence from total MC to PA but no evidence for object control skill to PA, and indeterminate evidence for locomotor/coordination/stability to PA.

#### MC and PA Interventions

Only two interventions fulfilled the inclusion criteria for addressing the causal relationship between MC and PA in either direction. Both studies used process measures of MC and objectively measured PA. Total MC (but not object control or locomotor competence) mediated the effect of a 12-month intervention on MVPA in Australian 8-year-olds [59]. However, MC improvement as a result of the 9-month intervention among 12-year-old Irish youth was significant and positive regardless of participant PA level [60], so intervention effects could not be attributed to PA. As such, there is insufficient evidence that MC interventions can promote change in PA or the reverse (see Table 5).

**Table 5:**
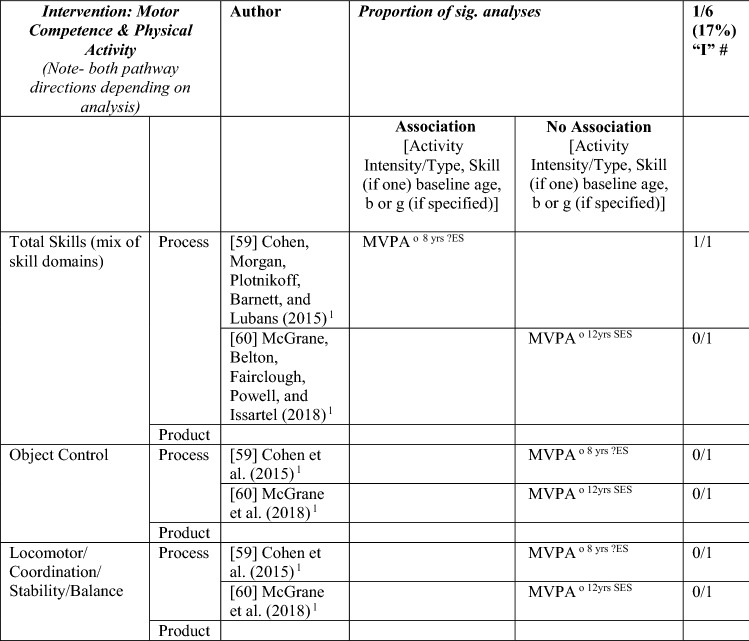
Summary of intervention studies – motor competence and physical activity

*L* longitudinal, *MVPA* moderate to vigorous PA, *OPA* objective PA, *PA* physical activity, *SES* small effect size, *?ES* effect size could not be calculated because of lack of information

^a^Based on the percentage of findings supporting the association, the variable was classified as either no association (0–33%), written as ‘0’; indeterminate/inconsistent (34–59%), written as ‘?’; or a positive ‘+’ or negative ‘ − ’ association (≥ 60%). When four or more studies found an association, it was classified as ‘++’ or ‘−−’ accordingly. If there were three or fewer studies in the domain, the strength of evidence was considered insufficient (I) to classify

### The Pathway from Weight Status to MC or the Reverse

#### Weight Status to MC

Nine studies investigated the pathway from weight status to MC, with around one-fifth of the analyses (22/98 [22%]) reporting a significant association in the hypothesised direction, rated as no evidence (‘0’). When studies with many comparisons were excluded [42, 48], the picture was quite different, with all analyses (10/10) indicating a significant negative relationship, rated as strong (‘—’). For most analyses, effect sizes could not be calculated. For the study with multiple comparisons, the few effects that were significant were large. See Table 6.

**Table 6:**
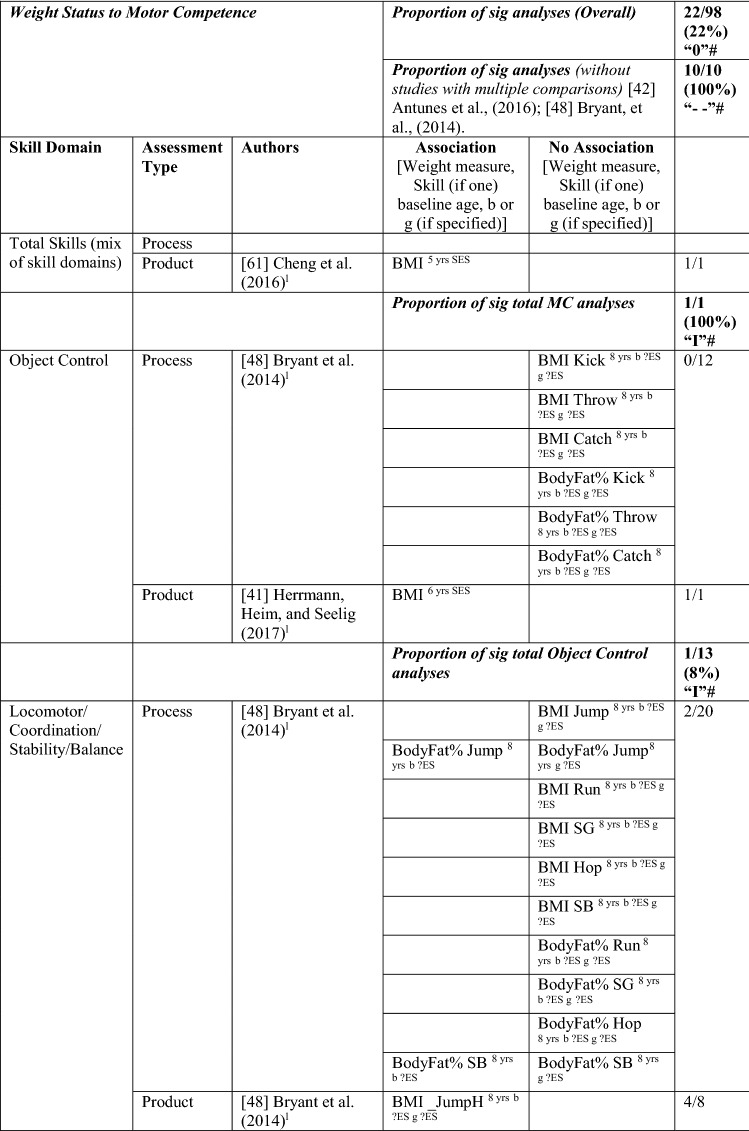

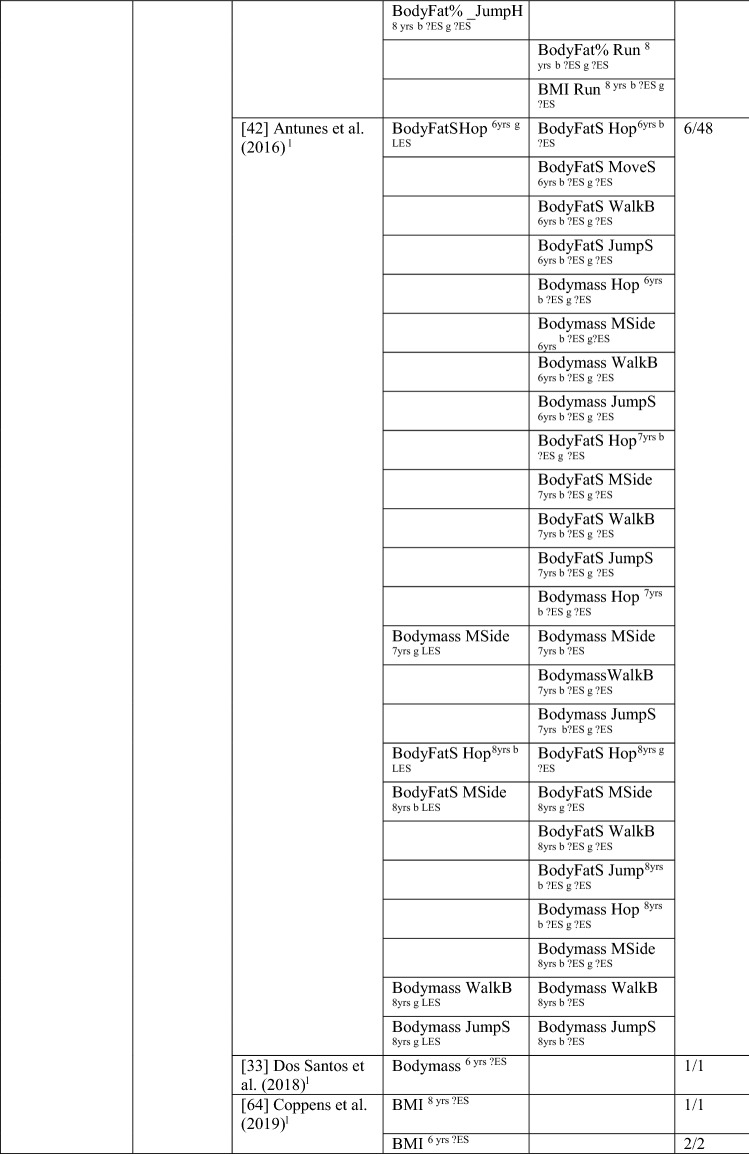

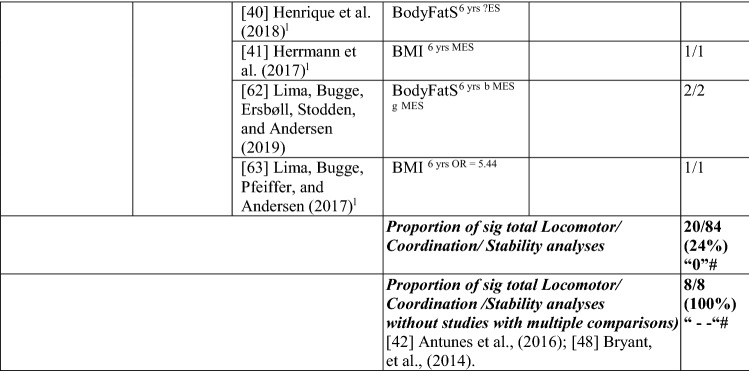
Summary of studies according to the pathway weight status to motor competence

If significant for whole sample, gender differences are not presented; adjusted values are used to report significance when they are reported; if only one skill is tested it is identified

*BMI* body mass index, *BodyFat*% skinfold assessment, *BodyFatS* sum of skinfolds, *Bodymass* weight, *Jump* vertical Jump with process measure, *JumpH* jump for height, *JumpS* jump to side, *L* longitudinal, *LES* large effect size, *MC* motor competence, *MES* medium effect size, *MoveS* move sideways, *OR* odds ratio as reported by author, *SB* static balance, *SES* small effect size, *SG* side gallop, *Waist* waist circumference, *WalkB* walk backwards, ?ES effect size could not be calculated because of lack of information

^a^Based on the percentage of findings supporting the association, the variable was classified as either no association (0–33%), written as ‘0’; indeterminate/inconsistent (34–59%), written as ‘?’; or a positive ‘+’ or negative ‘ − ’ association (≥ 60%). When four or more studies found an association, it was classified as ‘++’ or ‘−−’ accordingly. If there were three or fewer studies in the domain, the strength of evidence was considered insufficient (I) to classify

Cheng et al. [61] reported that Chilean children who were heavier at the age of 5 years had poorer total MC (product assessment) at 10 years. Bryant et al. [48] reported that British girls and boys with a higher body mass index (BMI) and greater body fat % did not have poorer object control skills (kick, throw, catch) 1 year later, whereas Herrmann et al. [41] reported that German children aged 6 years with a lower BMI had better object control (product assessment) scores 1 year later than those with a higher BMI.

Findings for locomotor/coordination/stability skills showed strong support for this pathway (100% [8/8], rated as strong negative [‘−−’]) when considered without the two studies with multiple comparisons [42, 48]. Six studies [33, 40–42, 62, 63] assessed weight status when children were 6 years old and reported associations with subsequent MC (product assessment). In the same sample of Portuguese children, Dos Santos et al. [33] reported that leaner children (body mass) had better motor coordination (product assessment) over the 3 years, and Henrique et al. [40] reported that children categorised as having lower BMI and less subcutaneous fat at 6 years old had high gross motor coordination levels at follow-up. German children with a lower BMI at age 6 years had higher skills (locomotor and stability) 1 year later. Danish children with a higher BMI at 6 years were more than five times more likely to be in the low MC group after 7 years (at age 13 years) than their lower BMI peers [63]. Antunes et al. [42] commenced with children aged 6, 7 and 8 years and assessed a range of skills (hop, moving to the side, walking backward, jumping to the side) in both boys and girls 6 years later and found that only six of 48 analyses were in the hypothesised direction.

In slightly older Flemish children, Coppens et al. [64] reported that a higher BMI at 8 years old predicted poorer motor coordination 2 years later. Bryant et al. [48] reported that a lower BMI at 8 years old was associated 1 year later with vertical jump (product assessment) for boys and girls. Lower body fat percentage at age 8 years was also associated with vertical jump (product assessment [girls and boys], and process assessment [boys]). Lower body fat percentage also predicted static balance in boys. However, the other skills in this domain, process assessments of side gallop, hop and sprint and both process/product assessments of the run, were not predicted by weight status for either boys or girls [48]. In summary, there is strong evidence of a negative relationship between weight status and MC.

#### MC to Weight Status

Five studies investigated the pathway from MC to weight status, with only 11% (5/45) of the analyses significant in the hypothesised direction, which means there was no evidence for this pathway (‘0’). The level of evidence was rated as strong negative (‘−−’), i.e., 80% (4/5), when one study with many comparisons was excluded [48]. Similar to the previous direction, effect size could not be calculated for most analyses. See Table 7.

**Table 7:**
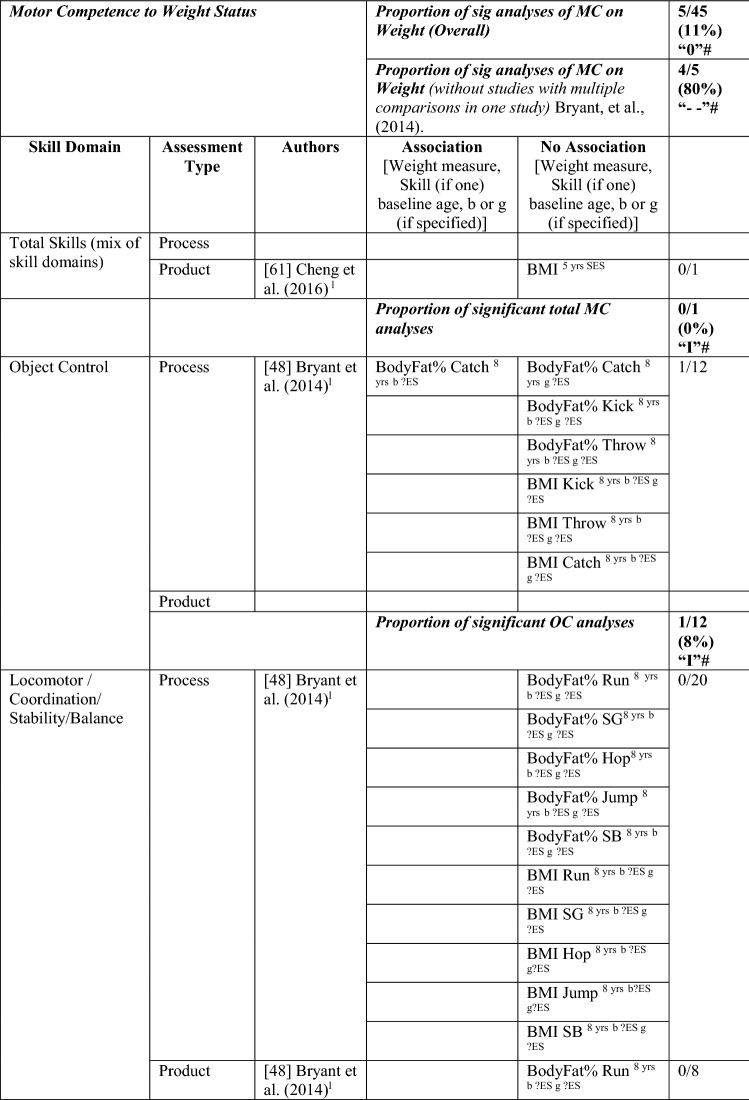

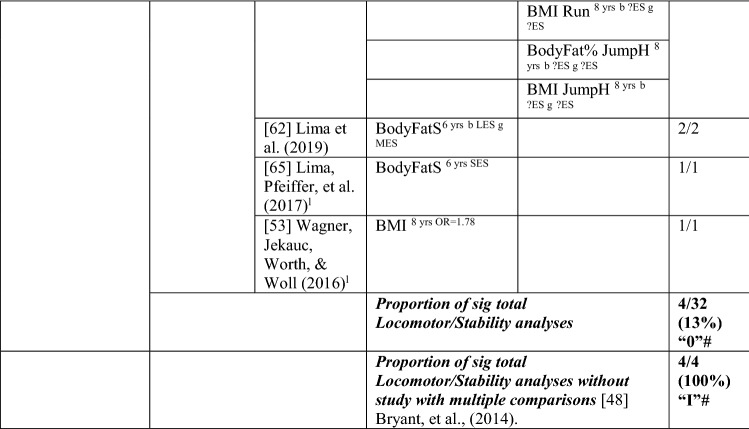
Summary of studies according to the pathway motor competence to weight status

If significant for whole sample, gender differences are not presented; adjusted values are used to report significant when they are reported; if only one skill is tested it is identified

*BMI* body mass index, *BodyFat*% skinfold assessment, *BodyFatS* sum of skinfolds, *Jump* vertical jump with process measure, *JumpH* jump for height, *L* longitudinal, *LES* large effect size, *MC* motor competence, *MES* medium effect size, *OC* object control, *OR* odds ratio as reported by author, *SB* static balance, *SES* small effect size, *SG* side gallop, *?ES* effect size could not be calculated because of lack of information

^a^Based on the percentage of findings supporting the association, the variable was classified as either no association (0–33%), written as ‘0’; indeterminate/inconsistent (34–59%), written as ‘?’; or a positive ‘+’ or negative ‘−’ association (≥ 60%). When four or more studies found an association, it was classified as ‘++’ or ‘−−’ accordingly. If there were three or fewer studies in the domain, the strength of evidence was considered insufficient (I) to classify

For total MC, Cheng et al. [61] reported that the MC level of Chilean children assessed at the aged of 5 years did not predict BMI at 10 years. Bryant et al. [48] investigated three object control skills (process assessment of catch, kick, throw) in British girls and boys over 1 year (aged 8–9 years) and reported no association with BMI or body fat percentage, except the catch (for boys), which predicted body fat percentage at age 9 years. Bryant et al. [48] assessed several locomotor and stability skills (process assessment of run, side gallop, hop, jump and static balance), with an additional product assessment for the run and the jump, and reported no associations with body fat percentage or BMI. In contrast, Lima et al. [62, 65] reported that Danish children with higher motor coordination at baseline (6 years old) had healthier levels of body fat, and Wagner et al. [53] reported that German children with low motor coordination at 8 years old were more likely to have a higher BMI in adolescence (14 years). Overall, there is no evidence for locomotor/coordination/stability as a predictor of weight status (4/32 [‘I’]). When the study with multiple comparisons was removed, four of four analyses were significant but only based on three studies, thus rated as insufficient ‘I’.

In summary, there is strong evidence of a negative association for weight status to MC and the reverse pathway, so long as studies with many multiple comparisons [42, 48] were excluded from the totals.

#### MC and Weight Status Interventions

Only one study assessed the impact of an MC intervention on weight status [60]. Whereas the 9-month intervention had positive and significant effects on MC in Irish 12-year-olds, weight status (i.e., normal weight or overweight/obese) did not have an impact on MC changes [60]. See Table 8.

**Table 8:**
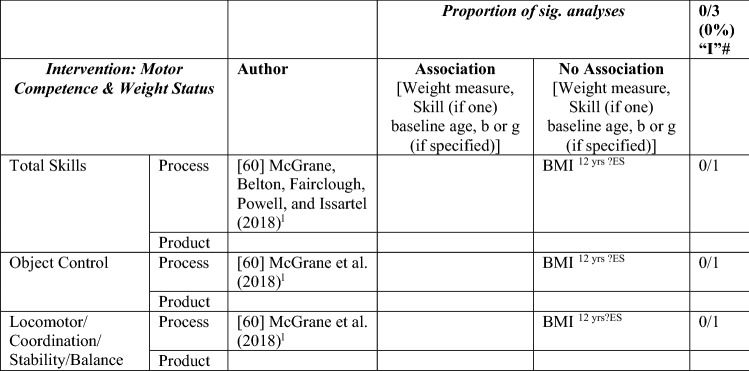
Summary of intervention studies – motor competence and weight status

*BMI* body mass index, *L* longitudinal, ?ES effect size could not be calculated because of lack of information

^a^Intervention study and was also listed as longitudinal

### The Pathway from Perceived MC to MC or the Reverse

#### Perceived MC to MC

There was insufficient evidence to support a longitudinal relationship between perceived MC and actual MC as we identified no studies that investigated this pathway direction.

#### MC to Perceived MC

Evidence was also insufficient to support a longitudinal relationship between actual MC and perceived MC. One study reported positive associations in two of six analyses (33%), rated as not enough information (‘I’). This Canadian longitudinal study investigated the pathway from actual MC (process assessment) at age 5–7 years to perceived MC (non-aligned recall assessment) at 16 and 26 years [44]. Total and object control skills (both large effect sizes) but not locomotor skills (small effect) were significantly and positively associated with perceived MC as an adolescent approximately 10 years later (baseline age ranged between 5 and 7 years). Neither object control nor locomotor skill domains were associated with perceived MC as an adult (recall measured at the age of 26 years), although total skills had a medium (non-significant) effect [44].

#### MC and Perceived MC Interventions

Two intervention studies investigated whether a motor skill intervention increased perceived MC [66, 67]. Lander et al. [66] used different measures of perceived physical competence in Australian preadolescents (12 years), one aligned and others not aligned with the process MC assessment. Even though perceived MC improved in both aligned and non-aligned measures after the intervention, this could not be attributed to actual MC changes in the intervention group. The 8-week intervention by Marouli et al. [67] in younger Greek children (4 years) (using a non-aligned measure of perception and a product assessment of MC) also did not result in a change in perceived MC. See Table 9.

**Table 9:**
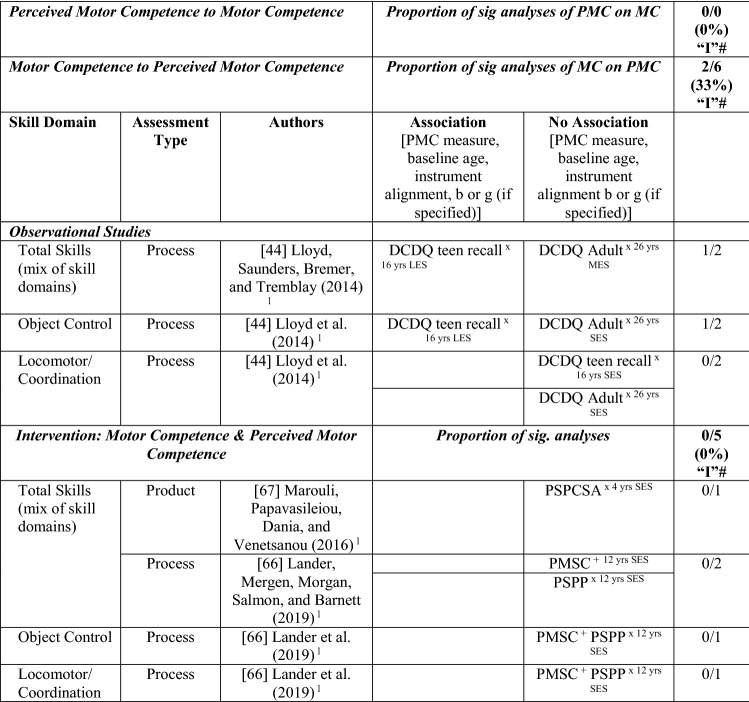
Summary of studies according to perceived motor competence

+ aligned actual and perceived measurements, *DCDQ* Developmental Coordination Disorder Questionnaire, *L* longitudinal, *LES* large effect size, *MC* motor competence, *MES* medium effect size, *PMC* perceived motor competence, *PMSC* Pictorial Scale of Perceived Movement Skill Competence, *PSPCSA* Pictorial Scale of Perceived Competence and Social Acceptance for Young Children, *PSPP* Physical Self-Perception Profile, *SES* small effect size, x non-aligned actual and perceived measurements, *?ES* effect size could not be calculated because of lack of information

^a^Based on the percentage of findings supporting the association, the variable was classified as either no association (0–33%), written as ‘0’; indeterminate/inconsistent (34–59%), written as ‘?’; or a positive ‘+’ or negative ‘−’ association (≥ 60%). When four or more studies found an association, it was classified as ‘++’ or ‘−−’ accordingly. If there were three or fewer studies in the domain, the strength of evidence was considered insufficient (I) to classify

Overall, there was no evidence for a pathway linking MC and perceived MC in either direction as evidence was inconsistent. This was likely because of the small number of longitudinal/intervention studies conducted in the past 5 years.

#### Perceived MC as a Mediator between MC and PA and the Reverse

Nine studies investigated perceived MC as a mediating variable, two using longitudinal data [34, 38], and the remainder using cross-sectional data [68–74]. One study [72] investigated the path from PA to perceived MC to MC only, four studies [68–71] investigated the path from MC to perceived MC to PA only, and four studies [34, 38, 73, 74] investigated both directions. See Table 10.

**Table 10:**
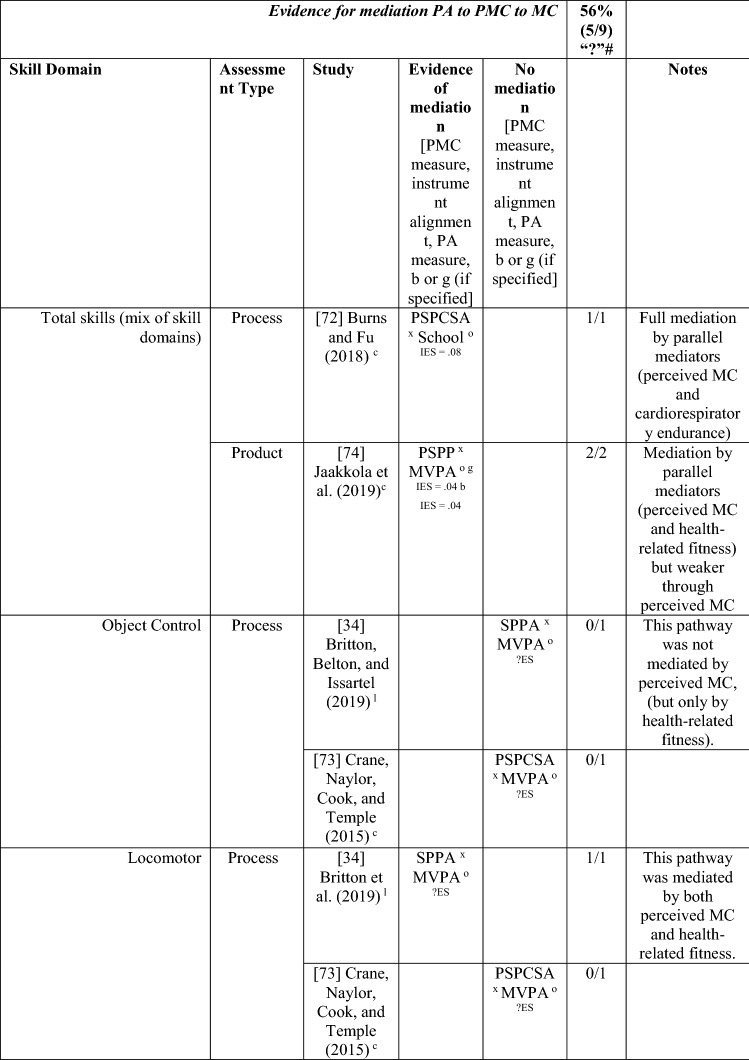

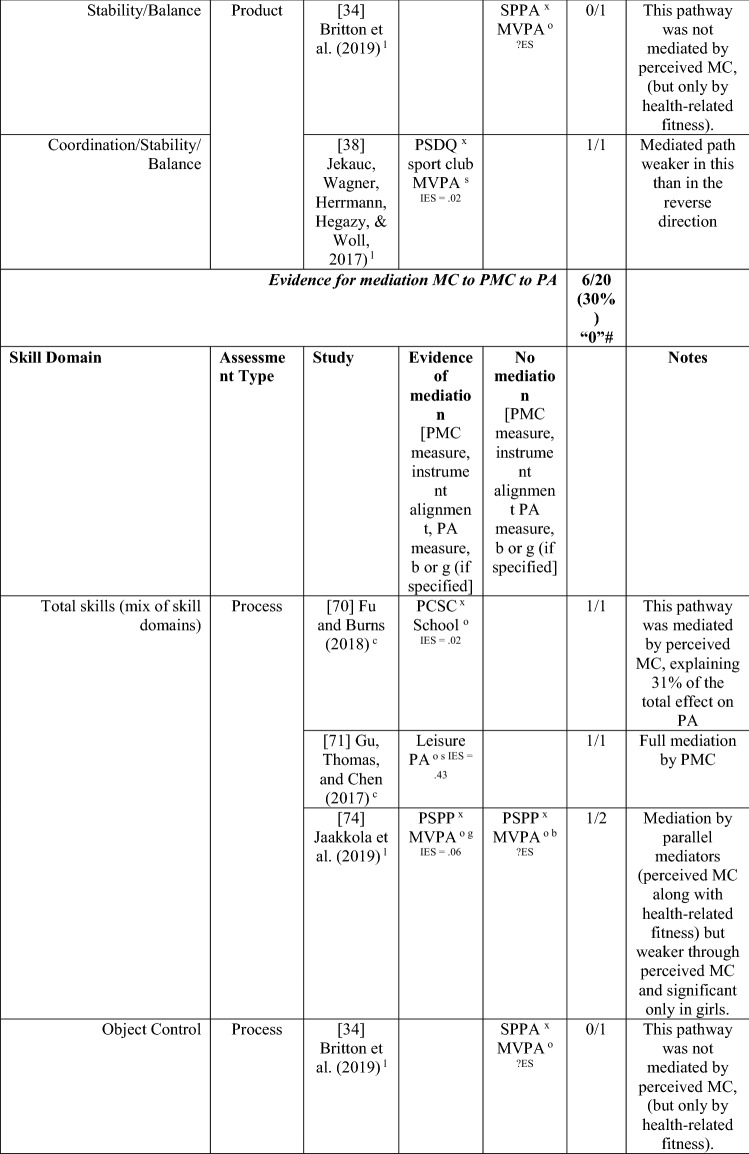

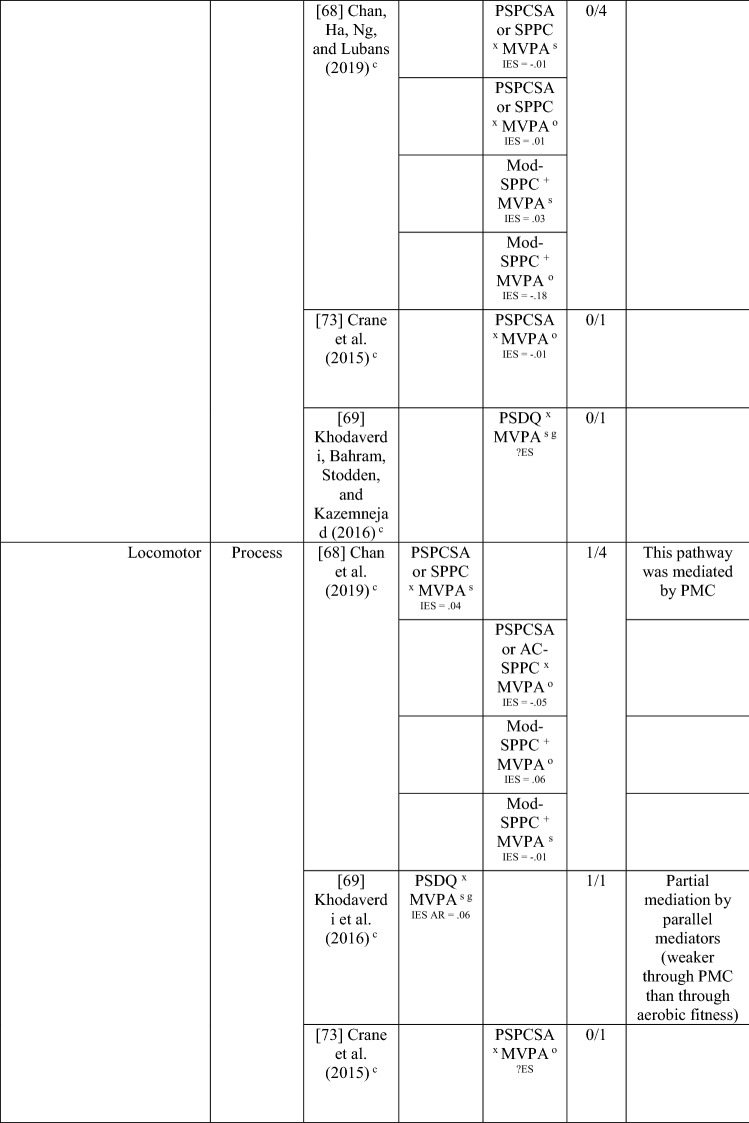

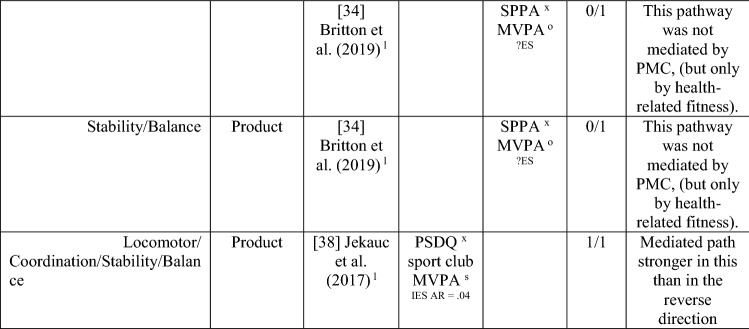
Perceived motor competence as a mediating variable between motor competence and physical activity (and the reverse)

If significant for whole sample, gender differences are not presented; adjusted values are used to report significance when they are reported; if only one skill is tested it is identified

*AR* author-reported, *C* cross-sectional, *IES* indirect effect size (no established guidelines for interpretation of IES for mediation, so actual values reported), *L* longitudinal, *Leisure PA* Self-Reported Leisure Time PA, *MC* motor competence, *Mod-SPPC* modified SPPC, *MVPA* moderate to vigorous PA, *OPA* objective PA, *PA* physical activity, *PCSC* Perceived Competence Scale for Children, *PMC* Perceived Motor Competence, *PSDQ* Physical activity subscale of the Self-Description Questionnaire, *PSPCSA* Pictorial Scale of Perceived Competence and Social Acceptance for Young Children, *PSPP* Physical Self-Perception Profile, *SPA* subjective PA, *SPPA* Self-Perception Profile for Adolescents, *SPPC* Self-Perception Profile for Children,  × non-aligned actual and perceived measurements, + aligned actual and perceived measurements, *?ES* unable to calculate effect size

^a^Based on the percentage of findings supporting the association, the variable was classified as either no association (0–33%), written as ‘0’; indeterminate/inconsistent (34–59%), written as ‘?’; or a positive ‘+’ or negative ‘−’ association (≥ 60%). When four or more studies found an association, it was classified as ‘++’ or ‘−−’ accordingly. If there were three or less studies in the domain, the strength of evidence was considered insufficient (I) to classify

Both longitudinal mediational studies [34, 38] and five of the seven studies using cross-sectional data [68–71, 74] reported at least one significant mediation for perceived MC. However, four of these studies also tested mediated pathways that were not significant [34, 68, 69, 74]. The longitudinal studies started in adolescence, and the cross-sectional studies that found mediation evidence were all in children aged 9–11 years.

Of the five studies that considered the path from PA to perceived MC to MC [34, 38, 72–74], five of nine analyses reported some evidence of mediation, classified as indeterminate (56% [‘?’]). Jekauc et al. [38] reported longitudinally that physical self-concept (unaligned with actual MC) was a mediator between sport-related PA (self-reported MVPA in sports clubs) and MC (product assessment). In 11-year-old American children, Burns and Fu [72] reported perceived MC (unaligned) as a mediator between school-day PA (assessed by pedometer steps) and MC (process assessment). Jaakkola et al. [74] reported that perceived competence was a mediator for both boys and girls using objectively measured PA and a product assessment of MC. Britton et al. [34] reported mixed findings in 12-year-old Irish children, using a non-aligned measure of perception, with mediation evidence linking MVPA to locomotor (process assessed) but not balance (product assessed) or object control skills (process assessed). A cross-sectional study in Canadian 5-year-olds reported no mediation evidence when assessing PA objectively and MC using a process assessment and a non-aligned measure of perceived MC [73].

Of the eight studies that considered the path from MC to perceived MC to PA, six [38, 68–71, 74] found evidence of mediation (some with multiple measures) with perceived MC, with an overall 6/20 analyses significant, categorised as no evidence (30% [‘0’]). Jekauc et al. [38] reported longitudinally (with German adolescents aged 14 and then 20 years) that physical self-concept (not aligned) mediated the relationship between MC (product assessment) and sport-related PA (self-reported). In American children (aged 10 or 11 years), two studies reported that perceived MC mediated the relationship between MC (process assessments) and pedometer steps measured during school-day PA [70] or during physical education combined with self-report of leisure activities [71]. In similarly aged Finnish children, Jaakkola et al. [74] reported that perceived competence mediated the pathway from MC (product assessment) to PA (using accelerometry) but only in girls. In some studies, different effects were reported according to the actual or perceived competence type and the way PA was measured. In 9-year-old Chinese children, Chan et al. [68] reported that perceived physical competence, using a non-aligned measure, mediated the relationship between locomotor skills (process assessment) but only for self-reported PA and not accelerometer-assessed MVPA. Chan et al. [68] also used an aligned measure of perception and found that it was not a mediator for either locomotor or object control skills. In a similar age group, Khodaverdi et al. [69] reported that perceived locomotor (but not perceived object control) skills mediated the relationship between MC (process assessment) and MVPA (self-reported) in Iranian girls. Two studies that assessed MC with a process assessment and MVPA objectively, reported no evidence of mediation for this direction. One Irish longitudinal study in which children were aged 12 years at baseline reported that a non-aligned measure (perceived athletic competence) did not mediate between MC (object control, locomotor or balance) and MVPA [34].

Studies that found a mediating pathway used a mixture of process [34, 68–72] and product [34, 38, 74] MC assessments. Most studies did not distinguish between types of MC. Still, those that used separate types of MC found evidence for locomotor skills (in self-reported PA) rather than object control skills [68, 69], although Britton et al. [34] found no evidence for mediation for either skill domain (with objectively assessed PA). Most studies did not use aligned measures of perceived and actual MC, except for Chan et al. [68] and Gu et al. [71]. While Chan et al. [68] and Gu et al. [71] both reported mediating pathways from MC to perceived MC to PA, Chan et al. [68] reported the mediating pathway for the non-aligned measure of perception. Overall, there is indeterminate evidence, according to the criteria [32], for the pathway from PA to perceived MC to MC (five of nine associations from five studies; 56% [‘?’]) and no evidence for the pathway from MC to perceived MC to PA (6 of 20 associations [30%], from eight studies reporting mediating associations). Nevertheless, it appears this pathway (in either direction) is more evident in older children, as all studies that found a mediating effect were in children aged ≥ 9 years.

### The Pathway from Health-Related Fitness to MC or the Reverse

#### Health-Related Fitness to MC

Six studies investigated the path from health-related fitness to MC [33, 36, 40, 42, 62, 64], with 27/101 of these analyses (27%, thus rated as no association [‘0’]) showing an association in the hypothesised direction between an aspect of fitness and an MC domain. When the study by Antunes et al. [42] was removed because it included more than eight comparisons within one study, the proportion of significant analyses was 59% (17/29), rated as indeterminate (‘?’). For most analyses, an effect size could not be calculated. For the study with multiple comparisons, the few significant analyses had large effects. See Table 11.

**Table 11:**
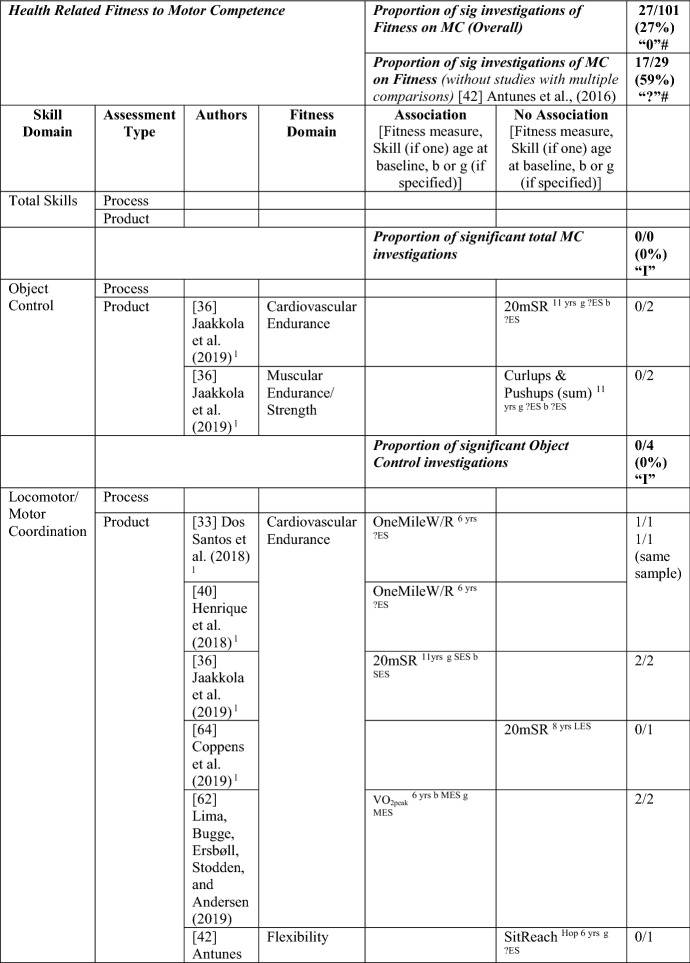

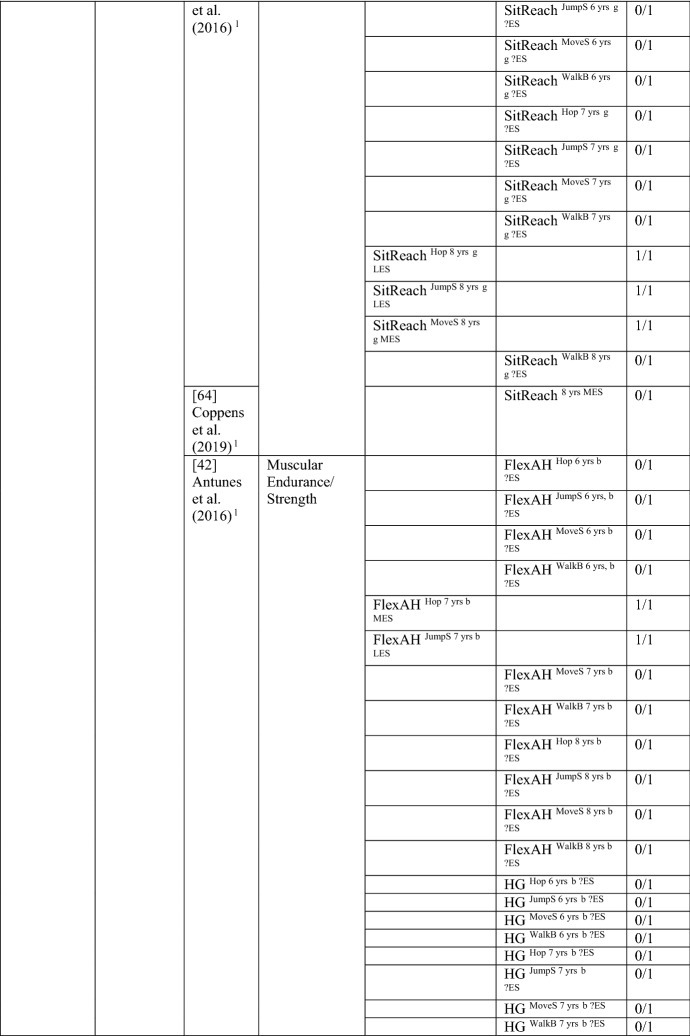

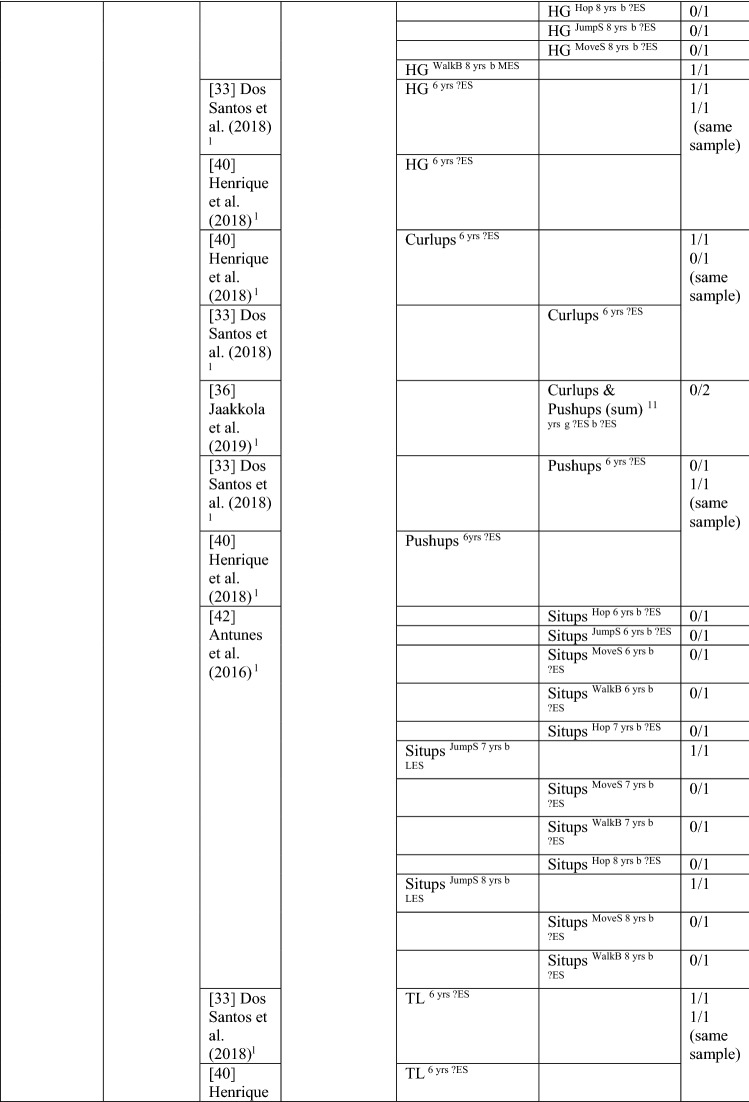

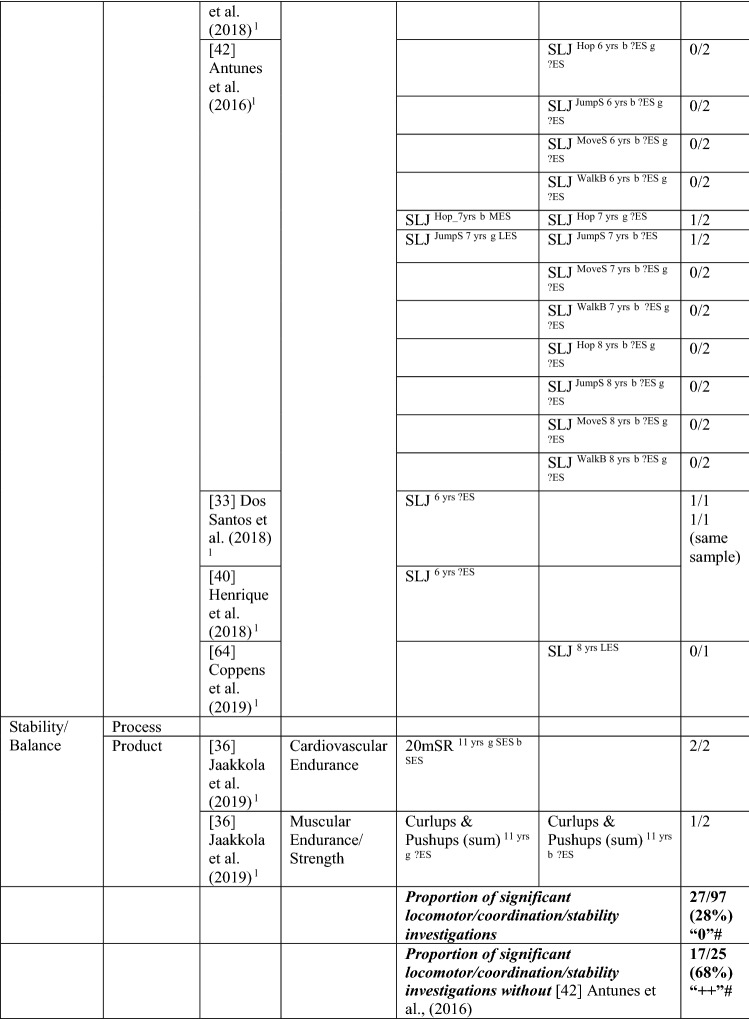
Summary of studies according to the pathway health-related fitness to motor competence

If significant for whole sample, gender differences are not presented; adjusted values are used to report significance when they are reported; if only one skill is tested it is identified

*20mSR* 20-m shuttle run, *FlexAH* flexed arm hang, *HG* handgrip, *JumpS* jump to the side, *L* longitudinal, *LES* large effect size, *MES* medium effect size, *MoveS* move sideways, *OneMileW/R* 1-mile walk/run, *Physical FT* physical fitness test, *SES* small effect size, *SitReach* sit and reach, *SLJ* standing long jump, *TL* trunk lift, *VO*_*2peak*_ peak oxygen uptake (continuous running on treadmill), *WalkB* walk backwards, *?ES* effect size could not be calculated due to lack of information

^a^Based on the percentage of findings supporting the association, the variable was classified as either no association (0–33%), written as ‘0’; indeterminate/inconsistent (34–59%), written as ‘?’; or a positive ‘+’ or negative ‘−’ association (≥ 60%). When four or more studies found an association, it was classified as ‘++’ or ‘−−’ accordingly. If there were three or less studies in the domain the strength of evidence was considered insufficient (I) to classify

All six studies used a product assessment of MC, with five studies using the body coordination test for children (Korperkoordinationtest fur Kinder [KTK]) [33, 40, 42, 62, 64]. Two of the six studies also investigated the reverse pathway [36, 62]. No study investigated fitness as a predictor of total MC. One study [36] investigated fitness (cardiovascular endurance and muscular endurance) as a predictor of object control skills, with none of the four analyses significant.

Cardiovascular endurance was investigated in five studies, with four studies reporting it was a predictor for motor coordination. Cardiovascular endurance (1-mile run/walk) at 6 years old predicted motor coordination across 3 years in the same sample of Portuguese children [33, 40] and in Danish children (this relationship changed over time for boys and was stable for girls) [62]. In Finnish children aged 11 years, cardiorespiratory endurance was a predictor of motor coordination 1 year later [36]. In contrast, Coppens et al. [64] reported that cardiorespiratory endurance at 8 years old did not predict motor coordination 2 years later in Belgian children. Jaakkola et al. [36] also reported that cardiorespiratory endurance predicted stability competence for girls and boys.

Flexibility was assessed using the ‘sit and reach’ test in two studies. Flexibility at 8 years old predicted motor coordination for Portuguese girls for hopping, jumping sideways and moving sideways [42]. Yet, flexibility did not predict coordination for 6- or 7-year-old girls in the same three skill tests or in walking backwards. In 8-year-old Belgian children, flexibility did not predict motor coordination 2 years later [64].

Five studies addressed muscular strength and endurance as a predictor of MC [33, 36, 40, 42, 64]. Antunes et al. [42] reported that, for boys, the flexed arm hang at 7 years old predicted hopping and jumping sideways but not moving sideways or walking backwards. Antunes et al. [42] also reported that the flexed arm hang for boys at 6 and 8 years old did not predict any of the assessed skills (hopping, jumping sideways, moving sideways or walking backwards).

Handgrip was reported as a predictor of motor coordination by three Portuguese studies: children aged 6 years followed up over 3 years (same child sample) [33, 40] and 8-year-old boys followed up 6 years later (walking backwards) [42]. However, handgrip was not a predictor for hopping, jumping sideways or moving sideways for 6-, 7- or 8-year-old boys or for walking backwards in 6- and 7-year-old boys in the same study [42].

One study reported that curl-ups at 6 years old did not predict motor coordination across 3 years in Portuguese children [33]. When this same sample was analysed with regard to consistently higher or lower skill performers across the 3 years, this was significant [40]. Curl-ups and push-ups at 6 years old also did not predict motor coordination across 3 years in one investigation of Portuguese children [33]. In contrast, push-ups were a predictor in the same sample using a different analysis [40]. Jaakkola et al. [36] reported that muscular fitness (composite of curl-ups and push-ups) at 11 years old was not a predictor of coordination for either sex 1 year later; however, muscular fitness did predict stability skills for girls [36]. Antunes et al. [42] reported that sit-ups in 7- and 8-year-olds but not in 6-year-olds predicted jumping sideways for boys. Also, sit-ups did not predict hopping, moving sideways or walking backwards in 6-, 7- or 8-year-old boys.

Lower body muscular strength and power (standing long jump) predicted motor coordination in two Portuguese samples [33, 40, 42]. For 6-year-old children, standing long jump predicted coordination across 3 years [33]. For 7-year-old boys, standing long jump predicted hopping at 12 years [42]. However Antunes et al. [42] also reported that the standing long jump at 6 and 8 years old did not predict hopping. Also, the standing long jump at 6, 7 and 8 years old did not predict jumping sideways, moving sideways or walking backwards. Furthermore, Coppens et al. [64] reported that standing long jump at 8 years old did not predict coordination 2 years later in Belgian children.

In summary, overall, there was indeterminate evidence for the pathway from fitness to MC (without the study with multiple comparisons). There was insufficient evidence for the pathway from fitness to total MC and to object control skills. There was no evidence for the pathway from fitness to locomotor/coordination/stability skills (27/97 [28%]); however, when the study with multiple comparisons was removed, there was strong positive evidence (‘++’) (17/25 [68%]).

#### MC to Health-Related Fitness

Five longitudinal studies [36, 51, 52, 62, 75] and one interventional study [59] investigated the pathway from MC to fitness. The proportion of significant analyses of MC on fitness was (23/37 [62%], thus rated strong positive [‘++’]). Similar to the other pathway direction, most effect sizes could not be calculated. Most studies addressed fitness components rather than a composite measure. To capture any patterns of association, the many fitness tests were grouped under health-related fitness domains (i.e., cardiovascular endurance, flexibility, muscular endurance/strength). Five studies used product MC assessments, with the KTK used as a measure of motor coordination in four of these studies [51, 52, 62, 75]. The intervention study in 8-year-old children by Cohen et al. [59] utilised a process assessment. This intervention was the only study that investigated total MC, reporting that total skills mediated the effect of the intervention on endurance fitness [59].

Two of the five studies investigated object control skills as a predictor of cardiorespiratory endurance [36, 59] and muscular endurance [36], with no significance. Concerning locomotor/motor coordination as a predictor of fitness, only one study used a composite measure of fitness, reporting that coordination at the age of 5–6 years predicted fitness 6 and 9 years later in Norwegian children [75]. Five studies [36, 51, 52, 59, 62], four of which used product assessments [36, 51, 52, 62] investigated MC as a predictor of cardiovascular endurance with fairly consistent associations. Motor coordination at age 6 years was a predictor for the 1-mile walk/run test 4 years later in another Portuguese sample, for boys (but not girls) in the upper levels of fitness compared with the lower levels [52]. In the study by Fransen et al. [51], children (aged 6 and 8 years at baseline) with higher MC generally had better cardiovascular endurance than children with low MC, and these differences remained constant over time. Lima et al. [62] reported longitudinal associations in boys and girls aged ≥ 6 years. Cohen et al. [59] reported that locomotor skills (process assessment) mediated the effect of the intervention on endurance fitness. Yet, in Finnish 11-year-old children, coordination and stability were not significant predictors of cardiovascular endurance for boys or girls 1 year later [36].

Only one study investigated flexibility. Fransen et al. [51] reported that Belgian children with higher motor coordination at 6 and 8 years old were more flexible over a 2-year period than those with lower coordination. Two studies investigated multiple aspects of muscular endurance and strength, with 6- and 8-year-old Belgian children with higher MC remaining better than children with less MC in the handgrip, push-ups, sit-ups and standing long jump over 2 years [51]. In Finnish 11-year-olds, Jaakkola et al. [36] reported that coordination predicted a composite muscular fitness score (curl-ups and push-ups) 1 year later in both boys and girls. Yet, in this same Finnish study, stability was a predictor of muscular endurance for girls only. See Table 12.

**Table 12:**
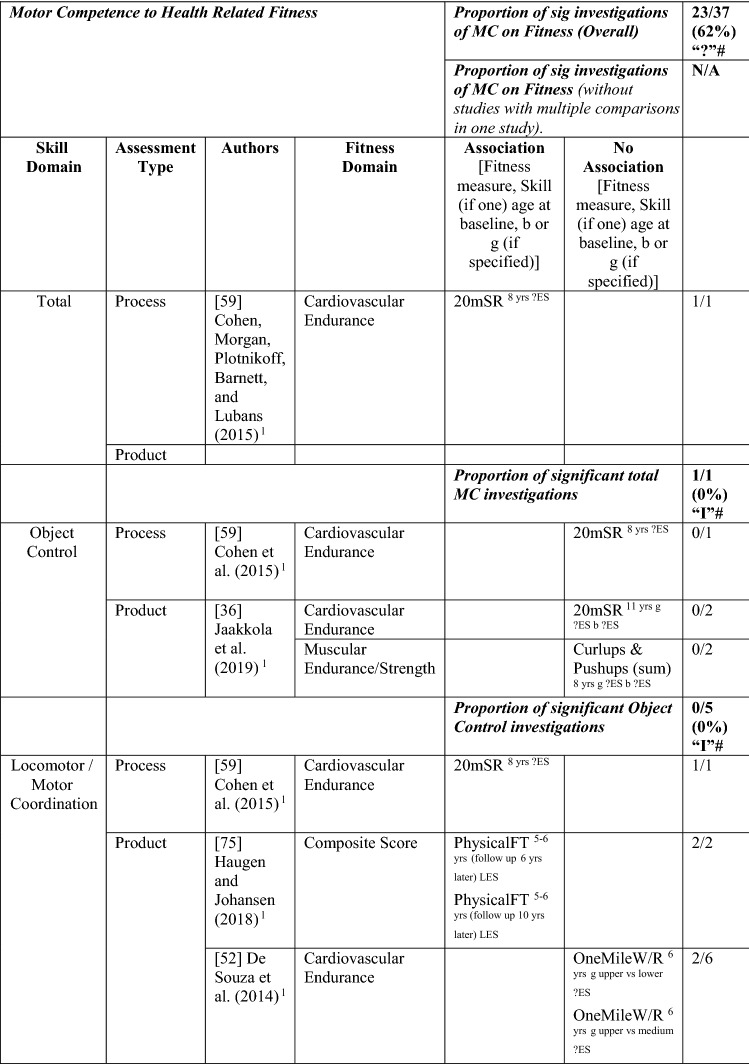

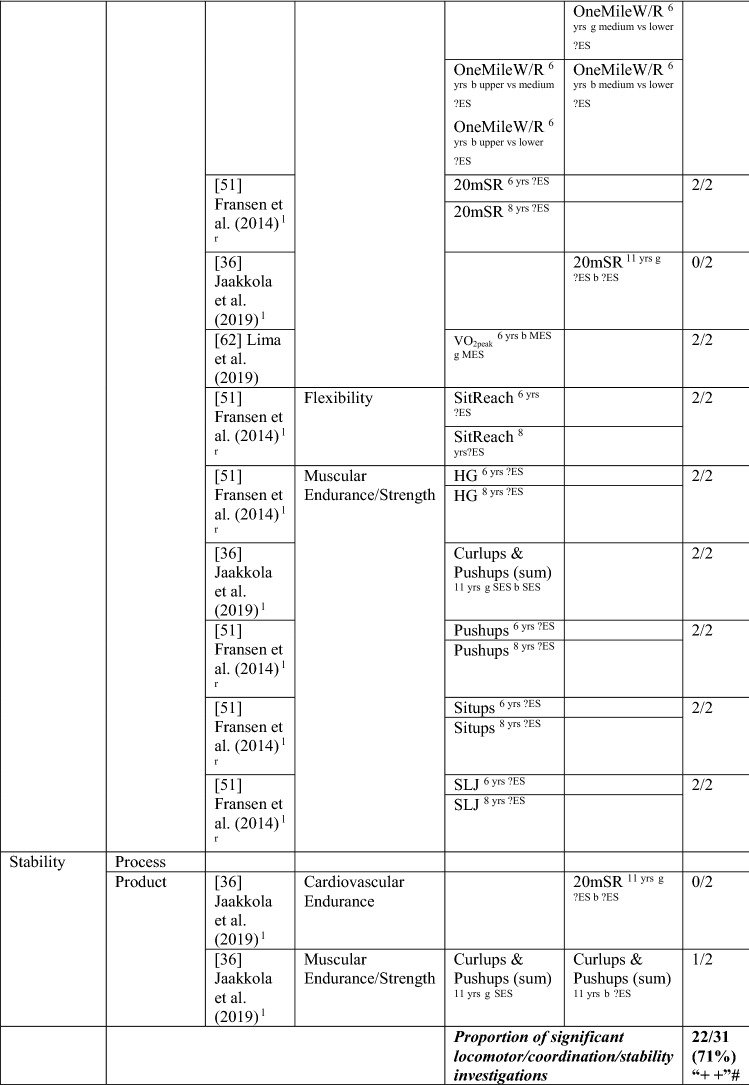
Summary of studies according to the pathway motor competence to health-related fitness

If significant for whole sample, gender differences are not presented; adjusted values are used to report significance when they are reported; if only one skill is tested it is identified

*20mSR* 20-m shuttle run, *HG* handgrip, *L* longitudinal, *LES* large effect size, *MC* motor competence*, MES* medium effect size, *OneMileW*/R one mile walk/run, *PhysicalFT* physical fitness test, *SES* small effect size, *SitReach* sit and reach, *SLJ* standing long jump, *VO*_*2peak*_ peak oxygen uptake (continuous running on treadmill), ?ES effect size could not be calculated because of lack of information

^a^Based on the percentage of findings supporting the association, the variable was classified as either no association (0–33%), written as ‘0’; indeterminate/inconsistent (34–59%), written as ‘?’; or a positive ‘ + ’ or negative ‘−’ association (≥ 60%). When four or more studies found an association, it was classified as ‘++’ or ‘−−’ accordingly. If there were three or less studies in the domain the strength of evidence was considered insufficient (I) to classify

In summary, overall, there was strong positive evidence for the pathway from MC to fitness but insufficient evidence for the pathway from total MC to fitness and from object control skills to fitness. There was strong positive evidence (‘++’) (22/31 [71%]) for the pathway from locomotor/coordination/stability skills to fitness.

#### Health-Related Fitness as a Mediator between MC and PA and the Reverse

Four studies (with seven analyses) investigated mediation from PA to fitness to MC. This was classified as strong positive (‘++’) evidence. Britton et al. [34], Jaakkola et al. [74] and Lima et al. [37] also investigated the reverse mediated pathway. Britton et al. [34] reported mediation evidence of a composite fitness measure via MVPA to object control, locomotor and stability skills 1 year later and that the pathway association was stronger in this direction. Lima et al. [37] reported that endurance fully mediated the relationship between objectively measured MVPA and a product assessment of MC and partially mediated the relationship between VPA and MC in a longitudinal study. Jaakkola et al. [74] reported that fitness was a mediator between objectively measured MVPA and a product assessment of total skills, partially mediated for Finnish boys and fully mediated for girls. Another cross-sectional study investigated this pathway direction in 12-year-old US children, reporting that cardiovascular endurance fully mediated between total skills and PA objectively measured [72]. See Table 13.

**Table 13:**
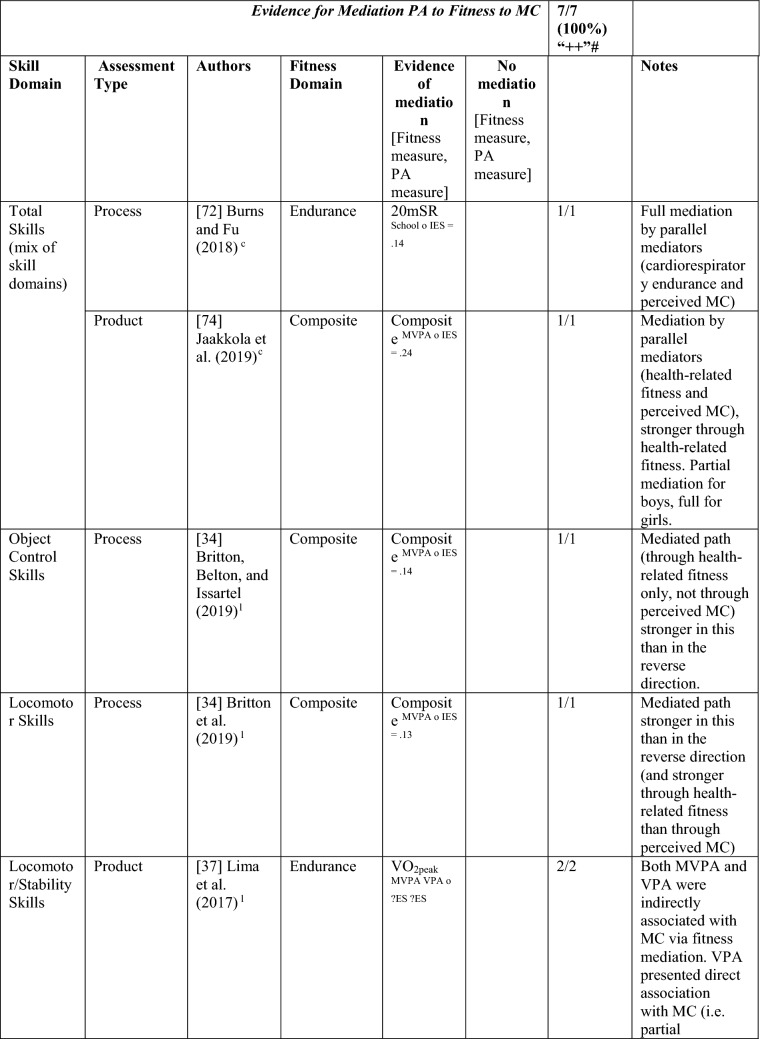

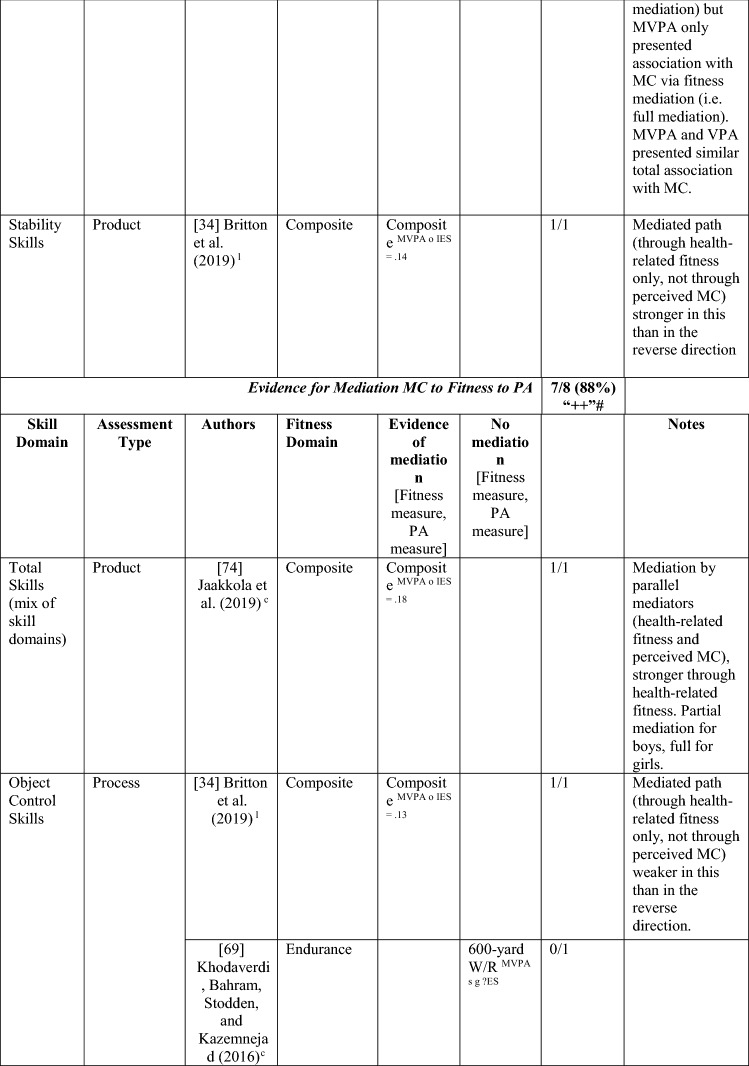

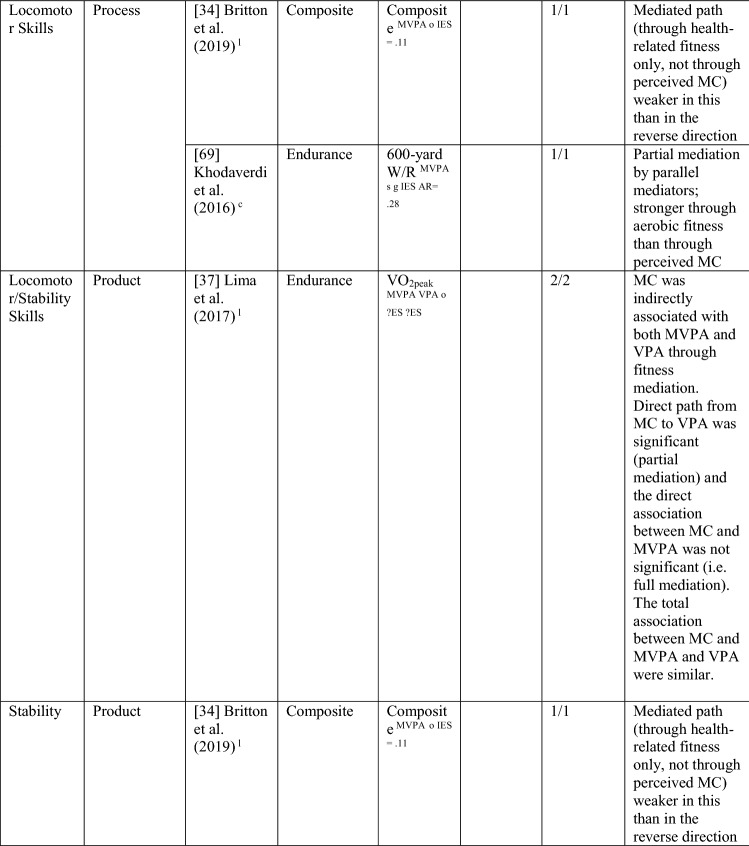
Fitness as a mediating variable between motor competence and physical activity (and the reverse)

*20mSR* 20-m shuttle run, *600 yard W/R* 600-yard walk/run test, *AR* author-reported, *CompositeFT* composite fitness test, *C* cross sectional, *IES* indirect effect size (no established guidelines for interpretation of IES for mediation, so actual values reported), *L* longitudinal, *MC* motor competence, *MVPA* moderate to vigorous PA, *OPA* objective PA, *PA* physical activity, *PMC* perceived motor competence, *SPA* subjective PA, *VO*_*2peak*_ peak oxygen uptake, *VPA* vigorous PA, *?ES* unable to calculate effect size

^a^Based on the percentage of findings supporting the association, the variable was classified as either no association (0–33%), written as ‘0’; indeterminate/inconsistent (34–59%), written as ‘?’; or a positive ‘ + ’ or negative ‘ − ’ association (≥ 60%). When four or more studies found an association, it was classified as ‘++’ or ‘−−’ accordingly. If there were three or less studies in the domain, the strength of evidence was considered insufficient (I) to classify

Two longitudinal studies [34, 37] and two cross-sectional studies [69, 74] (with eight different analyses, seven significant) investigated the mediated path from MC to fitness to PA. This was also classified as strong positive (‘++’) evidence. In 12-year-old Irish children, Britton et al. [34] reported evidence of mediation for object control, locomotor and stability skills using a composite fitness measure assessed at time point one (comprising tests such as the 20-m shuttle run, horizontal and vertical jump, push-ups, curl-ups) and an objective measure of MVPA 1 year later. Lima et al. [37] reported that endurance fully mediated the relationship between MC and objectively measured MVPA and partially mediated the relationship between MC and VPA. In a cross-sectional sample of 8-year-old Iranian children, Khodaverdi et al. [69] reported that the 600-yard running/walking test was a mediator between locomotor skills and subjectively measured MVPA but that object control skills were not. In 11-year-old Finnish children, a composite measure of health-related fitness mediated a product assessment of MC to MVPA (objectively measured) for both boys and girls [74]. The relationship showed a partial mediation for boys and a full mediation for girls [74]. In short, there was strong evidence for mediation between PA to MC via health-related fitness.

### The Level of Evidence for Each Pathway

**Fig. 3:**
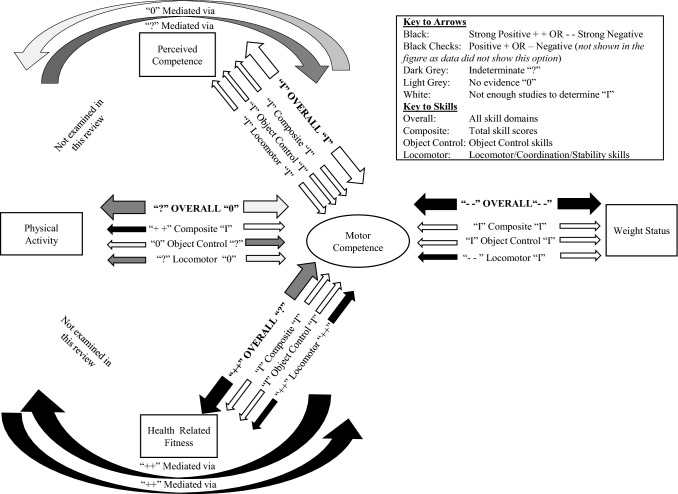
The level of evidence for each pathway summarised for each skill domain based on the findings of the current review

The level of evidence for each pathway is summarised for each skill domain according to the information provided above and represented in Fig. 3. The direct pathways between MC and weight status and the mediated pathway from MC to PA via fitness and the reverse are well supported. There was strong positive evidence for the path from MC to health-related fitness and indeterminate evidence for the path from fitness to MC. The path from PA to MC has mixed evidence depending on the skill domain and the amount of evidence available, and there were simply not enough studies to provide a level of evidence for the MC to perceived MC pathway, although there was indeterminate evidence for the mediating aspects of perceived MC from PA to MC.

## Discussion

The unique contribution of this review is that it examined all pathways of the Stodden et al. [2] model that relate to MC in conjunction with the prioritisation of mediation, longitudinal and experimental evidence from the past 5 years. Whilst other systematic reviews have examined aspects of the Stodden et al. [2] model, to our knowledge, none have examined each pathway related to MC and the mediated aspects in one review. It is clear from examining Fig. 3 that much more work is needed to support the premises of the model. We discuss each pathway in turn, followed by the mediated pathways, experimental evidence, studies that addressed more than one pathway and, finally, the strengths and limitations of the review and conclusions.

### Physical Activity

The bulk of studies (*n* = 28) in this review investigated the path to or from PA. The studies varied widely in terms of measure of PA (i.e., objective vs. subjective) and MC (i.e., product vs. process assessments), age and sex of the study sample and the length of follow-up, making it challenging to compare study findings. Overall, we found no evidence for the pathway from PA to MC. This result was unexpected and contradicts past reviews, which highlighted the association between MC and PA [5, 16, 25–27], but we believe this finding is valid because we carefully considered every analysis in each study, both the null and the significant associations, and we were able to provide a balanced picture. The bias in the literature towards publication of significant results in sports science is documented [76], and, as a result, researchers tend to report and highlight their significant associations. Subsequent researchers then cite these positive results, neglecting to mention the other analyses in the paper that were not significant, and this gives an impression of a weight of evidence or ‘truth’ that might not actually be present [76].

One previous review also reported inconsistent evidence for the MC and PA pathway, particularly when examining MC domains such as object control or locomotor skills [24]. The current review also had mixed results at the domain level, with inconclusive evidence for the path from PA to total skills, no evidence for locomotor, coordination and stability skills, and indeterminate evidence for object control skills. For the reverse pathway, MC to PA, the evidence was indeterminate. This highlights the importance of considering skill domains when synthesising results.

Another factor that distinguishes the current review from others and that may help to explain our results is the explicit focus on longitudinal studies. Since the search for the current review, a systematic review was published that included five longitudinal studies examining the association between MC and PA in the early years [20]. Four studies were also included in the current review [39, 55, 57, 77], and one was published before our inclusion date and thus not included [77]. Whilst these four studies provide some support for the longitudinal relationship between PA and MC, Bürgi et al. [77] reported in Swiss children that changes in balance from baseline to follow-up did not significantly predict objectively measured PA 9 months later. Also, even though Schmutz et al. [57] identified MC as a determinant of objectively measured PA 12 months later, MC at baseline was not significantly associated with change in either total PA or MVPA from baseline to follow-up. In contrast, a recent study in Canadian children aged 3–5 years reported that MC was a positive predictor of musculoskeletal fitness and vigorous PA over time and that better MC was associated with steeper increases in PA across time [78]. Yet in this study, there were no cross-sectional associations between objectively measured vigorous PA and MC when children were 3 years old. The study by Barnett et al. [39] in early childhood, included in the current review, also reported predictive associations (from age 3.5 to 5 years) but no cross-sectional associations (at age 5). Both these studies illustrated that the bias in the literature towards cross-sectional evidence could be cause for caution and that we need robust longitudinal evidence to illustrate how these variables interact over time. However, longitudinal studies need to be carefully designed so that robust inferences can be made. In the current review, very few studies provided a sample size justification, power description or variance and effect estimates, and few studies measured and adjusted for key controlling variables. This means that there may have been significant relationships amongst the variables of interest, but the study was neither powered nor designed to be able to detect these associations.

Finally, measurement of both variables is also an important factor to consider. In this review, we documented the MC measurement approach within each skill domain, but it was not possible to find any pattern because of the lack of studies in certain domains. We also documented the PA approach, in terms of objective or subjective measurement. PA is typically operationalised as intensity and/or duration rather than by type and quality [76], and this was reflected in this review. As such, PA may not (depending on the context) be closely related to skilled movement. For example, the common placement of an accelerometer at the waist would not capture the intensity of discrete skilled movement (e.g., throwing) [79], which means the association between MC in this instance and PA will likely be low. Please see a recent book chapter by Barnett et al. [80] for a full discussion on measurement issues in this area. Future research needs to carefully consider the type of measurement in use for each construct and consider the ramifications carefully in any synthesis.

### Weight Status

More studies (*n* = 9) investigated the path from weight status to MC than the reverse (*n* = 5). There was strong evidence of a negative association for weight status to MC and the reverse pathway, so long as studies with many multiple comparisons [42, 48] were excluded from the totals. These associations occurred for MC assessed by process and product assessments. The review by Cattuzzo et al. [17], which mainly included cross-sectional studies, also reported strong support for an inverse relationship between weight status and MC (82% of studies). In the current review, there was also strong support for a negative association for the pathway from weight status to locomotor/coordination and balance/stability skills and some support for the reverse. In short, it seems that excess body weight will be an obstacle to the development of MC, especially in children who are obese at a young age. An child who is overweight at a young age rarely changes to a normal weight percentile as a function of growth [81]. This means that obesity during early childhood will negatively affect the development of MC throughout childhood. The reverse is harder to prove. Studies must have a sufficiently large number of years of observation to detect normative changes (percentile), and the sample should preferably consist of children with a healthy weight at baseline and also considerable within-group variability in MC.

Past reviews (with mainly cross-sectional evidence) have either provided evidence that an unhealthy weight status is associated with poorer motor coordination (but not object control skill) because of the need to propel the body through space [82] or have not been able to provide this information as MC was not examined by skill domain [17]. The current review provided no clear determination with regard to object control skills and weight status because the number of studies was insufficient. This lack of evidence for the reverse path did not allow us to address the chicken-and-egg problem but adds to the evidence of wide and likely interconnected negative influence of overweight on PA behaviours and MC. In short, future researchers may wish to explore the relationship between MC and weight status further as this pathway direction was underexplored.

Most studies in the current review assessed BMI [40, 41, 48, 53, 60, 61, 63, 64] and body mass [33, 42]. Whilst some studies used more robust assessments of body composition (i.e., body fat percentage [48] and sum of skinfolds [40, 42, 62, 65]), there was no particular pattern of results in terms of the type of weight status assessment. In the review by Cattuzzo et al. [17], only 4 of 33 studies used robust assessment (skinfolds or bioelectrical impedance), whereas, in the current review, 5 of 11 studies did. This may indicate a move towards researchers utilising more robust assessments to measure weight status as recommended in the Robinson et al. [5] review.

### Fitness

There was strong positive evidence for a pathway from MC to fitness and indeterminate evidence for the reverse. In terms of domains, there were strong positive relationships in both directions for locomotor/coordination skills. There was not enough evidence in either pathway direction to associate object control skills with any aspect of health-related fitness. There were not enough studies to judge total skills as a predictor or outcome of fitness or for stability or flexibility as predictors or outcomes of MC. There was strong evidence that locomotor/coordination predicted muscular strength/endurance and some evidence for cardiovascular endurance as an outcome of MC. For the pathway from fitness to MC, there was strong evidence that cardiovascular endurance predicted locomotor/motor coordination. There was mixed evidence concerning MC and muscular strength/endurance.

These findings are confirmed by previous literature [5, 17, 18, 25]. An early review (from 2010) located four studies that all reported a positive relationship between MC and cardiorespiratory fitness [25]. The review by Cattuzzo et al. [17], which mainly included cross-sectional studies (82%), reported that all 16 studies that assessed cardiorespiratory fitness (either with a composite measure or independently) reported associations with MC. Cattuzzo et al. [17] also reported that 7 of 11 studies that assessed musculoskeletal fitness showed a positive association. However, Cattuzzo et al. [17] did not document whether non-significant results existed for each study in their summary tables. If we had only reported significant results per study, our overall picture would have been skewed towards the positive. However, a recent meta-analyses that did take into consideration null effects, reported similar moderate to large effects for the association between MC and cardiorespiratory and also musculoskeletal fitness [18]. The meta-analyses by Utesch et al. [18] highlighted the grey area between skills and fitness, where it can be hard to determine how to classify each. Executing fitness tests requires specific coordination patterns to be learned and executed with high inter- and intramuscular control, so such tasks encompass both MC and health-related fitness. As such, the notion of causation within the development of MC and health-related fitness is difficult to assess because both constructs are not independent [83]. MC and health-related fitness often share similar neuromuscular functioning with the underlying level of commonality between different types of fitness and motor skill tasks dependent on the tasks being compared [80]. From this point of view, the somewhat stronger evidence between fitness and locomotor versus fitness and object control (Fig. 3) can be explained by the assumption that locomotor MC tasks appeal to muscular strength/endurance (weight bearing) to a larger extent than do object control tasks.

### Perceived Motor Competence

Overall, evidence linking MC and perceived MC in either direction was insufficient and inconsistent. This was likely because of the small number of longitudinal/intervention studies conducted in the past 5 years. A recent meta-analysis regarding the association between actual and perceived MC reported significant small pooled effects for overall MC (*r* = 0.25), locomotor (*r* = 0.19), object control (*r* = 0.22) and stability/balance (*r* = 0.21) [21]. Nearly all (88.5%) of the 87 located studies in this meta-analyses were cross-sectional [21], so the field is in need of longitudinal studies.

A factor hypothesised to be potentially important in enhancing associations between actual and perceived MC is alignment between the measure used for perception of competence and the measure for actual MC (i.e., perception of catch vs. objective assessment of a catch) [4]. However, the results from the current review (albeit based on a small number of studies) suggested that more aligned measures do not moderate the association. Similarly, the review by De Meester et al. [21] did not find that the level of instrument alignment moderated the association between the perception measure and the actual measure of MC. However, De Meester et al. [21] compared studies with aligned measures and all studies without aligned measures without considering the degree of misalignment in these comparison studies. De Meester et al. [21] noted that this decision was because of the complexity of trying to ascribe levels of alignment between instruments and suggested future researchers could seek to try and further unravel the relationship between these variables. Consideration of other potential moderators (e.g., cognitive function, motivation towards PA) could also be investigated.

### Mediated Pathways

In terms of fitness, seven analyses from four studies reported mediation from PA to MC, and seven of eight analyses from four studies found evidence of the reverse direction, providing strong positive evidence. For perceived MC, the path from PA to MC was classified as indeterminate, and the reverse was classified as no evidence. Whilst several studies since 2014 have assessed mediation, few used longitudinal designs [34, 37, 38]. One recent cross-sectional study using pathway analyses extended the conceptual model by Stodden et al. [2] by examining actual MC, fitness and perceived MC and including a perception measure that aligned to fitness rather than just to MC [84]. Whilst this extended the perception part of the model to be more comprehensive, longitudinal evidence is needed. Mediating pathways should be tested using longitudinal designs, since analyses using cross-sectional data may inflate causal estimates [85]. Not using a longitudinal design can result in over-reporting of likely mediation, so the cross-sectional literature could include a bias towards perceived MC (or fitness) being considered a mediator between MC and PA. This review included cross-sectional meditated models, simply because there is not yet much evidence in this area. Future studies should investigate mediation using a longitudinal design to be able to answer the question of mediated pathways in the model [2].

### Experimental Evidence

Very few intervention studies met our inclusion criteria (i.e., studies needed to have been able to determine whether a change in MC resulted in a change in fitness, PA or perceived MC or vice versa). Whilst many interventions assessed both PA (and or fitness) and MC with the intention to change both, analyses and/or designs were mostly targeted to detect eventual parallel gains, so it was not possible to state whether change in MC explained change in the other variable (or vice versa). Only one intervention assessed whether change in MC affected change in PA and fitness, finding that total skills mediated the effect of the 12-month intervention on moderate to vigorous PA and fitness in Australian 8-year-olds [59]. The other interventions located in the current review did not manage to show that MC improvements related to PA in 12-year-old Irish youth [60] or improvements in perceived MC in 4-year-old Greek children [67] and 12-year-old Australian girls [66]. A previous review of intervention studies had a stated aim similar to ours, i.e., to “determine whether a relationship exists between change in fundamental movement skills and change in PA levels”, yet the meta-analyses included articles that examined each outcome separately [13]. Similarly, the review by Figueroa and An [16] included randomized controlled trials, but none of the interventions (e.g. Jones et al. [86]) actually tested whether a change in one variable related to a change in the other variable. In another systematic review, Tompsett et al. [15] reported that PA increased in 7 of 12 MC interventions, but all these interventions [87–91] except Cohen et al. [59] analysed MC and PA as separate outcomes. To test causality, researchers need to show that change in one variable results in change in the other variable. Future researchers should consider how to address this within their study design and analyses.

### Model in Entirety

No longitudinal studies included all the variables and pathways of the model conceptualised by Stodden et al. [2]. Thus, there is no evidence base to support the model as a whole, only piecemeal evidence that calls for more holistic research designs. However, seven studies [10, 33, 34, 37, 40, 42, 43, 65] are worth noting as they assessed multiple model aspects in relation to MC. The study by Reyes et al. [43] in Portuguese children, assessed children from six age cohorts (aged 5–9 years), annually over 3 years, reporting that BMI (negative) and fitness (positive) were associated with developmental trajectories of MC but not PA. Dos Santos et al. [33] followed children annually from the age of 6 to 9 years and also showed that fitness was associated with MC change but PA was not. These authors also reported that children with a more linear body size/shape had better MC over time [33]. Antunes et al. [42] examined children aged 6–8 years who were followed up 6 years later and reported a number of fitness and MC associations but only one association with PA. Lima et al. [37] reported evidence of longitudinal mediation in the relationship between MC and PA via fitness in Danish children. In another study, Lima et al. [65] investigated PA, MC and fitness in relation to body fatness across 7 years (at ages 6, 9 and 13 years). Mediated effects (of PA—objectively measured, MC and fitness) were also analysed but in relation to body fatness (rather than PA) so did not meet inclusion for this aspect in our mediation pathways. Nevertheless, it is worth noting that fitness mediated the associations between PA, MC and body fatness and that MC mediated the associations between PA and body fatness [65]. PA only indirectly influenced body fatness [65]; as such, all these studies collectively suggest that PA is less important than fitness in the model by Stodden et al. [2]. Britton et al. [34] examined relationships in both directions for MC and PA as well as the mediating roles of perceived athletic competence and health-related fitness in adolescents and followed up 1 year later. Health-related fitness was more important mediator than perceived athletic competence, further supporting the importance of fitness in the model. The constructs of fitness and MC are closely and synergistically related (as discussed in Sect. 4.3), which may help to explain why fitness appears to play a bigger part in the model pathways. Also as discussed, PA is typically operationalised as intensity and/or duration rather than by type and quality [76] and, as such, may not be closely associated with skilled movement.

### Strengths and Limitations

The current review considerably expands the evidence base by synthesising mediation, longitudinal and experimental studies within the last 5 years, including studies published in languages other than English. Further strengths include using PRISMA guidelines, clear inclusion and exclusion criteria, careful analysis and consideration of null results, calculation of effect sizes where possible and multiple authors double checking each other’s categorisation. The broad scope of this review meant that we could not assess how other relevant variables (e.g., diet, genetics, cultural settings, growth and maturation, cognition, motivation) related to the core variables in the model. For instance, an important consideration in understanding the influences on health-related fitness and MC is biological maturation and associated allometric growth, given the differential impact maturational status and growth has on MC and health-related fitness [43]. Further, it was not possible to synthesise information to understand the unique developmental underpinnings of the model by Stodden et al. [2] (i.e., whether the direction of the relationships changed over time as children developed). As such, any effects might wash each other out when studies span a large time span. Each study had different start, end, and follow-up points (as well as differently measured constructs within the variables) and there were simply not enough studies to categorise age groups and follow-up times in any meaningful way. Longitudinal research that tests the hypothesis of increasing strength of associations among variables as a function of development is needed.

Meta-analyses were not permitted because of the limited number of studies with homogenous measures within a variety of domains regarding MC, PA, fitness and weight status. Future researchers should carefully consider the design of, and the measures used in, a study. Applying standard and robust measures and reporting fully on these parameters will facilitate comparing and pooling data from different studies. Our results syntheses were conducted based on the number of significant associations versus the number of non-significant associations. As stated in the discussion on the PA pathway, this helped to provide a balanced picture of the literature. However, some studies included multiple analyses within one study, and this way of calculating results does not take into account the weighting of results from one single study. In an effort to combat this ‘bias,’ we decided to present the results with and without studies with multiple (more than eight) analyses in the one study. If it had been possible to perform meta-analyses, we could have considered the weighting of the data from different studies. Future reviews need to consider the weighting of evidence from single studies and ensure this is considered in results analyses and interpretations. What this current review has highlighted is that these single studies can shift the weight of evidence, which shows the importance of the process of result synthesis. We did calculate effect sizes, where possible, in an attempt to better understand the strengths of associations across pathways. This served to highlight the few studies in which sufficient information was reported to enable calculation of effect sizes. Various study aspects were not reported, which then precluded calculation of effect sizes, for instance, a lack of reporting regarding:

- sample size (e.g., for treatment and control groups and subgroups),
- subgroups (e.g., not providing F values for subgroups),
- means (e.g., reported overall for dependent variable, but not for subgroups),
- standard deviations (e.g., for the total sample for the means at different time points/moments or for subgroups),
- intervention group detail (e.g., the means and standard deviations of the dependent variable for baseline and treatment groups),
- all variables in a model (e.g., beta—both adjusted and non-adjusted, overall F values for multilevel modelling, significant and non-significant R2),
- non-significant values (e.g., commonly no details were reported for non-significant values such as non-significant correlations) and/or
- the regression coefficient in mediator studies (i.e., between the independent variable and the mediator variable and also between the mediator variable and the dependent variable).

Future analyses need to report effect sizes, or sufficient information for effect sizes to be calculated, especially when null results are reported.

## Conclusion

Overall, there was evidence of a strong negative association for a pathway from weight status to MC and the reverse. There was strong positive evidence for the path from MC to health-related fitness and indeterminate evidence from fitness to MC. There was strong evidence of a positive path from locomotor/coordination skills to fitness in both directions. There was indeterminate evidence for a pathway from MC to PA and no evidence for the reverse. There was insufficient evidence between MC and perceived MC. Conclusions on mediation outcomes are weakened by the predominantly cross-sectional nature of the available evidence and the limited studies, with indeterminate evidence for the PA to MC to perceived MC mediated pathway (and insufficient evidence for the reverse) but strong positive evidence for the fitness-mediating pathway. This review has gone “through the looking glass”, as described in *Alice in Wonderland* [92] when things are not as you thought them to be. Our findings do not provide the support for the MC to PA pathway that previous review literature suggested. Relying on many cross-sectional studies for evidence creates a bias, as the proximal measurement of variables is likely to contribute to more associations. Also, publication bias—highlighting significant results and overlooking the non-significant associations and not providing the effect size—has likely contributed to a picture of positive pathways that may not be accurate. To truly test the model authored by Stodden et al. [2], the field is in need of robust longitudinal studies across early childhood and into adolescence that include multiple variables from the model, have multiple time points and account for potential confounding factors.

## Supplementary Information

Below is the link to the electronic supplementary material.

Supplementary file1 (DOCX 18 kb)

Supplementary file2 (DOCX 63 kb)

Supplementary file3 (DOCX 37 kb)

Supplementary file4 (DOCX 22 kb)

Supplementary file5 (DOCX 37 kb)

Supplementary file6 (DOCX 44 kb)

Supplementary file7 (DOCX 33 kb)

Supplementary file8 (DOCX 32 kb)

## Declarations

### Availability of data and material

Data extracted from the articles are available in the supplementary tables. Data extracted for the effects sizes are available from the corresponding author on reasonable request.
