# Supplementary material for: Through the Looking Glass: A Systematic Review of Longitudinal Evidence, Providing New Insight for Motor Competence and Health
Source: Sports Med. 2021 Aug 31;52(4):875–920. doi: 10.1007/s40279-021-01516-8 (PMC8938405; doi:10.1007/s40279-021-01516-8)
Supplement: Supplementary file 1 — Supplementary file1 (DOCX 18 kb) [file 40279_2021_1516_MOESM1_ESM.docx]

Supplementary Table 1: Search terms for each construct of interest. Constructs linked by the ‘OR’ Boolean operator correspond to individual reviews.

| Motor Competence | “motor skill*” OR “movement skill*” OR “motor development” OR “gross motor” OR “motor performan*” OR “Motor proficien*” OR “motor abilit*” OR “object manipulation” OR “motor coordination” OR “actual competen*” OR “object control” OR “locomotor skill*” OR “motor proficiency” OR “motor competen*” OR “movement competenc*” OR “motor fitness” OR “fundamental movement” OR “fundamental motor” OR “basic movement” OR “manipulative skill*” OR “motor function*” OR “athletic skill*” OR “athletic competen*” OR “skill proficiency” OR “movement pattern” OR “motor fitness” |
| --- | --- |
| AND | |
| Children | “child*” OR “adolescen*” OR “student*” OR “teen*” OR “youth” OR “pediatric*” OR “paediatric*” OR “pube*” OR “juvenil*” OR “school*” OR “youngster*” OR “preschool*” OR “kindergart*” OR “kid” OR “kids” OR “playgroup*” OR “play-group*” OR “playschool*” OR “prepube*” OR “preadolescen*” OR “junior high” OR “high school*” OR “middle school*” OR “senior high” OR “young people*” OR “young person*” OR “minor*” OR “elementary school*” OR “primary school*” |
| AND | |
| Physical activity  (Review 1) | “physical* activ*” OR “physical inactiv*” OR “sedentary” OR “motor activit*” OR “physical education” OR “physical exercise” OR “exercise training” OR “sport” OR “active play” OR “walking” OR “active commut*” OR “energy expenditure” |
| OR | |
| Weight status  (Review 2) | “body composition” OR “waist circumference” OR “body weight” OR “weight” OR “body fat” OR “anthropometr*” OR “obes*” OR “overweight” OR “adipos*” OR “BMI” OR “skinfold” OR “DEXA” OR “BIA” ” OR “bioelectrical impedance analysis” OR “dual-energy x-ray absorptiometry” OR “body mass index” |
| OR | |
| Health-related fitness  (Review 3) | “fitness” OR “physical fitness” OR “musc* fitness” OR “musc* strength” OR “strength” OR “endurance” OR “cardiorespiratory fitness” OR “speed” OR “flexibility” OR “musc* power OR “muscular endurance” |
| OR | |
| Perceived motor competence  (Review 4) | “perceived motor competence” OR “perceived sport* competence” OR “perceived competence” OR “perceived physical competence” OR “perception* of competence” OR “perceived skill competence” OR “perceived skill proficiency” OR “perceived movement skill competence” OR “skill perception*” OR “perceived athletic competence” OR “perceived movement competence” OR “perceived athletic skill*” OR “perceived movement skill*” OR “perception of physical competence” OR “perceptions of physical competence” OR “perceived object“ OR “perceived locomotor” OR “self-belief” OR “self-concept” OR “self-esteem” OR “self-efficacy” OR “global self-worth” OR “self-perception*” OR “self-awareness” OR “self-rating*” OR “self-confidence” |
