# Supplementary material for: Through the Looking Glass: A Systematic Review of Longitudinal Evidence, Providing New Insight for Motor Competence and Health
Source: Sports Med. 2021 Aug 31;52(4):875–920. doi: 10.1007/s40279-021-01516-8 (PMC8938405; doi:10.1007/s40279-021-01516-8)
Supplement: Supplementary file 2 — Supplementary file2 (DOCX 63 kb) [file 40279_2021_1516_MOESM2_ESM.docx]

| **Supplementary Table 2. Motor Competence and Physical Activity Results** | | | | | | | | | | | | | | | |
| --- | --- | --- | --- | --- | --- | --- | --- | --- | --- | --- | --- | --- | --- | --- | --- |
|  | ***Longitudinal Studies*** | | | | | | | | | | | | | | |
| **Study** | **Country** | **Intervention Description** | **Time points # (Duration)** | **Sample #**  ***(M, F)*** | **Age (SD)** | **MC measure** | **MC scores at each timepoint**  ***Mean (SD)*** | | **PA measure**  ***Objective/Subjective***  **Duration of measurement** | | **PA scores at each timepoint**  ***Mean (SD)*** | **Analysis** | **Initial analyses (e.g. correlations)** | **Pathway tested and values** | **Overall findings (based on models rather than initial analyses)** |
| [42] Antunes et al. (2016) | Portugal | N/A | 2 (6 years) | 158 (83 M, 75 F) | T1:  Group 1: 6  Group 2: 7  Group 3: 8  T2:  Group 1: 12  Group 2: 13  Group 3: 14 | KTK  *Product*  TGMD-2  *Process* (assessed, but not in relation to PA) | **Walking backwards**  ***Males***  T1  Group 1: 40.0(11.3)  Group 2: 44.5(11.1)  Group 3: 49.1(12.1)  T2  Group 1: 59.9(12.1)  Group 2: 55.8(13.0)  Group 3: 64.2 (9.4)  ***Females***  T1  Group 1: 34.3(12.3)  Group 2: 44.0(10.4)  Group 3: 46.3(12.7)  T2  Group 1: 49.0(16.9)  Group 2: 56.9(9.6)  Group 3: 56.5(13.8)  **Hopping**  ***Males***  T1  Group 1: 21.4(10.2)  Group 2: 29.4(12.4)  Group 3: 35.2(7.7)  T2  Group 1: 61.1(14.4)  Group 2: 60.9(16.0)  Group 3: 70.0 (9.6)  ***Females***  T1  Group 1: 19.4(11.1)  Group 2: 29.3(8.8)  Group 3: 33.2(13.9)  T2  Group 1: 51.7(12.9)  Group 2: 54.8(10.6)  Group 3: 52.4(11.7)  **Jumping sideways**  ***Males***  T1  Group 1: 31.0(8.8)  Group 2: 34.3(7.8)  Group 3: 42.1(7.3)  T2  Group 1: 70.8(11.4)  Group 2: 68.7(15.0) Group 3: 78.1(7.3)    ***Females***  T1  Group 1: 31.3(7.2)  Group 2: 41.7(13.1)  Group 3: 42.6(12.2)  T2  Group 1: 64.2(14.6)  Group 2: 67.6(11.2)  Group 3: 69.7(11.6)  **Moving sideways**  ***Males***  T1  Group 1: 30.7(4.8)  Group 2: 34.1(4.7)  Group 3: 37.4(4.2)  T2  Group 1: 50.0 (6.4)  Group 2: 49.1 (7.5)  Group 3: 54.2 (5.2)  ***Females***  T1  Group 1: 28.5(5.5)  Group 2: 32.9(4.9)  Group 3: 34.5(5.5)  T2  Group 1: 45.7(10.5)  Group 2: 50.4 (7.1)  Group 3: 50.3 (6.7) | | Baecke questionnaire (Baecke et al., 1982)  *Subjective* Sport score (measure of participation in top two sports), sport index (sum of sport score parameter divided by four), leisure index (frequency of PA activities in leisure time). | | **Sport score**  ***Males***  T1 Group 1: 1.5(0.8)  Group 2: 1.5(0.6)  Group 3: 1.7(0.7)  T2  Group 1: 2.1(0.9)  Group 2: 1.7(0.8)  Group 3: 1.9(0.9)  ***Females***  *T1*  Group 1: 1.3(0.5)  Group 2: 1.4(0.7)  Group 3: 1.2(0.5)  T2  Group 1: 1.7(0.8)  Group 2: 1.6(0.6)  Group 3: 1.8(0.9)  **Sport index**  ***Males***  T1  Group 1: 2.8(0.8)  Group 2: 2.8(0.6)  Group 3: 2.9(0.4)  T2  Group 1: 3.1(0.7)  Group 2: 2.9(0.6)  Group 3: 3.0(0.6)  ***Females***  T1  Group 1: 2.4(0.5)  Group 2: 2.6(0.5) Group 3: 2.5(0.4)  T2  Group 1: 2.6(0.6)  Group 2: 2.5(0.6)  Group 3: 2.7(0.7)  **Leisure-time index**  ***Males***  T1  Group 1: 2.6(0.8)  Group 2: 2.5(0.6)  Group 3: 2.8(0.6)  T2  Group 1: 2.9(0.5)  Group 2: 2.7(0.5)  Group 3: 2.8(0.5)  ***Females***  T1  Group 1: 2.6(0.5) Group 2: 2.6(0.4)  Group 3: 2.7(0.7)  T2  Group 1: 2.4(0.6)  Group 2: 2.5(0.7)  Group 3: 2.5(0.5) | Repeated-measures multiple analysis of variance; Stepwise multiple linear regression |  | **PA (T1) 🡪MC (T2)**  ***Females***  **(Group 1; leisure time index and moving sideways only)**  β = 0.52, Partial R^2^ = 0.26  Leisure time index was not predictive of any skill in Group 2 or 3 for females, and not at all for males.  Sport score and sport index not predictive of any skill for males and females. | For girls only leisure time  physical activity at 6 years was a predictor of moving sideways at  12 years. |
| [39] Barnett, Salmon, and Hesketh (2016) | Australia | N/A | 2 (T2 to T3 = 18 months) | *T2:* 118 (53 M, 65 F)  *T3:* 127 (59 M, 68 F) | *T2:* 3.5 (0.2)  *T3:* 5.0 (0.1*)* | TGMD-2 (at T3 only)  *Process* | **Total Scores**  T3: 49.7 (9.7)  **Locomotor Scores**  T3: 26.0 (5.5)  **Object Control Scores**  T3: 23.3 (6.1) | | Accelerometer (ActiGraph GT1M)  *Objective,* 8-day wear time, total MVPA | | **Total MVPA**  T2: 42.5 (16.1)  T3: 52.8 (17.9) | Linear Mixed Models |  | **PA🡪MC**  **Total Skill**  PA(T2) 🡪 MC(T3): B = 0.11  **Object Control Skill**  PA (T2) 🡪 MC (T3) B = 0.03  **Locomotor Skill**  PA(T2) 🡪 MC(T3) B = 0.07* | MVPA at age 3.5 years was a significant predictor of locomotor skills  MVPA was not a predictor of total or object control skills |
| [34] Britton, Belton, and Issartel (2019) | Ireland | N/A | 2 (1 year) | 224 (110 M, 114 F) | 12.3 (0.0) | TGMD-3 (kick, catch, overhand throw, one- and two-hand strike, run, skip, horizontal jump) *Process*  Victoria Department of Education Training Manual (vertical jump)  *Process*  MABC-2 (two-board balance, zigzag hop, walking toe to heel backward)  *Product* | **Locomotor skills**  ***Males***  *T1:* 29.6 (3.5)  *T2:* 31.1 (2.8)  ***Females*** *T1:* 29.2 (3.6)  *T2:* 29.5 (3.3)  **Object control skills**    ***Males***  *T1:* 33.2 (4.2)  *T2:* 36.4 (2.3)    ***Females*** *T1:* 26.1 (7.0)  *T2:* 30.5 (4.6)  **Balance/Stability**  ***Males***  *T1:* 36.6 (8.1)  *T2:* 38.6 (6.7)    ***Females***  *T1:* 36.4 (8.7)  *T2:* 39.2 (6.9) | | Accelerometer (ActiGraph GT1M, GT3X, GT3X+, wGT3X-BT); MVPA mins  *Objective,* 7 days during waking hours | **MVPA Minutes**  ***Males***  *T1:* 54.4 (26.3)  *T2:* 44.2 (19.2)    ***Females***  *T1:* 46.3 (16.0)  *T2:* 37.1 (11.7) | | SEM |  | **PA (T1)🡪MC (T2)**  **Object Control**  β = 0.35**  No other significant pathways from PA to locomotor or balance/stability  **MC (T1)🡪PA (T2)**  No significant pathways from any MC variable to PA | Reciprocal relationships were stronger in the direction of MVPA predicting later MC, compared to the reverse direction. But this only appears to be substantial for object control skills. |
| [48] Bryant, James, Birch, and Duncan (2014) | United Kingdom | N/A | 2 (1 year) | 281 (129 M, 152 F) | T1: 8.9 (1.4)  T2: 9.8 (1.4) | Process checklist from New South Wales “*Move it Groove it*” (Sprint Run, Side Gallop, Hop, Kick, Catch, Overarm Throw, Vertical Jump and Static Balance)  *Process*  Objective Measurements (sprint run, vertical jump)  Product | | NR | Pedometer (New Lifestyles, NL2000)  *Objective,* 4 days (2 weekday, 2 weekend; average daily step, average weekend step, and average weekday step count | **Daily Steps**  ***Males***  T2: 9712 (3816)  ***Females***  T2: 8064 (3488)  ***Combined***  T2: 8820 (3724)  **Weekend Steps**  ***Males***  T2: 8819 (4427)  ***Females***  T2: 7314 (4116)  ***Combined***  T2: 8005 (4316)  **Weekday Steps**  ***Males***  T2: 10,514 (4236)  ***Females***  T2: 8830 (3741)  ***Combined***  T2: 9603 (4053) | | Linear Regression |  | **MC 🡪PA**  ***Males***  **Catch (T1) 🡪 daily steps (T2)**  β Slope  *= 3623*, R^2^ =6.7%*  Sprint run, side gallop, hop, kick, overarm throw, vertical jump, and balance were not significant  ***Females***  **Hop (T1) 🡪 Daily Steps (T2)**  β Slope = 3178***, *R^2^ =24.1%*  **Hop (T1) 🡪 weekday steps (T2)**  β Slope = 3890***, *R^2^*  *= 14.6%*  ***Jump height (T1) 🡪 daily steps (T2)***  β Slope = 3452***, *R^2^ =16.3%*  **Jump height (T1) 🡪 weekend steps (T2)**  β Slope = 4309***, *R^2^ =13.5%*  **Jump height (T1) 🡪 weekdaysteps (T2)**  β Slope = 3890***, *R^2^ =14.6%*  Sprint run, jump (process) side gallop, catch, kick, overarm throw, and balance were not significant | MC was a significant predictor of future physical activity. Catching in boys was the best predictor of average daily steps. In girls the hop was the best predictor of daily average steps. |
| [52] De Souza et al. (2014) | Portugal | N/A | 2 (4 years) | 285 (143 M, 142 F) | T1: 6  T2: 10 | KTK  *Product* | ***Scores grouped by PA tertiles***  **Sedentary**  ***Males***  80.1 (21.7)  ***Females***  79.4 (24.8)  **Moderate**  ***Males***  91.1 (26.9)  ***Females***  87.7 (25.9)  **Very Active**  ***Males***  9765 (27.1)  ***Females***  99.7 (33.2) | | Godin and Shephard questionnaire  *Subjective,* total PA derived by multiplying the frequency of each PA category by corresponding MET value | | **Sedentary**  ***Males***  43.80(27.24)  ***Females***  46.26 (32.71)  **Moderate**  ***Males***  55.74 (37.53)  ***Females***  43.31 (34.11)  **Very Active**  ***Males***  48.46 (28.87)  ***Females***  42.47 (29.52) | T-test |  | **MC (T1)🡪 PA(T2)**  ***Males***  **Sedentary vs Moderate**  ***Males***  p = 0.381  ***Females***  p = 0.366  **Moderate vs Very Active**  ***Males***  p = 0.726  ***Females***  p = 0.325  **Sedentary vs Very Active**  ***Males***  p = 0.035*  ***Females***  p = 0.016** | MC was significantly different for children in the highest PA tertile compared to those in the lowest PA tertile in girls and boys. |
| [33] Dos Santos et al. (2018) | Portugal | N/A | 4 (T1 to T2 = 1 year)  T2 to T3 = 1 year  T3 to T4 = 1 year) | 245 (123 M, 122 F) | T1 = 6  T2 = 7  T3 = 8  T4 = 9 | KTK  *Product* | **Total Score**  ***Males***  T1: 111.0 (31.1)  T2: 140.5 (36.6)  T3: 164.6 (37.0)  T4: 182.1 (39.8)  ***Females***  T1: 99.5 (27.2)  T2: 130.9 (30.7)  T3: 157.8 (34.2)  T4: 174 (38.6) | | Godin and Shephard questionnaire  *Subjective,* total PA derived by multiplying the frequency of each PA category by corresponding MET value | | **Total MET Minutes**  ***Males***  T1: 51.4 (31.8)  T2: 42.1 (24.0)  T3: 47.7 (22.8)  T4: 49.3 (23.3)  ***Females***  T1: 41.2 (32.9)  T2: 37.8 (23.3)  T3: 34.8 (3.3)  T4: 33.3 (19.8) | Multilevel modelling |  | **PA🡪MC**  **Model 1**  B = 0.0002 ± 0.0002, p = .382 | Physical activity was not significantly associated with change over time in MC |
| [51]Fransen et al. (2014) | Belgium | N/A | 2 (2 years) | 501 (268 M, 233 F) | T1: 8.2 (1.2)  T1 Age Cohort 1: 6-7.99  T1 Age Cohort 2: 8.00-9.99 | KTK  *Product* | NR; participants stratified into tertiles based on level of MC | | Flemish Physical Activity Computerized Questionnaire  *Subjective* Total time spent in sport (hours per week x months per year), Time (hours per week) in club sport | | **T1 time spent in sports**  ***Males-Cohort 1***  Low MC: 21.7 (14.8)  Average MC: 26.1 (17.4)  High MC: 22.9 (21.4)  ***Males-Cohort 2***  Low MC: 34.2 (24.0)  Average MC: 32.2 (17.8)  High MC: 31.8 (16.1)  ***Females-Cohort 1***  Low MC: 20.7 (13.4)  Average MC: 24.5 (19.5)  High MC: 18.9 (12.6)  ***Females-Cohort 2***  Low MC: 20.7 (8.3)  Average MC: 27.5 (15.4)  High MC: 36.3 (3.8)  **T1 time spent in club sport**  ***Males-Cohort 1***  Low MC: 2.5 (1.7)  Average MC: 2.4 (1.1)  High MC: 2.2 (1.8)    ***Males-Cohort 2***  Low MC: 3.0 (1.4)  Average MC: 3.1 (1.7)  High MC: 3.4 (1.6)  ***Females-Cohort 1***  Low MC: 2.3 (1.9)  Average MC: 2.3 (1.9)  High MC: 2.1 (1.3)  ***Females-Cohort 2***  Low MC: 2.2 (0.8)  Average MC: 2.6 (1.5)  High MC: 3.4 (2.3)  **T2 time spent in sports**  ***Males-Cohort 1***  Low MC: 29.5 (16.2)  Average MC: 34.6 (15.3)  High MC: 36.7 (28.5)  ***Males-Cohort 2***  Low MC: 29.3 (14.1)  Average MC: 27.5 (15.4)  High MC: 37.8 (24.4)  ***Females-Cohort 1***  Low MC: 28.5 (28.8)  Average MC: 34.6 (15.3)  High MC: 16.7 (8.9)  ***Females-Cohort 2***  Low MC: 20.8 (15.2)  Average MC: 27.9 (17.6)  High MC: 41.2 (31.7)  **T2 time spent in club sports**  ***Males-Cohort 1***  Low MC: 2.7 (1.1)  Average MC: 3.5 (1.7)  High MC: 3.5 (2.7)  ***Males-Cohort 2***  Low MC: 2.8 (1.5)  Average MC: 3.5 (2.6)  High MC: 3.7 (2.4)    ***Females-Cohort 1***  Low MC: 2.9 (2.9)  Average MC: 2.2 (1.0)  High MC: 1.8 (1.0)  ***Females-Cohort 2***  Low MC: 2.2 (1.7)  Average MC: 2.9 (1.8)  High MC: 3.9 (2.8) | Repeated-measures ANOVA |  | **MC (T1) 🡪PA (T2)**  **Cohort 1 Time in sports**  **MC**  F = 0.73  **Time x MC**  F = 0.09  Differences between MC Groups Not reported  **Cohort 2**  **Time in sports**  **MC**  F = 3.51*  **Time x MC**  F = 0.66  **High MC vs Low MC**  High MC significantly more time in sport (total) than Low MC (presented in text; no quantitative result)  **Average MC vs High MC**  No difference (presented in text; no quantitative result)  **Average MC vs Low MC**  No difference (presented in text; no quantitative result)  **Cohort 1 time in club sports**  **MC**  F = 0.20  **Time x MC**  F = 0.01  Differences between MC Groups Not reported  **Cohort 2 time in club sports**  **MC**  F = 3.44*  **Time x MC**  F = 0.26  **High MC vs Low MC**  High MC significantly more time in club sport (hours/week) than Low MC (presented in text; no quantitative result)  **Average MC vs High MC**  No difference (presented in text; no quantitative result)  **Average MC vs Low MC**  No difference (presented in text; no quantitative result) | MC was not a significant predictor of time in sports for 6-8 year olds  MC was a significant predictor of sport (total and club) PA in 8-10 year olds |
| [56] Gu (2016) | United States | N/A | 2 (school year) | 256 (129 M, 127 F) | 5.4 (0.5) | Physical Education Metrics (hopping, sliding, dribbling, underhand throwing)  *Process* | **Locomotor**  T1: 11.8 (3.0)  **Object Control**  T1: 19.5 (4.7)  **FMS Index**  T1: 31.4 (6.3) | | Accelerometer (Actical)  *Objective*, 5 school days, Light PA, MVPA, and Vigorous PA | | **% Light PA**  T2: 67.4 (10.4)  **% Vigorous PA**  T2: 3.4 (3.4)  **% MVPA**  T2: 23.5 (14.4) | Correlation, regression | **MC (T1) 🡪PA (T2)**  **Locomotor🡪LPA**  r = -0.18**  **Locomotor🡪VPA**  r = 0.18**  **Locomotor🡪MVPA**  r = 0.21**  **Object Control🡪LPA**  r = -0.18**  **Object Control🡪VPA**  r = 0.18**  **Object Control🡪MVPA**  r = 0.21**  **FMS Index🡪LPA**  r = -0.23**  **FMS Index🡪VPA**  r = 0.22**  **FMS Index🡪MVPA**  r = 0.26** | **MC (T1) 🡪PA (T2)**  **Locomotor🡪 LPA**  B = -0.06**  β = -0.19**  **Locomotor🡪VPA**  B = 0.06**  β = 0.16**  **Locomotor🡪MVPA**  B = 0.07**  β = 0.20**  **Object Control🡪LPA**  B = -0.02  β = -0.11  **Object Control🡪VPA**  B = 0.03  β = 0.12  **Object Control🡪MVPA**  B = 0.03  β = 0.14  **FMS Index🡪MVPA**  β = 0.26**  FMS Index is not presented in relation to LPA and VPA. | FMS Index and Locomotor skills explained significant variance in physical activity (all intensities) Object control skills did not. |
| [55] Gu, Keller, Weiller-Abels, and Zhang (2018) | United States | N/A | 2 (school year) | 141 (72 M, 69 F) | 5.4 (0.5) | Physical Education Metrics (hopping, sliding, dribble, underhand throwing)  *Process* | NR | | Accelerometer (Actical)  *Objective*, 5 school days, MVPA and Sedentary behavior | | NR | SEM | **MC🡪PA**  r = 0.28** | **MC (T1)🡪PA (T2)**  β = 0.29** | Across one school year, motor skills were a statistically significant predictor of future physical activity |
| [58] Henrique et al. (2016) | Brazil | N/A | 2 (2 years) | 292 (158 M, 134 F) | 4.8 (0.79) | TGMD-2  *Process* | **Locomotor**  ***Test Sample***  T1: 10.07 (1.95)  ***Dropout Sample***  T1: 10.49 (2.08)  **Object Control**  ***Test Sample***  T1: 9.34 (2.25)  ***Dropout Sample***  T1: 9.75 (1.97) | | Sport participation defined as  organised physical activities (at least one hour per week)  *Subjective,* parent questionnaire developed by researchers | | NR | Logistic Regression |  | **MC (T1)🡪PA (T2)**  **Locomotor**  OR = 1.21*  **Object Control**  OR = excluded from analysis | Baseline locomotor skills, but not object control skills were a significant predictor of sport participation two years later. |
| [40] Henrique et al. (2018) | Portugal | N/A | 4 (1 year) | 245 (123 M, 122 F) | T1: 6.46– 9.46 | KTK  *Product* | **Walking backwards**  ***Males***  T1: 29.0 (13.8)  T2: 37.9 (13.9)  T3: 43.3 (13.7)  T4: 48.3 (13.0)  ***Females***  T1: 28.6 (15.5)  T2: 36.6 (14.7)  T3: 40.5 (11.5)  T4: 46.6 (13.7)  **Jumping sideways**  ***Males***  T1: 32.0 (9.8)  T2: 37.7 (11.0)  T3: 44.6 (13.9)  T4: 54.7 (13.2)  ***Females***  T1: 28.6 (8.9)  T2: 36.0 (9.8)  T3: 43.9 (13.6)  T4: 52.5 (12.0)  **Hopping for height**  ***Males***  T1: 18.6 (11.8)  T2: 27.5 (16.0)  T3: 38.0 (17.9)  T4: 43.5 (18.5)  ***Females***  T1: 15.3 (10.1)  T2: 23.0 (13.6)  T3: 35.3 (17.4)  T4: 38.4 (17.8)  **Moving sideways**  ***Males***  T1: 30.5 (5.0)  T2: 36.9 (6.5)  T3: 40.3 (5.5)  T4: 41.9 (7.6)  ***Females***  T1: 27.6 (5.2)  T2: 35.7 (6.5)  T3: 39.0 (7.2)  T4: 40.4 (6.9)  **Motor quotient**  ***Males***  T1: 94.9 (13.9)  T2: 98.8 (16.1)  T3: 97.0 (16.3)  T4: 88.0 (17.4)  ***Females***  T1: 81.7 (14.3)  T2: 89.5 (15.0)  T3: 92.9 (16.6)  T4: 79.2 (16.9) | | Godin and Shephard questionnaire  *Subjective,* total PA score derived by multiplying the frequency of each category by corresponding MET value | | **Total Physical Activity**  ***Males***  T1: 51.4 (31.8)  T2: 42.1 (24.0)  T3: 47.7 (22.8)  T4: 49.3 (23.3)  **Total Physical Activity**  ***Females***  T1: 41.2 (32.9)  T2: 37.8 (23.3)  T3: 34.8 (31.3)  T4: 33.3 (19.8) | T-test |  | **PA (T1) 🡪 MC (T4)**  **High vs Low MC and Physical Activity**  t = 0.56, p = 0.60 | Physical activity at age 6 did not statistically differ between individuals with high and low levels of MC at age 9. |
| [41] Herrmann, Heim, and Seelig (2017) | Germany | N/A | 2 (8 months) | 1031^1^ (557 M, 474 F)  436^2^ (209 M, 227 F) | *T1:* 6.8 (0.4)  *T2:* 7.5 (0.4) | MOBAK-1  *Product* | **Self-Moving**  T2: 5.7 (1.8)  **Object-Moving**  T2: 5.4 (1.9) | | Parental questionnaire  *Subjective,* sum of times per week individual and team sports training occurred | | T1: 48% of the 436 children practiced sport  Team sport  12%; 2.24 times/week (0.72)    **Individual Sport**  31 %; 1.67 times/week (0.79)  **Both team and individual sport**  6%; 1.72 times/week (0.61) for team sports and individual sport 1.24 times/week (0.44) | Autoregressive SEM |  | **PA🡪MC**  **Individual sports and locomotor/stability**  ß = 0.35**  **Team sports and locomotor/stability competence**  ß = -.01  **Individual sports and object control skills**  ß = -.10  **Team sports -and object control skills**  ß = .21** | MC in self-moving (i.e., locomotor and stability) skills is predicted by the participation in enhanced school sport practice and the frequency of practice of afterschool individual sports.  MC in object-moving (i.e., object-control) skills is predicted only by the frequency of practice of afterschool team sports. |
| [47] Jaakkola, Yli‐Piipari, Huotari, Watt, and Liukkonen (2016) | Finland | N/A | 2 (6 years) | 333 (133 M, 200 F) | *T1:* 12.4 (0.3)  *T2:* 18.3 (0.3) | Flamingo standing test, leaping test, and ﬁgure-8 test  *Product* | **Composite score (z-score)**  T1: 0.04 (0.57***)*** | | Short form of International Physical Activity Questionnaire (Ainsworth et al., 2006)  *Subjective,* frequency and duration of LPA, MPA, and VPA 10-min bouts to calculate weekly amounts in each intensity | | ***Follow-up only***  **MET (min/week)**  Total = 4810.4 (2665.9)  **Light PA**  Total = 448.6 (290.1)  **Moderate PA**  Total = 214.6 (127.2)  **Vigorous PA**  Total = 309.9 (164.3) | Hierarchical multiple linear regressions | **MC (T1)🡪PA (T2)**  METs  r = 0.38***  Light PA  r = 0.27***  Moderate PA  r = 0.27***  Vigorous PA  r = 0.43*** | **MC (T1)🡪PA (T2)**  **METs**  β = 0.34***  **LPA**  β = 0.27***    **MPA**  β = 0.17**  **VPA**  β = 0.38*** | MC is a significant predictor of future physical activity in a variety of intensities |
| [36] Jaakkola, Yli-Piipari, et al. (2019) | Finland | N/A | 2 (1 year) | 491 (216 M, 275 F) | *T1:* 11.3 (0.3) [Note - article reports 3 different M ages at baseline: 11.26 (Table 1), 11.27 (abstract), 11.36 (method]  *T2:* 12.3 (0.3) | 5‐leaps test (locomotor), throwing‐catching combination test (manipulative), two‐legged jumping from side to side test (stability)  *Product* | | **5-leaps test**  T1: 7.8(0.9)  T2: 8.37(1.1)  **Throwing-catching**  T1: 12.6(4.8)  T2: 14.3(4.3)  **Two-legged jumping**  T1: 36.6(6.5)  T2: 40.4(7.1) | Accelerometer (GT3X+)  *Objective,* 7 day physical activity | | **MVPA**  T1: 63.2(23.1)  T2: 58.7(21.3) | Cross-lagged Structural Equation Model panel analysis | **MC (T1) 🡪 PA (T2)**  ***Females***  **Stability and MVPA**  r = 0.23**  **Manipulative and MVPA**  r = 0.17*  **Locomotor and MVPA**  r = 0.23**  ***Males***  **Stability and MVPA**  r = 0.26**  **Manipulative and MVPA**  r = 0.29***  **Locomotor Skills and MVPA**  r = 0.41***  **PA (T1)🡪MC (T2)**  ***Females***  **MVPA and Stability**  r = 0.15*  **MVPA and Manipulative**  r = 0.30***  **MVPA and Locomotor**  r = 0.22***  ***Males***  **MVPA and Stability**  r = 0.43***  **MVPA and Manipulative**  r = 0.34***  **MVPA and Locomotor**  r = 0.42*** | **MC (T1) 🡪 PA (T2)**  No significant cross-lagged panel associations between any measure of MC and future PA for boys or girls  **PA (T1)🡪MC (T2)**  **MVPA and Manipulative**  ***Females***  ß = 0.11*  No significant cross-lagged panel associations between MVPA and stability or locomotor skills in girls.  No significant cross-lagged panel associations between MVPA and future MC in boys. | Findings revealed that MC at age 11 was not a significant predictor of MVPA at age 12 for boys or girls.  MVPA was only a predictor of future manipulative skills in girls. These findings did not hold for locomotor or stability skills in girls.  This study did not find any significant relationship between MVPA and future MC of any kind for boys. |
| [35] Jaakkola, Hakonen, et al. (2019) | Finland | N/A | 2 (1 year) | 336 (173 M, 163 F) | 12.0 (0.4) | 5-leaps test (locomotor), throwing-catching combinationtest (manipulative)  *Product* | **Throwing–catching**  ***Males***  T1:14.8 (4.7)  T2: 13.2(4.6)  ***Females***  T1: 13.9 (4.6)  T2: 12.9 (4.5)  **5-leaps test**  ***Males***  T1: 8.5 (1.2)  T2: 9.0 (1.2)  ***Females***  T1: 8.2 (0.9)  T2: 8.3 (0.9) | | Accelerometer (ActiGraph GT3X+,wGT3X+)  *Objective,* 7 day physical activity | | **MVPA (min/day)**  ***Males***  T1: 61.7 (26.6)  T2: 57.9 (26.4)  ***Females***  T1: 47.5 (17.4)  T2: 41.6 (18.5) | SEM, correlation | ***PA (T1) 🡪MC (T2)***  **Males**  *MVPA and Manipulative*  r = 0.30**  *MVPA and Locomotor*  r = 0.18  **Females**  *MVPA and Manipulative*  r = 0.13  *MVPA and Locomotor*  r = 0.32**  **MC (T1) 🡪PA (T2)**  ***Males***  Manipulative and MVPA  r = 0.23*  Locomotor and MVPA  r = 0.25*  ***Females***  Manipulative and MVPA  r = 0.12  Locomotor and MVPA  r = 0.11 | **PA (T1) 🡪MC (T2)**  ***Males***  No significant cross-lagged panel pathways for boys for *locomotor or manipulative skills*  **MVPA and Locomotor**  ***Females***  B = 0.14*, Standard error = 0.064  No significant cross-lagged panel analysis for girls for MVPA and manipulative skills  **MC (T1) 🡪PA (T2)**  No significant cross-lagged association between T1 locomotor or manipulative skills and T2 PA for boys or girls | Girls who engage in MVPA at the end of primary school (Grade 6) achieve higher leaping skill scores one year after during transition to secondary school (Grade 7).  Throwing-catching did not show a significant relationship with future physical activity for boys or girls.  There were no statistically significant cross-lagged associations between MVPA and MC for boys. |
| [38] Jekauc, Wagner, Herrmann, Hegazy, and Woll (2017) | Germany | N/A | 2 (6 years) | 698 (335 M, 363 F) | *T1:* 14.2 (2.0)  *T2:* 20.5 (2.0) | KTK (jumping side-to-side test, single leg stance, and backward balancing)  *Product* | **Jumping side-to-side**  T1: 34.2 (6.2)  T2: 39.9 (6.8)  **Single leg stance**  T1: 4.5 (5.4)  T2: 2.4 (3.8)  **Backward balancing**  T1: 34.8 (9.2)  T2: 39.2 (8.0) | | MoMo Physical Activity Questionnaire  *Subjective,* type, duration, frequency and seasonality of sports club participation | | **Sport MVPA (min/week)**  T1:110.0 (144.9)  T2:71.6 (126.1) | Multiple Regression |  | **MC🡪PA**  β = -0.2  **PA🡪MC**  β = 0.1 | There was no statistically significant direct effect in either direction for sport PA and MC. |
| [54] Larsen, Kristensen, Junge, Rexen, and Wedderkopp (2015) | Denmark | N/A | 2 (3 years) | 673 (298 M, 375 F) | 9.2 (1.4) | Backward balance, Precision Throw | **Balance**  ***Males***   T1: 45.0 (13.0)  ***Females***  T1: 48.6 (12.7)  ***Total***  T1: 47.0 (12.9)  **Precision throw**  ***Males***  T1: 14.8 (4.5)  ***Females***  T1: 11.7 (4.8)  ***Total***  T1: 13.2 (4.9) | | Accelerometer (ActiGraph GT3X)  *Objective,* 7 day wear time, MVPA | | **Mean % MVPA (unweighted estimates)**  ***Males***  T1: 9.0 (2.4)  T2: 7.9 (3.0)  ***Females***  T1: 7.4 (2.3)  T2: 6.1 (2.2)  ***Total***  T1: 8.1 (2.4)  T2: 6.9 (2.7) | Linear Regression |  | **MC (T1) 🡪PA (T2)**  **Throw and MVPA**  B = 0.04, 95% CI (-0.006, 0.09)  β = 0.20, 95% CI (-0.03, 0.43)  **Balance and PA**  B = 0.01, 95% CI (-0.006, 0.026)  β = 0.13, 95% CI (-0.07,0.33) | Balance and throwing were not significant predictors of future MVPA |
| [37] Lima et al. (2017) | Denmark | N/A | 3 (T1 to T2 = 3 years,  T2 to T3 = 4 years) | *T1 =* 696  *T2 =* 617  *T3 =* 513 | *T1:* 6.8 (0.4)  *T2:* 9.6 (1.1)  *T3:* 13.4 (0.3) | KTK  *Product* | **KTK Total Score**  ***Males***  T1: 120.1 (28.4)  T2: 194.8 (34.9)  T3: 251.4 (29.9)  ***Females***  T1: 118.2 (26.8)  T2: 195.6 (34.4)  T3: 247.3 (28.8)  ***Total Sample*** T1: 119.2 (27.7)  T2: 195.2 (34.6)  T3: 249.4 (29.4) | | Accelerometer (ActiGraph 7164 at T1 and ActiGraph GT1M at T2, T3)  *Objective,* 4 days of MVPA and VPA reported | | **MVPA**  ***Males***  T1: 82.7 (29.6)  T2: 78.1 (25.8)  T3: 59.9 (26.5)  ***Females***  T1: 69.7 (21.8)  T2: 67.5 (24.1)  T3: 6.0 (20.1)  ***Total Sample***  T1: 76.5 (27.0)  T2: 73.0 (25.5)  T3: 53.2 (24.6)  **VPA**  ***Males***  T1: 31.1 (15.6)  T2: 32.6 (15.8)  T3: 23.3 (14.1)  ***Females*** T1: 25.2 (11.2)  T2: 27.1 (13.8)  T3: 8.0 (11.8)  ***Total Sample***  T1: 28.3 (14.0)  T2: 29.9 (15.1)  T3: 20.8 (13.3) | SEM |  | **PA (T1) 🡪MC (T3)**  **VPA and MC (direct)**  β = 0.095*, 95% CI (0.02,0.17)  **MVPA and MC (direct)**  β = 0.07, 95% CI (-0.005, 0.142)  **MC (T1) 🡪PA (T3)**  **MC and VPA (direct)**  β = 0.08*, 95% CI (0.018, 0.149)  **MC and MVPA (direct)**  β = 0.06, 95% CI (-0.005, 0.126) | Results of this study indicated a reciprocal, longitudinal relationship occurred between vigorous physical activity and motor competence across 7 years. |
| [44] Lloyd, Saunders, Bremer, and Tremblay (2014) | Canada | N/A | 4  (T1 to T2 = 5 years; T2 to T3 = 5 years; T3 to T4 = 10 years) | *T1:* 17 (5 M, 12 F)  *T2:* 10 (4M, 6F)  *T3:* 13 (4 M, 9 F)  *T4:* 17 (5 M, 12 F) | *T1:* 6.8 (0.4) *T2:* 11.9 (0.4) *T3:* 16.8 (0.3) *T4:* 26.8 (0.4) | TGMD (T1 and T2 only)  *Process* | **Total Score** ***High Motor Proficiency***  T1: 26.33 (2.34)  T2: 36.50 (2.12)  ***High Motor Proficiency***  T1: 38.18 (2.56)  T2: 40.75 (2.96)  **Locomotor**  ***Low Motor Proficiency***  T1:18.50 (3.21)  T2: 24.00 (1.41)  ***High Motor Proficiency***  T1: 22.91 (2.74)  T2: 24.63 (2.30)  **Object Control**  ***Low Motor Proficiency***  T1: 9.50 (1.52)  T2: 12.50 (0.71)  ***High Motor Proficiency***  T1: 15.27 (2.65)  T2: 26.23 (2.48) | | International Physical Activity Questionnaire Craig et al., 2003) ; T4 only)  *Subjective,* total physical activity per week | | ***T4 Only***    **Leisure time MVPA (min/ wk)** Low MC: 31.7 (60.1)  High MC: 62.7 (57.6)   **Leisure time VPA (min/wk)** Low MC: 121.7 (141.2)  High MC: 272.7 (210.2)  **Total leisure time PA (min/wk)**  Low MC: 413.3 (293.0)  High MC: 526.4 (343.7)  **Total PA (min/wk)** Low MC: 1178.3 (1011.2)  High MC: 1206.8 (887.7) | Correlation |  | **MC (T1) 🡪PA (T4)**  ***Total sample***  **Total MC and Total PA**  r = 0.09  **Locomotor and PA**  r = 0.34  **Object control and PA**  r = -0.25  ***High MC Group***  **T1 Total MC and T4 Leisure Time PA**  r = 0.77** (reported in text)  **T1 Locomotor and T4 Leisure Time PA**  r = 0.78** (reported in text)  **T1 Object Control and T4 Leisure Time PA**  Not reported  Low MC group results not reported. | For the total sample, MC (total, locomotor, object control) were not significant predictors of PA at age 26.  Motor skill proficiency (Total and Locomotor) at age 6 was positively associated with leisure time PA at age 26 in the High MC group.  Low MC results were not reported, thus conclusions cannot be made. |
| [49] Lopes et al. (2019) | Portugal | N/A | 2 (2 years) | 103 (50 M, 53 F) | *T1:* 13.5 (0.9)  *T2:* 14.6 (0.3) | KTK  *Product* | **Total Scores**  *T1*: 89.2 (16.8)  *T2:* 100.5 (16.3) | | Accelerometer (ActiGraph GT1M)  *Objective,* 5 days (3 weekdays, 2 weeknights) | | **Total PA(%)** *T1*: 24.4 (4.2)  *T2*: 23.0 (4.5)  **Light PA (%)**  T1: 16.6 (2.4)  T2: 15.2 (2.5)  **Moderate PA (%)** T1: 4.4 (1.4)  T2: 4.5 (1.8)  **MVPA (%)**  T1: 7.8 (2.8)  T2: 7.7 (3.1)    **Vigorous PA (%)** T1: 3.3 (1.5)  T2: 3.3 (1.7) | Linear Regression |  | **MC (T1) 🡪PA (T2)**  **MC and LPA**  *Unadjusted*  B =−0.24  *Adjusted*  B = 0.02   **MC and MPA**  *Unadjusted*  B = 0.02  *Adjusted*  B = 0.03*   **MC and MVPA** *Unadjusted*  B = 0.04*  *Adjusted*  B = 0.05*  **MC and VPA**  *Unadjusted*  B = 0.02* *Adjusted*  B = 0.02   **MC and Total PA** *Unadjusted*  B = 0.01  *Adjusted*  B = 0.07* | MC in adolescence is positively associated with moderate PA, moderate-to-vigorous PA and total PA |
| [45] McIntyre, Parker, Chivers, and Hands (2018) | Australia | N/A | 4 (T1 to T2 = 6 months,  T2 to T3 = 6 months,  T3 to T4 = 6 months | 201 (112 M, 89 F) | T1: 7.2 F; 7.2 M | Fundamental Movement Skills Teacher Resource Manual (standing broad jump, run, overhand throw, line walk; EDWA, 2001)  *Process* | | **Total Scores**  ***Males***  *T1:* 17.7 (3.2)  *T2:* 18.4 (2.5)  *T3:* 19.0 (2.6)  *T4:* 19.2 (2.6)  ***Females*** *T1:* 15.6 (2.3)  *T2:* 16.5 (2.4)  *T3:* 17.0 (2.1)  *T4:* 17.3 (2.0) | Pedometers (Yamax SW-200)  *Objective,* daily step counts over 7 days | **Daily Steps**  ***Males*** *T1:* 14134 (3531)  *T2:* 15189 (7054)  *T3:* 14761 (3689) *T4:* 14645 (3472)  ***Females***  *T1:* 12250 (3041)  *T2:* 11553 (4750)  *T3:* 11850 (2775) *T4:* 11785 (2775) | | Linear Mixed Model |  | **MC🡪PA**  β = 136.18* (SE = 58.51). | MC was a significant predictor of physical activity longitudinally |
| [43] Reyes et al. (2019) | Portugal | N/A | 3 (1 year between assessments) | 344 (174 M, 170 F) | T1: 4-9  T2: 5-10  T3: 6-11 | KTK  *Product* | | **Total Scores**  ***Males***  5-year olds: 64.1 (23.3)  6-year olds: 102.2 (33.2)  7-year olds: 130.1 (37.0)  8-year olds: 153.1 (34.8)  9-year olds:  177.0 (38.3)  10-year olds: 199.8 (29.8)  11-year olds: 213.8 (41.8)  ***Females***  5-year olds: 70.2 (19.9)  6-year olds: 103.0 (29.7)  7-year olds: 129.2 (28.6)  8-year olds: 155.6 (31.9)  9-year olds: 175.9 (31.6)  10-year olds: 207.7 (37.0)  11-year olds: 219.9 (42.0) | Accelerometer (GT3X+)  *Objective,* Seven days | **MVPA**  ***Males***  5-year olds: 83.6 (23.8)  6-year olds: 82.8 (21.8)  7-year olds: 77.3 (23.5)  8-year olds: 74.0 (23.4)  9-year olds:  71.1 (20.7)  10-year olds: 68.5 (22.6)  11-year olds: 70.3 (20.1)  ***Females***  5-year olds: 65.3 (20.8)  6-year olds: 62.2 (19.6)  7-year olds: 60.3 (17.4)  8-year olds: 59.6 (19.7)  9-year olds: 55.0 (15.3)  10-year olds: 57.8 (18.3)  11-year olds: 60.2 (19.8) | | Multilevel Hierarchical Linear Models |  | **PA🡪MC**  β = 0.03 | MVPA is not a significant predictor of future gross motor coordination in boys and girls across a three year period. |
| [57] Schmutz et al. (2018) | Switzerland | N/A | 2 (1 year) | 498 (268 M, 230 F) | T1: 3.9 (0.7)  T2: 4.9 (0.7) | Zurich Neuromotor Assessment  Walking, running, jumping, hopping  *Process and Product* | | **Total Scores**  *T1:* 0.0 (1.0)  *T2:* 0.1 (1.1)  Composite z-score | Accelerometer (ActiGraph wGT3X-BT)  *Objective,* Seven days | **Total PA (counts per minte)**  *3 yr:* 577.8 (136.2)  *4 yr:* 631.3 (154.1)  *5 yr:* 651.6 (149.5)  *6 yr:* 683.3 (170.7)  **MVPA (min/ day)**  *3 yr:* 81.2 (26.9)  *4 yr:* 93.9 (28.0)  *5 yr:* 99.9 (28.8)  *6 yr:* 107.2 (31.2) | | Linear Mixed Models |  | **MC (T1) 🡪PA (T2)**  **Total PA (counts per minte)**  β = 10.7*, 95% CI [0.1, 21.3]  **MVPA**  β = 2.2* 95% CI [0.4, 4.2]  **Change in Total PA (counts per minute)**  β = -16.8  **Change in MVPA**  β = -3.0 | Gross motor skills were longitudinally associated with future PA.  However, gross motor skills were not a significant predictor for the change in PA levels shown across a one year period. |
| [50] Smith, Fisher, and Hamer (2015) | United Kingdom | N/A | 3 (T1 to T2 = 6 years,  T2 to T3 = 26 years) | 3073-4879 | T1: 10 years | Throwing test, standing on one leg, backward walking  *Product* | NR; based on overall score participants were grouped into low, medium, or high MC at T1 | | Survey (unspecified; T2 and T3 only)  *Subjective,* list of activities provided and frequency of participation for each activity | | NR | Logistic Regression, odds ratio |  | ***MC🡪PA***  **MC (T1) and PA (T2)**  Low MC: OR = 1.0  Medium MC: OR = 0.98  High MC: OR = 1.16-1.20  **MC (T1)and PA (T3)**  Low MC: OR = 1.0 (Ref)  Medium MC: OR = 1.08-1.11  High MC: OR = 1.18* -1.22 | Gross motor coordination (for those in the high competence group) was associated with participation in physical activity at age 42. There was no association at age 16. |
| [46] Venetsanou and Kambas (2017) | Greece | N/A | 2 (10 years) | 106 (47 M, 59 F) | *T1:* 5.0 (0.6)  T2: 14.5 (0.8) | BOT-Short Form  *Product* | NR; Stratified into Average, Above Average, and High MC groups per manual guidelines | | Pedometer (Omron HJ-720IT)  *Objective,* dailysteps taken across 7 day wear period | | **Daily Steps**  ***Males***  Average MC: 11067 (739)  Above Average MC: 10398 (1895)  High MC: 12112 (858)  ***Females*** Average MC: 7820 (1841)  Above average MC: 10537 (1850)  High MC: 11810 (456)  ***Total***  Average MC: 9119 (2192)  Above average MC: 10509 (1849)  High MC: 12019 (766) | ANOVA |  | **MC (T1) 🡪PA (T2)**  F = 28.63*** η2=.253 | MC was significantly associated with physical activity 10 years later.  High MC group had highest levels of PA. |
| [53] Wagner, Jekauc, Worth, and Woll (2016) | Germany | N/A | 2 (6 years) | 940 (462 M, 478 F) With and without motor coordination problems. 825 w/o 418 and 407 | *T1:* 8.1 (1.5)  *T2:* 14.4 (1.5) | MoMo test battery items (backwards walk, side to side jumping, one leg balance)  *Product* | NR; groups stratified according to MC level | | MoMo-Physical Activity Questionnaire  *Subjective*club sport participation | | NR | Logistic Regression |  | **MC (T1) 🡪PA (T2)**  B = 0.43*  OR = 1.53 | Children with higher MC were more likely to have higher club sport participation. |
| ***Experimental Studies (MC🡪PA)*** | | | | | | | | | | | | | | | |
| [59] Cohen, Morgan, Plotnikoff, Barnett, and Lubans (2015) | Australia | Reported elsewhere (Lubans et al., 2012)  Dose:  Framework/ Theory: Socioecological Model  Approach: Teaching professional learning, student leadership, school committee and physical activity policies, provision of equipment, parental engagement, and school-community support.  Control: Usual physical education and school sport programs | 2 (1 year) | 460 (212 M, 248 F) | 8.5 (0.6) | TGMD-2  *Process* | NR; Cohen et al. 2014 | | Accelerometers (ActiGraph GT3X+)  *Objective,* 7 day wear with 4 valid days needed to calculate time spent in MVPA | | NR; Cohen et al., 2014 | Multilevel linear analysis |  | **MC🡪PA**  **Mediated Effect**  **Locomotor**  Product of Coefficient estimate = 0.86, 95% CI (-0.12, 2.44)  **Object Control**  Product of Coefficient estimate= 1.60, 95% CI (-1.04, 4.50)  **FMS**  Product of Coefficient estimate= 2.09*, 95% CI (0.01, 4.55) | Overall FMS competency mediated the effect of the SCORES intervention on physical activity. Locomotor skills and object control skills did not meet criteria for mediations effects. |
| [60] McGrane, Belton, Fairclough, Powell, and Issartel (2018) | Ireland | *Dose:* 9 month duration. Dose in lessons unclear  *Framework/Theory:* NR  *Approach:* School-based intervention with four components: (1) health-related activity and FMS in PE, (2) Parents and guardians educated about health benefits of PA,  (3) 2 teacher/ staff workshops with the main objective to promote PA participation among staff and students during school time, and (4) website | 3 (T1 to T2 = 8 months,  T2 to T3 = 4 months) | 482 (246 M, 236 F) | 12.8 (0.4) | TGMD-2  TGMD (skip, vertical jump)  Victorian Fundamental Movement Skills Manual (balance)  *Process* | **Object control**  ***Intervention***  T1: 36.7 (4.4)  T2: 38.7 (7.0)  T3: 42.5 (4.5)  ***Control***  T1: 37.4 (4.1)  T2: 36.1 (6.4)  T3: 40.1 (5.4)  **Locomotor**  ***Intervention***  T1: 52.1 (5.9)  T2: 50.3 (14.8)  T3: 57.1 (7.4)  ***Control***  T1: 51.5 (5.7)  T2: 48.7 (12.9)  T3: 54.5 (7.6)  **Total Scores**  ***Intervention***  T1: 95.1 (8.4)  T2: 86.8 (6.0)  T3: 99.6 (11.7)  ***Control***  T1: 94.7 (8.5)  T2: 83.9 (22.1)  T3: 94.5 (12.0) | | Accelerometer (GT3X, or GT3X+)  *Objective,* 9 days worn, middle 7 days used, daily MVPA reported | | **MVPA (min/day)**  ***Intervention***  T1: 52.6 (19.2)  ***Control***  T1: 53.6 (23.8)  Participants grouped into active (≥ 60 min/day MVPA) or inactive (<60 min/day MVPA | Multilevel linear regressions |  | **Locomotor**  ***Active***  β  **=** 2.18*** (CI 1.12 to 3.24)  ***Inactive***  β = 2.07*** (CI 1.03 to 3.11)  **Object Control**  ***Active***  β **=** 1.95*** (CI 0.95 to 2.95)  ***Inactive*** β = 2.13*** (CI 1.07 to 3.19)  **Total**  ***Active***  β = 4.03*** (CI 2.09 to 5.97)  ***Inactive*** β = 4.06*** (CI 2.14 to 5.98) | Intervention had significant and positive effects regardless of physical activity level, which implies PA didn’t have a role in MC changes. |
| * Reported within article, p < 0.05  ** Reported within article, p < 0.01  *** Reported within article, p < 0.001  ^1^ = Participants with full motor competence data  ^2^ = Participants with full motor competence data and sport participation data (i.e., physical activity) Note.CI = Confidence intervalF = FemaleKTK = Körperkoordinationstest Für KinderM = MaleMABC = Motor Assessment Battery for ChildrenMC = Motor competenceMET = Metabolic equivalentMVPA = Moderate-to-vigorous physical activityN/A = Not applicableNR = Not reportedPA = Physical activitySD = Standard deviationSEM = Structural equation modelingTGMD = Test of Gross Motor Development | | | | | | | | | | | | | | | |
