# Supplementary material for: Through the Looking Glass: A Systematic Review of Longitudinal Evidence, Providing New Insight for Motor Competence and Health
Source: Sports Med. 2021 Aug 31;52(4):875–920. doi: 10.1007/s40279-021-01516-8 (PMC8938405; doi:10.1007/s40279-021-01516-8)
Supplement: Supplementary file 3 — Supplementary file3 (DOCX 37 kb) [file 40279_2021_1516_MOESM3_ESM.docx]

| **Supplementary Table 3. Motor Competence and Weight Status Results** | | | | | | | | | | | | |
| --- | --- | --- | --- | --- | --- | --- | --- | --- | --- | --- | --- | --- |
| ***Longitudinal Studies*** | | | | | | | | | | | | |
| **Study** | **Country** | **Intervention description** | **Timepoints # (Duration)** | **Sample #**  ***(M, F)*** | **Age (SD)** | **MC measure** | **MC scores at each timepoint**  ***M (SD)*** | **Weight status measure** | **Weight status at each timepoint**  ***M (SD)*** | **Analysis** | **Pathway tested and values** | **Overall findings** |
| [42] Antunes et al. (2016) | Portugal | N/A | 2 (6 years) | 158 (83 M, 75 F) | T1:  Group 1: 6  Group 2: 7  Group 3: 8  T2:  Group 1: 12  Group 2: 13  Group 3: 14 | KTK  *Product*  TGMD-2  *Process* (assessed in relation to MC so not relevant to this review). | **Walking backwards**  ***Males***  T1  Group 1: 40.0(11.3)  Group 2: 44.5(11.1)  Group 3: 49.1(12.1)  T2  Group 1: 59.9(12.1)  Group 2: 55.8(13.0)  Group 3: 64.2 (9.4)  ***Females***  T1  Group 1: 34.3(12.3)  Group 2: 44.0(10.4)  Group 3: 46.3(12.7)  T2  Group 1: 49.0(16.9)  Group 2: 56.9(9.6)  Group 3: 56.5(13.8)  **Hopping**  ***Males***  T1  Group 1: 21.4(10.2)  Group 2: 29.4(12.4)  Group 3: 35.2(7.7)  T2  Group 1: 61.1(14.4)  Group 2: 60.9(16.0)  Group 3: 70.0 (9.6)  ***Females***  T1  Group 1: 19.4(11.1)  Group 2: 29.3(8.8)  Group 3: 33.2(13.9)  T2  Group 1: 51.7(12.9)  Group 2: 54.8(10.6)  Group 3: 52.4(11.7)  **Jumping sideways**  ***Males***  T1  Group 1: 31.0(8.8)  Group 2: 34.3(7.8)  Group 3: 42.1(7.3)  T2  Group 1: 70.8(11.4)  Group 2: 68.7(15.0)  Group 3: 78.1(7.3)  ***Females***  T1  Group 1: 31.3(7.2)  Group 2: 41.7(13.1)  Group 3: 42.6(12.2)  T2  Group 1: 64.2(14.6)  Group 2: 67.6(11.2)  Group 3: 69.7(11.6)  **Moving sideways**  ***Males***  T1  Group 1: 30.7(4.8)  Group 2: 34.1(4.7)  Group 3: 37.4(4.2)  T2  Group 1: 50.0 (6.4)  Group 2: 49.1 (7.5)  Group 3: 54.2 (5.2)    ***Females***  T1  Group 1: 28.5(5.5)  Group 2: 32.9(4.9)  Group 3: 34.5(5.5)  T2  Group 1: 45.7(10.5)  Group 2: 50.4 (7.1)  Group 3: 50.3 (6.7) | General  (height, body mass); Skin-folds (abdominal, biceps, calf, subscapular, suprailiac, thigh, and triceps  Procedures from the ‘‘Leuven Growth Study—Growth and Fitness of Flemish Girls’’ (Claessens et al., 1990). | **Body mass**  ***Males***  *Group 1*  T1: 23.3 (3.7)  T2:45.6 (8.0) *Group 2*  T1: 26.9 (4.0)  T2: 51.1 (11.3)  *Group 3*  T1: 31.9 (7.9) T2: 61.2 (16.6)  ***Females***  *Group 1*  T1: 23.3 (5.4)  T2: 47.2 (12.9)  *Group 2*  T1: 26.5 (5.2)  T2: 50.0 (8.3)  *Group 3*  T1: 30.5 (6.7)  T2: 54.5 (10.4)  **Sum of skin-folds**  ***Males***  *Group 1*  T1: 35.9 (14.3)  *Group 2*  T1: 39.8 (21.8)  *Group 3*  T1: 49.5 (23.4)    ***Females***  *Group 1*  T1: 48.1 (28.1)  *Group 2*  T1: 44.5 (17.1)  *Group 3*  T1: 59.7 (29.6) | Multiple linear regression | **WS (T1) 🡪MC (T2)**  ***Males***  *Group 1*  Skinfolds or body mass not predictive of any skills  *Group 2*  Skinfolds or body mass not predictive of any skill.  *Group 3*  Skinfolds🡪Hop  β = -0.55*  Skinfolds🡪Move sideways  β = -0.49*  ***Females***  *Group 1*  Skinfolds🡪 Hop  β = -0.64*  Skinfolds not predictive of Walking backwards or Jumping sideways or Move Sideways  Body mass not predictive of any skill.  *Group 2*  Body mass🡪Move sideways  β = -0.41*  Body mass not predictive of Hop, Walking backwards, Jumping sideways.  Skinfolds not predictive of any skill.  *Group 3*  Body mass🡪Walking backwards  β = -0.47*  Body mass🡪Jump sideways  β = -0.39*  Body mass not predictive of Moving sideways.  Skinfolds not predictive of any skill. | General characteristics of growth in early childhood showed limited correlations with MC performance 6 years later.  For boys, early childhood skinfolds were related to hopping and moving sideways.  For girls, body mass in early childhood was correlated to several individual MC six years later including moving sideways, walking backwards, and jumping sideways; skinfolds were related to hopping. |
| [48] Bryant, James, Birch, and Duncan (2014) | United Kingdom | N/A | 2 (1 year) | 281 (129 M, 152 F) | T1: 8.9 (1.4)  T2: 9.8 (1.4) | Process checklist from New South Wales “*Move it Groove it*” (Sprint Run, Side Gallop, Hop, Kick, Catch, Overarm Throw, Vertical Jump and Static Balance)  *Process*  Objective Measurements (sprint run, vertical jump)  *Product* | NR | Weight, height, BMI (Cole, Freeman, & Preece, 1995).  Body Fat % was calculated using skinfold assessments (tricep and medial calf). | **BMI**  T1: 17.5 (2.9)  T2: 17.7 (3.4)  **Body Fat %**  T1: 14.6 (2.9)  T2: 21.9 (8.5) | Multiple linear regression | **MC (T1) 🡪 WS (T2)**  ***Males***  Catch🡪 BF%  β = -8.2*  Non-significant predictors of weight status: Sprint Run (both process and product assessment), Side Gallop, Hop, Kick, Overarm Throw, Vertical Jump (both process and product) and Static Balance  ***Females***  No MC skills were significant predictors of weight status  **WS (T1) 🡪MC (T2)**  ***Males***  Body Fat % 🡪 Jump (Process)  β = -1.6***  Body Fat % 🡪 Balance  β = -1.6***  Body Fat % 🡪 Jump Height  β = -2.4**    BMI🡪Jump Height (Product)  β = -1.7*  Skills not retained in final model for BMI: Sprint Run (Process and Product), Jump (Process), Side Gallop, Hop, Kick, Overarm Throw, Catch, Balance.  Skills not retained in final model for Body Fat %: Sprint Run (Process and Product), Side Gallop, Hop, Kick, Overarm Throw, Catch  ***Females***  BMI 🡪Jump Height (Product)  β = -3.1***  BF% 🡪Jump Height (Product)  β = -3.2***  Skills not retained in final model for BMI and Body Fat %: Sprint Run (Process and Product), Jump (Process), Side Gallop, Hop, Kick, Overarm Throw, Catch, Balance. | MC was not a predictor of future weight status for girls.  For boys, catching was a predictor of only body fat percentage (but not BMI) one year later.  For boys, body fat percentage was the best predictor for jump (process) scores, as well as balance. BMI was a better predictor for jump height (product score).  For girls, weight status (BMI and body fat percentage) was most predictive of future jumping height one year later. |
| [61] Cheng et al. (2016) | Chile | N/A | 2 (5 years) | 668 (360 M, 308 F) | T1: 5.0  T2: 10.0 | BOTMP-SF  *Product* | **Total Scores**  T1: 52.4 (8.2)  T2: 45.0 (10.2) | Weight, Height, BMI (Kuczmarski et al. 2002) | **BMI z-Scores**  T1: 0.84 (1.0)  T2: 0.73 (1.0) | Cross lagged panel analysis | **MC (T1) 🡪WS (T2)**  β = -0.02  **WS (T1) 🡪 MC (T2)**  β = -0.16*** | A higher BMI at age five predicted lower MC at age 10.  Low MC at age five was not a predictor of BMI at age 10. |
| [64] Coppens et al. (2019) | Belgium | N/A | 3 (T1 to T2 = 1 year; T2 to T3 = 1 year) | 558 (293 M, 265 F) | 8.2 (1.1) | KTK  *Product* | **Total Scores**  ***Males***  T1: 166.1 (39.8)  T2: 196.2 (40.1)  T3: 224.9 (40.7)  ***Females***  T1: 162.9 (43.01)  T2: 191.5 (41.0)  T3: 217.0 (42.2)  ***Total***  T1: 164.6 (41.4)  T2: 194.0 (40.6)  T3: 221.1 (41.6) | Height, weight, BMI | **BMI**  ***Males***  T1: 16.2 (2.0)  ***Females***  T1: 16.3 (2.2)  ***Total***  T1: 16.3 (2.1) | Latent Growth Curve | **WS (T1) 🡪MC (T2)**  ß = -1.42** | Children with higher BMI at baseline had significantly worse MC two years later compared to those with a lower BMI. |
| [33] Dos Santos et al. (2018) | Portugal | N/A | 4 (T1 to T2 = 1 year  T2 to T3 = 1 year  T3 to T4 = 1 year) | 245 (123 M, 122 F) | T1 = 6  T2 = 7  T3 = 8  T4 = 9 | KTK  *Product* | **Total Score**  ***Males***  T1: 111.0 (31.1)  T2: 140.5 (36.6)  T3: 164.6 (37.0)  T4: 182.1 (39.8)  ***Females***  T1: 99.5 (27.2)  T2: 130.9 (30.7)  T3: 157.8 (34.2)  T4: 174 (38.6) | Weight | **Body Mass (kg)**  ***Males***  T1: 24.6 (5.2) T2: 26.9 (6.3) T3: 30.2 (7.5) T4: 34.2 (0.1)^1^  ***Females***  T1: 24.0 (4.6) T2: 26.1 (5.6) T3: 29.8 (6.6) T4: 33.5 (7.7) | Multilevel Modeling with Repeated Measures | **WS🡪MC**    ***Body Mass***  Estimate: -0.43 (SE = 0.06)*** | Children who were leaner had better gross MC across the three years relative to their peers. |
| [40] Henrique et al. (2018) | Portugal | N/A | 4 (1 year) | 245 (123 M, 122 F) | T1: 6.46– 9.46 | KTK  *Product* | **Walking backwards**  ***Males***  T1: 29.0 (13.8)  T2: 37.9 (13.9)  T3: 43.3 (13.7)  T4: 48.3 (13.0)  ***Females***  T1: 28.6 (15.5)  T2: 36.6 (14.7)  T3: 40.5 (11.5)  T4: 46.6 (13.7)  **Jumping sideways**  ***Males***  T1: 32.0 (9.8)  T2: 37.7 (11.0)  T3: 44.6 (13.9)  T4: 54.7 (13.2)  ***Females***  T1: 28.6 (8.9)  T2: 36.0 (9.8)  T3: 43.9 (13.6)  T4: 52.5 (12.0)  **Hopping for height**  ***Males***  T1: 18.6 (11.8)  T2: 27.5 (16.0)  T3: 38.0 (17.9)  T4: 43.5 (18.5)  ***Females***  T1: 15.3 (10.1)  T2: 23.0 (13.6)  T3: 35.3 (17.4)  T4: 38.4 (17.8)  **Moving sideways**  ***Males***  T1: 30.5 (5.0)  T2: 36.9 (6.5)  T3: 40.3 (5.5)  T4: 41.9 (7.6)  ***Females***  T1: 27.6 (5.2)  T2: 35.7 (6.5)  T3: 39.0 (7.2)  T4: 40.4 (6.9)  **Motor quotient**  ***Males***  T1: 94.9 (13.9)  T2: 98.8 (16.1)  T3: 97.0 (16.3)  T4: 88.0 (17.4)  ***Females***  T1: 81.7 (14.3)  T2: 89.5 (15.0)  T3: 92.9 (16.6)  T4: 79.2 (16.9) | Weight, Height, BMI (Cole, Freeman, & Preece, 1995).  Skinfold measurement (triceps and subscapular) Procedures of Lohman, Roche, and Martorell (1988).  Sum of measures used for proxy of subcutaneous fat | **BMI**  ***Upper canal***  T1: 16.6 (1.6)  ***Lower canal***  T1: 18.6 (3.4)  **Adiposity (sum of SF)**  ***Upper canal***  T1: 12.9 (3.5)  ***Lower canal***  T1: 23.4 (12.8) | T-test | **WS🡪MC**  ***BMI***  t = 3.39**  ***Adiposity***  t = 4.77** | Both BMI and adiposity were significantly different at age 6 among children classified in the upper and lower canals (i.e., high and low MC) which resulted in different trajectories of MC across a four-year period.  This favoured the high MC group who had a lower BMI and lower levels of adiposity. |
| [41] Herrmann, Heim, and Seelig (2017) | Germany | N/A | 2 (8 months) | 1031^2^ (557 M, 474 F)  436^3^ (209 M, 227 F) | *T1:* 6.8 (0.4)  *T2:* 7.5 (0.4) | MOBAK-1  *Product* | **Self-Moving**  T2: 5.7 (1.8)  **Object-Moving**  T2: 5.4 (1.9) | Height, Weight, BMI | **BMI**  ***All***  T1:16.2 (2.2)  T2:16.4 (2.5)  ***Males***  T1:16.2 (2.1)  T2: 16.6 (2.5)  ***Females***  T1: 16.2 (2.3)  T2: 16.3 (2.4) | Autoregressive Structural Equation Modelling | **WS🡪MC**  ***Self-Moving***  ß = -0.24**  ***Object Moving***  ß = -0.10** | Individuals with lower BMIs had higher skill levels (object control, locomotor, and balance/stability) one year later. |
| [62] Lima, Bugge, Ersbøll, Stodden, and Andersen (2019) | Denmark | N/A | 3 (T1 to T2 = 3 years  T2 to T3 = 4 years) | *T1:*696 (369 M, 327 F)  *T2:* 615 (323 M, 292 F)  *T3:* 442 (231 M, 211 F) | *T1:* 6.75 (0.4)  *T2:* 9.59 (1.1)  *T3:* 13.4 (0.3) | KTK  *Product* | **Total Scores**  ***Total***  T1: 119.2 (27.7)  T2: 195.2 (34.6)  T3: 249.4 (29.4)  ***Males***  T1: 120.1 (28.4)  T2: 194.8 (34.9)  T3: 251.4 (29.9)  ***Females***  T1: 118.2 (26.8)  T2: 195.6 (34.4)  T3: 247.3 (28.8) | Sum of four skinfolds (bicipital, tricipital, subscapular, and suprailiac) | **Skinfolds**  ***Total***  T1: 26.6 (10.0)  T2: 33.6 (16.5)  T3: 34.9 (16.8)  ***Males***  T1: 24.4 (9.0)  T2: 30.1 (14.5)  T3: 31.6 (17.0)  ***Females***  T1: 29.2 (10.4)  T2: 37.4 (17.6)  T3: 38.5 (15.9) | Multilevel Linear Regressions | **MC 🡪WS**  ***Males***  −0.31 Z-scores, 95% CI: −0.36 to −0.26.  ***Females***  −0.26 Z-scores, 95% CI: −0.31 to −0.20).  **WS🡪MC**  ***Males***  −0.45 Z-scores, 95%CI: −0.52 to −0.38.  ***Females***  −0.35 Z-scores, 95% CI: −0.42 to −0.28). | MC and sum of skinfolds demonstrated a reciprocal influence on each other’s development across time.  For boys, the strength of the association between MC and Weight Status increased across 3 and 7 years follow-up, independent of the direction analysed. For girls, an increase in strength of association was observed from 6 to 9 years-of-age, and maintenance of the strength of the association from 9 to 13 years-of-age, independent of direction analysed.  Sum of skinfolds had a stronger influence on the development of MC for both boys and girls, than MC had on the sum of skinfolds. |
| [63] Lima, Bugge, Pfeiffer, and Andersen (2017) | Denmark | N/A | 3 (T1 to T2 = 3 years; T2 to T3 = 4 years) | T1: 649; 337 M, 304 F  T2: 316 M, 289 F  T3: 222 M, 205 F | T1: 6.8 (0.4)  T2: 9.6 (1.1)  T3: 13.4 (0.3) | KTK  *Product* | **Motor Quotient Score**  ***All***  T1: 98.1 (14.5)  T2: 96.3 (14.4)  T3: 97.3 (16.4) | Weight, Height, BMI | **BMI**  ***All***  T1: 16.0 (1.8)  T2: 17.3 (2.4)  T3: 19.2 (2.7) | Mixed-effects logistic regression analysis | **WS🡪MC**  ***BMI***  **High MC**  OR = 1.0  **Medium MC**  OR = 1.72, 95% CI [0.99, 2.99]  **Low MC**  OR = 5.44, 95% CI [3.0, 9.87] | Children with a higher BMI at age 6 were more than 5 times more likely to be in the low MC group after 7 years compared to their lower BMI peers. |
| [65] Lima, Pfeiffer et al., 2017 | Denmark | N/A | 3 (T1 to T2 = 3 years; T2 to T3 = 4 years) | T1: 696 T2: 617 T3: 513 | T1: 6.8 (0.4)  T2: 9.6 (1.1)  T3: 13.4 (0.3) | KTK  *Product* | **Motor Quotient Score**  ***All***  T1: 119.2 (27.7) T2: 195.2 (34.6)  T3: 249.4 (29.4)  ***Males***  T1: 120.1 (28.4) T2: 194.8 (34.9) T3: 251.4 (29.9)  ***Females***  T1: 118.2 (26.8) T2: 195.6 (34.4) T3: 247.3 (28.8) | Sum of four skinfolds (Biceps, triceps, subscapular, and suprailiac) | **Body fatness (mm)**  ***All***  T1: 26.6 (10.0)  T2: 33.5 (16.5)  T3: 34.9 (16.8)  ***Males***  T1: 24.4 (9.0) T2: 30.1 (14.5)  T3: 31.6 (17.0)  ***Females***  T1: 29.2 (10.4)  T2: 37.4 (17.6)  T3: 38.5 (15.9) | SEM | **MC🡪WS**  β= −0.23* (95% CI: −0.287, −0.173) | Across a seven year period, children who had higher levels of gross MC at baseline were more likely to have a healthier body fatness level (assessed by skinfolds) than their peers with lower gross MC. |
| [53] Wagner, Jekauc, Worth, and Woll (2016) | Germany | N/A | 2 (6 years) | 940 (462 M, 478 F) | *T1:* 8.1 (1.5)  *T2:* 14.4 (1.5) | MoMo test battery items (backwards walk, side to side jumping, one leg balance)  *Product* | NR; groups stratified according to MC level | Height, weight, BMI (Kromeyer-Hauschild et al., 2011) | NR | Binary logistic regression | **MC (T1) 🡪WS (T2)**  B = 0.58*  OR = 1.78 | Having low MC in childhood show a 1.78 times higher risk of an elevated BMI in adolescence, compared to children without poor MC. |
| ***Intervention Studies*** | | | | | | | | | | | | |
| [60] McGrane, Belton, Fairclough, Powell, and Issartel (2018) | Ireland | *Dose:* 9 month duration. Dose in lessons unclear  *Framework/Theory:* NR  *Approach:* School-based intervention with four components: (1) health-related activity and FMS in PE, (2) Parents and guardians educated about health benefits of PA,  (3) 2 teacher/ staff workshops with the main objective to promote PA participation among staff and students during school time, and (4) website | 3 (T1 to T2 = 8 months,  T2 to T3 = 4 months) | 482 (246 M, 236 F) | 12.8 (0.4) | TGMD-2  TGMD (skip, vertical jump)  Victorian Fundamental Movement Skills Manual (balance)  *Process* | **Object Control**  ***Intervention***  T1: 36.7 (4.4)  T2: 38.7 (7.0)  T3: 42.5 (4.5)  ***Control***  T1: 37.4 (4.1)  T2: 36.1 (6.4)  T3: 40.1 (5.4)  **Locomotor**  ***Intervention***  T1: 52.1 (5.9)  T2: 50.3 (14.8)  T3: 57.1 (7.4)  ***Control***  T1: 51.5 (5.7)  T2: 48.7 (12.9)  T3: 54.5 (7.6)  **Total FMS**  ***Intervention***  T1: 95.1 (8.4)  T2: 86.8 (6.0)  T3: 99.6 (11.7)  ***Control***  T1: 94.7 (8.5)  T2: 83.9 (22.1)  T3: 94.5 (12.0) | Weight, Height, BMI (Cole, Bellizzi, Flegal, & Dietz, 2000) | **BMI**  ***Intervention***  T1: 20.4 (3.3)  ***Control***  T1: 19.8 (3.0) | Multilevel linear regressions | **WS🡪MC**  ***Locomotor***  ***Normal Weight***  β  **=** 1.65*, 95% CI [0.75, 2.55]  ***Overweight/Obese***  β  **=** 2.25***, 95% CI [1.35, 3.15]  **Object Control**  ***Normal Weight***  β  **=** 2.41***, 95% CI [1.16, 3.66]  ***Overweight/Obese***  β  **=** 1.95***, 95% CI [1.04, 2.85]  **Total FMS**  ***Normal Weight***  β  **=** 4.07***, 95% CI [1.62, 6.52]  ***Overweight/Obese***  β  **=** 4.04***, 95% CI [2.33, 5.75] | Intervention had positive and significant effects improving MC regardless of weight status (i.e., normal weight or overweight/obese at baseline) implying weight status didn’t have an impact on MC changes |
| * Reported within article, p < 0.05  ** Reported within article, p < 0.01  *** Reported within article, p < 0.001  N/A = Not applicable  ^1^ = Appears to be an error in reporting in the article as does not fit with other values  ^2^ = Participants with full motor competence data (Herrmann et al.,)  ^3^ = Participants with full motor competence data and sport participation data (i.e., physical activity) (Herrmann et al.)  BMI = Body mass index  BOT-MP SF = Bruininks-Oseretsky Test of Motor Proficiency, Short form  F = females  FMS = Fundamental motor skill  KTK = Körperkoordinationstest Für Kinder  M = Male  MC = Motor competence  PA = Physical activity  PE = Physical education  SD = Standard deviation  WS = Weight status | | | | | | | | | | | | |
