# Supplementary material for: Through the Looking Glass: A Systematic Review of Longitudinal Evidence, Providing New Insight for Motor Competence and Health
Source: Sports Med. 2021 Aug 31;52(4):875–920. doi: 10.1007/s40279-021-01516-8 (PMC8938405; doi:10.1007/s40279-021-01516-8)
Supplement: Supplementary file 4 — Supplementary file4 (DOCX 22 kb) [file 40279_2021_1516_MOESM4_ESM.docx]

| **Supplementary Table 4. Motor Competence and Perceived Movement Competence Results** | | | | | | | | | | | | |
| --- | --- | --- | --- | --- | --- | --- | --- | --- | --- | --- | --- | --- |
| ***Longitudinal Studies*** | | | | | | | | | | | | |
| **Study** | **Country** | **Intervention Description** | **Timepoints # (Duration)** | **Sample #**  ***(M, F)*** | **Age (SD)** | **MC Measure** | **MC Scores at each timepoint**  ***Mean (SD)*** | **PMC Measure**  ***Aligned with MC measure?*** | **PMC Scores at each timepoint**  ***Mean (SD)*** | **Analysis** | **Pathway tested and values** | **Overall findings** |
| [44] Lloyd, Saunders, Bremer, and Tremblay (2014) | Canada | Not applicable | 4  (T1 to T2 = 5 years; T2 to T3 = 5 years; T3 to T4 = 10 years) | *T1:* 17 (5 M, 12 F)  *T2:* 10 (4 M, 6 F)  *T3:* 13 (4 M, 9 F)  *T4:* 17 (5 M, 12 F) | *T1:* 6.8 (0.4) *T2:* 11.9 (0.4) *T3:* 16.8 (0.3) *T4:* 26.8 (0.4) | TGMD (T1 and T2 only)  *Process* | **Total Score**  ***Low Motor Proficiency***  T1: 26.33(2.34)  T2: 36.50(2.12)  ***High Motor Proficiency***  T1: 38.18 (2.56)  T2: 40.75 (2.96)  **Locomotor**  ***Low Motor Proficiency***  T1: 18.50 (3.21)  T2: 24.00 (1.41)  ***High Motor Proficiency***  T1: 22.91 (2.74)  T2: 24.63 (2.30)  **Object Control**  ***Low Motor Proficiency***  T1: 9.50 (1.52)  T2:12.50 (0.71)  ***High Motor Proficiency***  T1: 15.27 (2.65)  T2: 26.23 (2.48) | DCDQ teen recall (T4 recalling T3) and adult (T4 only; unpublished)  *Not aligned* | **DCDQ teen recall**  ***Low Motor Proficiency***  T3: 79.20 (7.5)  ***High Motor Proficiency***  T3: 91.80 (8.5)  **DCDQ-A**  ***Low Motor Proficiency***  T4: 89.60 (7.1)  ***High Motor Proficiency***  T4: 93.60 (4.9) | Correlation | **MC (T1) 🡪 PMC (T3)**  **Total TGMD – DCDQ Teen recall**  r = 0.65**  **Locomotor – DCDQ Teen recall**  r = 0.22  **Object Control – DCDQ Teen recall**  r = 0.59*  **MC (T1) 🡪 PMC (T4)**  **Total TGMD – DCDQ-A**  r = .35  **Locomotor – DCDQ-A**  r = 0.21  **Object Control – DCDQ-A**  r = 0.26 | Total and object control skills (not locomotor) at age 6 were significant and positively associated with perceived motor competence as a teenager (i.e., 10 years later).  MC at age 6 was not associated with perceptions of competence as an adult. |
| ***Experimental Studies*** | | | | | | | | | | | | |
| [66] Lander, Mergen, Morgan, Salmon, and Barnett (2019) | Australia | *Dose:* 90 mins/wk x 12 weeks  *Theory/Framework:* Intervention components informed by: Self-determination theory, Achievement goal theory, Competence motivation theory, and TARGET framework  *Approach:*  Intervention group: Teacher training and 12-week intervention targeting perceived and actual motor competence using SAAFE teaching principles  Control group: Usual practice | 2 (12 weeks) | 171 (171 F) | 12.5 (0.3) | Victorian Fundamental Motor Skill Teachers’ Assessment  *Process* | Not reported | Pictorial Scale of Perceived Movement Skill Competence (Barnett et al., 2015)  *Aligned*  Physical Self-Perception Profile (Fox & Corbin, 1989)  *Not aligned* | **Locomotor Skills**  ***Control***  *T1:* 17.86 (3.16)  *T2:* 18.00 (3.00)  ***Intervention***  *T1:* 18.47 (2.54)  *T2:* 18.82 (2.54)  **Object Control Skills**  ***Control***  *T1:* 18.68 (3.25)  *T2:* 18.74 (3.07)  ***Intervention***  *T1:* 19.47 (2.70)  *T2:* 20.34 (2.02)  **Total Skill**  ***Control***  *T1:* 36.54 (5.61)  *T2:* 36.74 (5.28)  ***Intervention***  *T1:* 37.94 (4.61)  *T2:* 39.16 (3.80)  **Sports Competence**  ***Control***  *T1:* 16.09 (3.71)  *T2:* 16.31 (3.61)  ***Intervention***  *T1:*17.54 (3.64)  *T2:* 18.44 (3.03)  **Physical Condition**  ***Control***  *T1:* 16.76 (3.27)  *T2:* 16.76 (3.27)  ***Intervention***  *T1:*18.15 (3.43)  *T2:* 18.15 (3.43)  **Body Attractiveness**  ***Control***  *T1:* 14.63 (3.54)  *T2:* 14.64 (3.52)  ***Intervention***  *T1:* 16.23 (3.29)  *T2:* 16.23 (3.29)  **Strength**  ***Control***  *T1:* 16.15 (3.13)  *T2:* 16.13(3.15)  ***Intervention***  *T1:* 16.34 (3.30)  *T2:* 16.32 (3.29)  **Physical Self-Worth**  ***Control***  *T1:* 16.78 (3.46)  *T2:* 16.76 (3.45)  ***Intervention***  *T1:* 17.77 (3.56)  *T2:* 18.23 (3.00)  **Total Physical Self-Perception**  ***Control***  *T1:* 80.41 (14.16)  *T2:* 79.99 (13.21)  ***Intervention***  *T1:* 86.04 (14.00)  *T2:* 86.52 (11.78) | Linear Mixed Models | **MC 🡪 PMC**  **Perceived object control skill**  t (168) = 9.30***  B = 0.94  **Perceived locomotor skill**  t(168) = 3.02**  B = 0.27  **Perceived total skill**  t(168) = 8.43***  B = 1.20  **Physical self-perception** t(168) = 7.10***  B = 1.54  No association between change in actual MC caused by the intervention, and post-intervention perceptions. This interaction term was excluded from the models. | No effect from the intervention regarding change in perceived motor competence as a result of the change in actual motor competence and. |
| [67] Marouli, Papavasileiou, Dania, and Venetsanou (2016) | Greece | *Dose:* 40 min sessions/ twice wk x 8 weeks  *Theory/Framework:*  *Approach:*  Intervention:  Psychomotor Education pedagogical approaches and principles of the Orff-Schulwerk method of rhythmic education.  Control group: Usual school curriculum | 2 (8 weeks) | 29 (16 M, 13 F) | 4.1 (0.5) | BOT-2 short form  *Product* | **Total Score**  ***Control***  *T1:* 26.20 (9.77)  *T2:* 27.47 (10.42)  ***Intervention*** *T1:* 22.71 (8.96)  *T2:* 28.21 (9.92)  ***Total* *Sample***  *T1:* 24.52 (9.39)  *T2:* 27.83 (10.00) | Pictorial Scale of Perceived Competence and Social Acceptance for Young Children-Greek Version  Physical Competence and Peer Acceptance subscales only (Makri-Botsari, 2001)  *Not aligned* | **Mean Score**  ***Control***  *T1:* 2.83 (0.65)  *T2:* 2.97 (0.67)  ***Experimental*** *T1:* 2.53 (0.57)  *T2:* 2.84 (0.64)  ***Total***  *T1:* 2.68 (0.62)  *T2:* 2.91 (0.65) | Analysis of variance | **MC🡪PMC**  “Group” by “Measure” interaction F_1,27_= .58, not significant. Note. Interpreting this as interaction between (Time) pre-post/ (Group) Exp-Con – but with the wording of “measure” (instead of time).  “Group” main effect F_1,27_= 1.06, not significant  “Measure” main effect  F_1,27_= 4.35, not significant | No statistically meaningful change in PMC as a result of this MC intervention. |
| * Reported within article, p < 0.05  ** Reported within article, p < 0.01  *** Reported within article, p < 0.001  Note.  BOT = Bruininks-Oseretsky Test  DCDQ = Developmental coordination disorder questionnaire  DCDQ-A = Developmental coordination disorder questionnaire for adolescents  F = Female  M = Male  MC = Motor competence  PMC = Perceived movement competence  SD = Standard deviation  TGMD = Test of Gross Motor Development | | | | | | | | | | | | |
