# Supplementary material for: Through the Looking Glass: A Systematic Review of Longitudinal Evidence, Providing New Insight for Motor Competence and Health
Source: Sports Med. 2021 Aug 31;52(4):875–920. doi: 10.1007/s40279-021-01516-8 (PMC8938405; doi:10.1007/s40279-021-01516-8)
Supplement: Supplementary file 5 — Supplementary file5 (DOCX 37 kb) [file 40279_2021_1516_MOESM5_ESM.docx]

| **Supplementary Table 5. Perceived Motor Competence Mediating the Motor Competence and Physical Activity Relationship Results** | | | | | | | | | | | | | |
| --- | --- | --- | --- | --- | --- | --- | --- | --- | --- | --- | --- | --- | --- |
| ***Mediation Studies*** | | | | | | | | | | | | | |
| **Study** | **Country** | **Timepoints # (Duration)** | **Sample #**  ***(M, F)*** | **Age (SD)** | **MC measure** | **MC scores at each timepoint**  ***Mean (SD)*** | **PMC measure** | **PMC scores at each timepoint**  ***Mean (SD)*** | **PA measure**  ***Objective/ Subjective***  **Duration of measurement** | **PA scores at each timepoint**  ***Mean (SD)*** | **Analysis** | **Pathway tested and values** | **Overall findings** |
| [34] Britton, Belton, and Issartel (2019) | Ireland | 2 (1 year) | 224 (110 M, 114 F) | 12.3 (0.0) | Test of Gross Motor Development-3rd edition (kick, catch, overhand throw, one- and two-hand strike, run, skip, horizontal jump) *Process*  Victoria Department of Education Training Manual (vertical jump)  *Process*  Motor Assessment Battery for Children-2^nd^ edition (two-board balance, zigzag hop, walking toe to heel backward)  *Product* | **Locomotor skills**  ***Males***  *T1:* 29.6 (3.5)  *T2:* 31.1 (2.8)  ***Females*** *T1:* 29.2 (3.6)  *T2:* 29.5 (3.3)  **Object control skills**    ***Males***  *T1:* 33.2 (4.2)  *T2:* 36.4 (2.3)    ***Females*** *T1:* 26.1 (7.0)  *T2:* 30.5 (4.6)  **Balance/Stability**  ***Males***  *T1:* 36.6 (8.1)  *T2:* 38.6 (6.7)    ***Females***  *T1:* 36.4 (8.7)  *T2:* 39.2 (6.9) | Perceived athletic subscale of the Self-Perception Profile for Adolescents (Harter, 2012)  *Not aligned* | **Male**  *T1:* 3.0 (.6)  *T2:* 3.0 (0.7)  **Female**  *T1:* 2.8 (0.8)  *T2:* 2.5 (0.8) | Accelerometer (GT1M, GT3X, GT3X+, wGT3X-BT); MVPA mins  *Objective,* 7 days during waking hours | **MVPA Minutes**  ***Male***  *T1:* 54.4 (26.3)  *T2:* 44.2 (19.2)    ***Females*** *T1:* 46.3 (16.0)  *T2:* 37.1 (11.7) | SEM | **Pathway tested: PA (T1)🡪PMC (T1) 🡪MC (T2)**  ***PA (T1)🡪PMC (T1)***  r = 0.345***  ***PMC (T1)🡪 Locomotorskills (T2)***  r = 0.344***  PMC not significantly correlated with balance/stability or object control skills  **PA (T1)🡪MC (T2)**  Not Significant  **Pathway tested: MC (T1)🡪PMC (T1)🡪PA (T2)**  ***Object control skills (T1)🡪PMC (T1)***  r = 0.177***  ***Locomotor skills (T1)🡪PMC (T1)***  r = 0.247***  Other pathways not significant | Perceived athletic competence did not mediate the relationships between MC and PA overall.  Perceived athletic competence did unidirectionally mediate the relationship from physical activity to locomotor skill competence. |
| [72] Burns and Fu (2018) | USA | 1 | 84 (44 M, 40 F) | 11.6 (0.6) | Test of Gross Motor Development-3^rd^ edition  *Process* | **Locomotor Skills**  ***Males***  39.0 (6.3)  ***Females***  36.1 (5.8)  ***Total***  37.9 (6.1)  **Ball Skills**  ***Males***  48.9 (6.2)  ***Females***  39.4 (5.2)  ***Total***  41.2 (7.2)  **Total Scores**  ***Males***  88.1 (10.9)  ***Females***  74.9 (7.8)    ***Total***  80.5 (10.9) | Perceived Competence Scale for Children (Harter, 1978)  *Not aligned* | **Males**  3.4 (0.6)  **Females**  3.0 (0.7)  **Total**  3.3 (0.7) | Pedometer  (Yamax Digi Walker CW600); steps  *Objective,* 5 school days (8am-3pm) | **School Day Steps**  ***Males***  4214 (1435)  ***Females***  3376 (1579)  ***Total***  3681 (1577) | SEM | **PA🡪PMC🡪MC (total scores)**  ***PA🡪PMC***  B = 0.0012*  β = 0.27*  ***PMC🡪MC***  B = 4.81*  β = 0.30*  ***PA🡪MC***  B = 0.009  β = 0.13 | Perceived competence mediated the relationship between physical activity and motor competence |
| [68] Chan, Ha, Ng, and Lubans (2019) | China | 1 | 763 (289 M, 474 F) | 9.3 (1.7) | Test of Gross Motor Development-2^nd^ edition *Process* | **Locomotor**  38.8 (5.8)  **Object Control** 37.3 (7.1) | **Perceived Physical Competence**  Physical Competence subscale of the  Pictorial Scale of Perceived Competence and Social Acceptance for First-Second Grades (Harter and Pike, 1984)  *Not aligned*  Athletic Competence subscale of the Self-Perception Profile for Children (Harter, 1985)  *Not aligned*  **Perceived Movement Skill Competence**  Modified Self-Perception Profile for Children scale (Jones et al., 2010; Southall et al., 2004)  *Aligned* | **Perceived Physical Competence**  2.96 (0.63)  **Perceived Athletic Competence**  2.98 (0.53) | Accelerometer (ActiGraph GT3X+); MVPA  The Physical Activity Questionnaire for Older Children (Kowalski, Crocker, & Donen, 2004)  *Objective,* 7 days | **Accelerometer (MVPA minutes)**  40.86 (14.70)  **Self-Report (level of PA on 1-5 scale, low to very high PA, respectively)**  2.67 (0.70) | SEM | **MC🡪Perceived Physical Competence🡪Self-Report PA**  ***Locomotor MC🡪PMC***  β = 0.16** [.08, .25]  ***Object Control MC🡪PMC***  β = -0.04  ***PMC 🡪 Self Report PA***  β = 0.29  **MC🡪Perceived Movement Skill Competence🡪MVPA (accelerometer)**  ***Locomotor MC🡪PMC***  β = 0.11**  [.001, .22]  ***Object Control MC🡪PMC***  β = -0.30  ***PMC 🡪 MVPA***  β = 0.59* [.04, 1.14] | Perceived physical competence and enjoyment mediated the relationship between locomotor skills and self-reported physical activity, but not accelerometer assessed physical activity.  Perceptions of competence were not a mediator in the relationships between object control skills and physical activity (self-report or accelerometer). |
| [73] Crane, Naylor, Cook, and Temple (2015) | Canada | 1 | 116 (67 M, 49 F) | 5.6 (Not reported) | Test of Gross Motor Development-2^nd^ edition  *Process* | **Locomotor Skills**  26.3 (7.0)  **Object Control Skills**  20.9 (6.7) | Pictorial Scale of Perceived Competence and Social Acceptance for Young Children  (Harter and Pike, 1984)  *Not aligned* | **Total Score**  18.8 (3.0) | Accelerometer (ActiGraph GT1M); MVPA  *Objective,* 7 days during waking hours | **MVPA Minutes**  135.3 (29.9) | Regression | **MC🡪PMC🡪PA**  ***Object control MC🡪 MVPA***  B = 1.332**  ***Object Control MC🡪PMC***  B = 0.081*  **PMC🡪MVPA**  B = -0.194  **PA🡪PMC🡪MC**  ***PA🡪 Object Control MC***  B = 0.068***  ***PA🡪PMC***  B = 0.003  ***PMC🡪MC***  Not assessed  Locomotor skills had a non-significant association with MVPA and therefore was excluded from mediation analyses | Perceived competence did not mediate the relationship between motor competence and physical activity in either direction |
| [70] Fu and Burns (2018) | USA | 1 | 66 (30 M, 36 F) | 11.6 (0.5) | Test of Gross Motor Development-3^rd^ edition  *Process* | **Total Scores**  ***Males***  86.1(12.1)  ***Females*** 75.5 (7.6)  ***Total***  80.2 (11.1) | Perceived Competence Scale for Children  (Harter, 1978)  *Not aligned* | **Total Score**  ***Males***  3.3 (0.6)  ***Females***  3.0 (0.7)  ***Total***  3.1 (0.7) | Pedometer (Yamax Digi Walker CW600); steps  *Objective,* 5 school days (8am-3pm) | **Steps Per Minute**  ***Males***  10.4 (2.6)  ***Females***  9.1 (3.1)  ***Total***  9.7 (3.0) | Causal mediation analysis | **MC🡪PMC🡪PA**  ***MC🡪PMC***  β = 0.02*  r = 0.37*  ***PMC🡪PA***  β = 0.88*  r = 0.25*  ***MC🡪PA***  r = 0.33*  **Total Effect**  0.070*  **Average Direct Effect**  0.048  **Average Causal Mediation Effect**  0.022* | Perceived competence mediates the relationship between MC and school day physical activity as assessed by pedometer steps |
| [71] Gu, Thomas, and Chen (2017) | USA | 1 | 262 (133 M, 129 F) | 10.9 (0.8) | Physical Education Metrics (soccer and gymnastic sequences) *Process* | **Total Scores** 12.6 (3.9)  **Soccer skills**  5.8 (2.6)  **Gymnastics skills**  6.7 (2.4) | Five item questionnaire developed by the author (Xiang et al., 2004)  *Not aligned* | **Mean Score**  ***High MC Group***  4.3 (0.5)  ***Low MC Group***  3.9 (0.6) | Godin Leisure-Time Exercise Questionnaire (Godin * Shephard, 1997); Number of 30 min activities  *Subjective,* 7 day recall  Pedometer (ACCUSPLIT); steps  *Objective,*6 physical education lessons (50 minutes) | **Self-Report Minutes**  ***High MC Group***  79.8 (36.5)  ***Low MC Group***  71.0 (34.98)  **Pedometer Steps**  ***High MC Group***  2083 (432.5)  ***Low MC Group***  1859 (693.5) | Structural Equation Modelling  PA was a latent variable which included objective and subjective PA assessment | **MC🡪PMC🡪PA**  **MC🡪PA**  β = 0.09  **MC🡪 PMC**  β = 0.45**  **PMC 🡪 PA**  β = 0.95** | Perceived competence fully mediated the relationship between MC and physical activity (latent variable: self-reported PA – leisure and pedometer-based PA during physical education class). |
| [74] Jaakkola et al. (2019) | Finland | 1 | 422 (176 M, 246 F) | 11.3 (0.3) | Leaping test, throwing-catching combination test, two-legged jumping from side to side  *Product* | **MC sum scores^1^**  ***Males***  0.15 (0.82)  ***Females***  0.06 (0.75)  **5‐leaps test**  ***Males***  7.8 (0.9)  ***Females***  7.8 (0.9)  **Throwing‐catching combination test**  ***Males***  12.5 (4.9)  ***Females***  9.9 (5.0)  **Two‐legged jumping from side to side test**  ***Males***  74.3 (12.9)  ***Females***  77.6 (12.5) | Finnish version of the sport competence dimension of the Physical Self‐Perception Profile  (Fox and Corbin, 1989)  *Not aligned* | **Mean Score**  ***Males***  2.4 (0.9)  ***Females***  2.6 (0.9) | Accelerometer (ActiGraph GT3X+); MVPA  *Objective,* 7 days during waking hours | **MVPA Minutes**  ***Males***  64.1 (24.7)  ***Females***  55.3 (21.0) | SEM | **MC 🡪 PMC 🡪 PA**  ***Females***  Standardized Estimate = .06 [0.03]**  Males had a significant direct path from MC 🡪 MVPA (β = 0.29), so mediation analysis could not be conducted  **MC🡪 PMC**  β = 0.41*  ***Females***  **PMC 🡪 MVPA** β = 0.16  **PA 🡪 PMC 🡪 MC**  ***Females***  Standardized estimate = .04 [.02]**  ***Males***  Standardized estimate = .06 [.02]**  **PA 🡪 PMC**  β = 0.25*  **PMC 🡪 MC**  β = .20*  **PA 🡪 MC** β = 0.15* | Perceived competence mediated the relationship from MC to MVPA, but only in girls.  Perceived competence was a mediator for girls and boys when MVPA predicted MC. |
| [38] Jekauc, Wagner, Herrmann, Hegazy, and Woll (2017) | Germany | 2 (6 years) | 698 (335 M, 363 F) | *T1:* 14.2 (2.0)  *T2:* 20.6 (2.0) | Jumping side-to-side, single leg balance, backward walking  *Product* | **Jumping side-to-side**  *T1:* 34.2 (6.2)  *T2:* 39.9 (6.8)    **Single leg balance** *T1:* 4.5 (5.4)  *T2:* 2.4 (3.8)  **Backward walking** *T1:* 34.8 (9.2)  *T2:* 39.2 (8.0) | Physical Self-Description Questionnaire (Stiller and Alfermann, 2007)  *Not aligned* | ***T2 Only***  17.8 (3.3) | MoMo Physical Activity Questionnaire; MVPA  *Subjective,* Sport club activity only | **MVPA Minutes**  *T1:* 110.0 (144.9)  *T2:* 71.6 (126.1) | Regression | **MC (T1)🡪PMC(T2)🡪PA (T2)**  **MC (T1)🡪PMC (T2)**  β = 0.261***  **PMC (T2)🡪PA (T2)**  β = 0.178***  **MC (T1)🡪PA (T2)**  β = -0.021  **Indirect effect**  β = 0.046  **PA (T1)🡪PMC (T2)🡪MC (T2)**  **PA (T1)🡪PMC (T2)**  β = 0.212***  **PMC (T2)🡪 MC (T2)**  β = 0.114**  **PA (T1)🡪MC (T2)**  β = 0.065 | Physical self-concept mediates the relationship between motor competence and sport club physical activity in both directions. |
| [69] Khodaverdi, Bahram, Stodden, and Kazemnejad (2016) | Iran | 1 | 352 (352 F) | 8.8 (0.3) | Test of Gross Motor Development-2^nd^ edition  *Process* | **Total Scores**  76.3 (9.3)  **Locomotor skills**  41.9 (6.6)  **Object control skills**  34.3 (5.5) | Physical ability subscale of the Self-Description Questionnaire -1 (Bahram & Shafizade, unpublished)  *Not aligned* | **Total Score**  34.8 (4.4) | Physical Activity Questionnaire for Older Children (Faghihimani et al., 2010); scale of 1-5 (1 indicates low PA)  *Subjective,* 7 day recall of MVPA | 3.3 (0.9) | Regressions | **MC (locomotor) 🡪PMC🡪PA**  **Direct effect**  b = 0.32***  **Indirect effect**  b = 0.16^2^  [.12, .32]  **MC 🡪 PMC**  b = .22**  **PMC 🡪 PA**  b = .28 ***  Object control skills were not tested for mediation as correlations were not significant with PA | Perceived competence mediated the relationship between locomotor skill competence and self-reported physical activity in 8-9 year old Iranian girls.  Object control skills were not eligible to be tested for mediation. |
| * Reported within article, p < 0.05  ** Reported within article, p < 0.01  *** Reported within article, p <0.001  ^1^ = Z-Scores reported  ^2^ = Significant, but level of significance not reported  F = Female  M = Male  MC = Motor Competence  MVPA = Moderate-to-Vigorous Physical Activity  PA = Physical activity  PMC = Perceived Movement Competence  VPA = Vigorous physical activity | | | | | | | | | | | | | |
