# Supplementary material for: Through the Looking Glass: A Systematic Review of Longitudinal Evidence, Providing New Insight for Motor Competence and Health
Source: Sports Med. 2021 Aug 31;52(4):875–920. doi: 10.1007/s40279-021-01516-8 (PMC8938405; doi:10.1007/s40279-021-01516-8)
Supplement: Supplementary file 6 — Supplementary file6 (DOCX 44 kb) [file 40279_2021_1516_MOESM6_ESM.docx]

| **Supplementary Table 6. Motor Competence (MC) and Health-Related Fitness (HRF) Results** | | | | | | | | | | | | | | | | | | |
| --- | --- | --- | --- | --- | --- | --- | --- | --- | --- | --- | --- | --- | --- | --- | --- | --- | --- | --- |
| ***Longitudinal Studies*** | | | | | | | | | | | | | | | | | | |
| **Study** | **Country** | | **Intervention description** | **Timepoints # (Duration)** | **Sample #**  ***(M, F)*** | **Age (SD)** | **MC measure** | **MC scores at each timepoint**  ***Mean (SD)*** | **Fitness measure** | **Fitness scores at each timepoint**  ***M (SD)*** | **Analysis** | | **Initial analyses (e.g., correlations)** | | **Pathway tested and values** | | **Overall findings** | |
| [42] Antunes et al., 2015 | Portugal | | N/A | 2 (6 years) | 158 (83 M, 75 F) | T1:  Group 1: 6  Group 2: 7  Group 3: 8  T2:  Group 1: 12  Group 2: 13  Group 3: 14 | KTK  *Product*  TGMD-2  *Process* (not used as a measure of MC in this study, noted as FMS) | **Walking backwards**  ***Males***  T1  Group 1: 40.0(11.3)  Group 2: 44.5(11.1)  Group 3: 49.1(12.1)  T2  Group 1: 59.9(12.1)  Group 2: 55.8(13.0)  Group 3: 64.2 (9.4)  ***Females***  T1  Group 1: 34.3(12.3)  Group 2: 44.0(10.4)  Group 3: 46.3(12.7)  T2  Group 1: 49.0(16.9)  Group 2: 56.9(9.6)  Group 3: 56.5(13.8)  **Hopping**  ***Males***  T1  Group 1: 21.4(10.2)  Group 2: 29.4(12.4)  Group 3: 35.2(7.7)  T2  Group 1: 61.1(14.4)  Group 2: 60.9(16.0)  Group 3: 70.0 (9.6)  ***Females***  T1  Group 1: 19.4(11.1)  Group 2: 29.3(8.8)  Group 3: 33.2(13.9)  T2  Group 1: 51.7(12.9)  Group 2: 54.8(10.6)  Group 3: 52.4(11.7)  **Jumping sideways**  ***Males***  T1  Group 1: 31.0(8.8)  Group 2: 34.3(7.8)  Group 3: 42.1(7.3)  T2  Group 1: 70.8(11.4)  Group 2: 68.7(15.0) Group 3: 78.1(7.3)    ***Females***  T1  Group 1: 31.3(7.2)  Group 2: 41.7(13.1)  Group 3: 42.6(12.2)  T2  Group 1: 64.2(14.6)  Group 2: 67.6(11.2)  Group 3: 69.7(11.6)  **Moving sideways**  ***Males***  T1  Group 1: 30.7(4.8)  Group 2: 34.1(4.7)  Group 3: 37.4(4.2)  T2  Group 1: 50.0 (6.4)  Group 2: 49.1 (7.5)  Group 3: 54.2 (5.2)  ***Females***  T1  Group 1: 28.5(5.5)  Group 2: 32.9(4.9)  Group 3: 34.5(5.5)  T2  Group 1: 45.7(10.5)  Group 2: 50.4 (7.1)  Group 3: 50.3 (6.7) | Eurofit test battery (Adam et al., 1988)  *Males*  flexed arm hang, handgrip,  sit-ups, standing long jump  Females:  sit and reach, standing long jump | **Flexed arm hang**  **(T1)**  ***Males***  Group 1: 5.1 (6.6)  Group 2: 4.8 (4.0)  Group 3: 5.7 (7.0)  **Handgrip (T1)**  ***Males***  Group 1: 10.1 (2.3)  Group 2: 12.4 (2.1)  Group 3: 13.8 (3.4)  **Sit ups**  **(T1)**  ***Males***  Group 1: 15.0 (4.8)  Group 2: 16.9 (4.1)  Group 3: 16.2 (4.0)  **(T2)**  Group 1: 22.4(5.1)  Group 2: 23.1(4.7)  Group 3: 24.1(3.7)  **Standing long jump**  **(T1)**  ***Males***  Group 1: 111.1 (14.9)  Group 2: 116.8 (16.5)  Group 3: 120.3 (12.9)  **(T2)**  Group 1: 157.6(22.5)  Group 2: 159.31(20.9)  Group 3: 180.1(21.0)  ***Females***  **(T1)**  Group 1: 96.9 (16.8)  Group 2: 105.2 (15.6)  Group 3: 109.1 (15.0)  (T2)  Group 1: 138.6(21.6)  Group 2: 146.3(18.3)  Group 3: 141.6(24.7)  **Sit and reach**  ***Females***  **(T1)**  Group 1: 21.3 (4.7)  Group 2: 22.1 (4.8)  Group 3: 18.6 (5.7)  **(T2)**  Group 1: 24.1(8.6)  Group 2: 24.4(5.5)  Group 3: 24.2(8.1) | Stepwise multiple linear regression | |  | | **HRF (T1) 🡪 MC (T2)^1^**  **Walking Backwards and Flexed Arm Hang**  ***Males Group 1***  No result reported  ***Males Group 2***  No result reported  ***Males Group 3***  No result reported  **Walking Backwards and Handgrip**  ***Males Group 1***  No result reported  ***Males Group 2***  No result reported  ***Males Group 3***  ß = -0.30*  **Walking Backwards and Sit-Ups**  ***Males Group 1***  No result reported  ***Males Group 2***  No result reported  ***Males Group 3***  No result reported  **Walking Backwards and Standing Long**  **Jump**  ***Males Group 1***  No result reported  ***Males Group 2***  No result reported  ***Males Group 3***  No result reported  ***Females Group 1***  No result reported  ***Females Group 2***  No result reported  ***Females Group 3***  No result reported  **Hopping and Flexed Arm Hang**  ***Males Group 1***  No result reported  ***Males Group 2***  ß = 0.31*  ***Males Group 3***  No result reported  **Hopping and Handgrip**  ***Males Group 1***  No result reported  ***Males Group 2***  No result reported  ***Males Group 3***  No result reported  **Hopping and Sit-Ups**  ***Males Group 1***  No result reported  ***Males Group 2***  No result reported  ***Males Group 3***  No result reported  **Hopping and Standing Long Jump**  ***Males Group 1***  No result reported  ***Males Group 2***  ß = 0.29*  ***Males Group 3***  No result reported  ***Females Group 1***  No result reported  ***Females Group 2***  No result reported  ***Females Group 3***  No result reported  **Jumping Sideways**  **and Flexed Arm Hang**  ***Males Group 1***  No result reported  ***Males Group 2***  ß = 0.44*  ***Males Group 3***  No result reported  **Jumping Sideways**  **and Handgrip**  ***Males Group 1***  No result reported  ***Males Group 2***  No result reported  ***Males Group 3***  No result reported  **Jumping Sideways**  **and Sit-Ups**  ***Males Group 1***  No result reported  ***Males Group 2***  ß = 0.53*  ***Males Group 3***  ß = -0.38*  **Jumping Sideways**  **and Standing Long Jump**  ***Males Group 1***  No result reported  ***Males Group 2***  No result reported  ***Males Group 3***  No result reported  ***Females Group 1***  No result reported  ***Females Group 2***  ß = 0.46*  ***Females Group 3***  No result reported  **Moving Sideways**  **and Flexed Arm Hang**  ***Males Group 1***  No result reported  ***Males Group 2***  No result reported  ***Males Group 3***  No result reported  **Moving Sideways**  **and Handgrip**  ***Males Group 1***  No result reported  ***Males Group 2***  No result reported  ***Males Group 3***  No result reported  **Moving Sideways**  **and Sit-Ups**  ***Males Group 1***  No result reported  ***Males Group 2***  No result reported  ***Males Group 3***  No result reported  **Moving Sideways**  **and Standing Long Jump**  ***Males Group 1***  No result reported  ***Males Group 2***  No result reported  ***Males Group 3***  No result reported  ***Females Group 1***  No result reported  ***Females Group 2***  No result reported  ***Females Group 3***  No result reported  **Walking Backwards and Sit and Reach**  ***Females Group 1***  No result reported  ***Females Group 2***  No result reported  ***Females Group 3***  No result reported  **Hopping and Sit and Reach**  ***Females Group 1***  No result reported  ***Females Group 2***  No result reported  ***Females Group 3***  ß= 0.42*  **Jumping Sideways**  **and Sit and Reach**  ***Females Group 1***  No result reported  ***Females Group 2***  No result reported  ***Females Group 3***  ß = 0.50*  **Moving Sideways**  **and Sit and Reach**  ***Females Group 1***  No result reported  ***Females Group 2***  No result reported  ***Females Group 3***  ß = 0.34* | | Childhood HRF is related to adolescent motor competence (operationalized in this study as motor coordination).  The longitudinal relationship between aspects of HRF and aspects of motor competence vary based on gender and age as well as in combination with FMS. | |
| [64] Coppens et al., 2019 | Belgium | | N/A | 3 (1 year) | 558 (293 M, 265 F) | 8.2 (1.1) | KTK  *Product* | **Total Scores**  ***Males***  T1: 166.1 (39.8)  T2: 196.2 (40.1)  T3: 224.9 (40.7)  ***Females***  T1: 162.9 (43.01)  T2: 191.5 (41.0)  T3: 217.0 (42.2)  ***Total***  T1: 164.6 (41.4)  T2: 194.0 (40.6)  T3: 221.1 (41.6) | Eurofit (T1 only; 20 meter shuttle run [cardiorespiratory endurance]; standing long jump [musculoskeletal fitness], sit-and-reach [flexibility]) | **20 Meter shuttle run**  ***Males***  T1: 4.9 (2.2)  ***Females***  T1: 3.6 (1.7)  ***Total***  T1: 4.2 (2.1)  **Standing long jump**  ***Males***  T1: 124.1 (20.5)  ***Females***  T1: 118.8 (20.6)  ***Total***  T1: 121.6 (20.7)  **Sit-and-reach**  ***Males***  T1: 19.5 (5.2)  ***Females***  T1: 22.6 (4.9)  ***Total***  T1: 21.0 (5.3) | Latent Growth Curve | |  | | **HRF (T1) 🡪MC (T3)**  **20m shuttle run**  ß = −0.49  **Standing long jump**  ß = −0.72  **Sit-and-reach**  ß = 0.34 | | Baseline HRF measures were not a significant predictor of change in motor competence across two years for boys or girls. | |
| [52] de Souza et al., 2014 | Portugal | | N/A | 2 (4 years) | 285 (143 M, 142 F) | T1: 6  T2: 10 | KTK  *Product* | ***Groups based on T2 HRF tertile (1 mile walk/run time)***  **Upper**  ***Males***  101.9 (27.6)  ***Females***  *97.3 (25.9)*  **Medium**  ***Males***  82.0 (21.1)  ***Females***  85.8 (26.2)  **Lower**  ***Males***  84.8 (25.9)  ***Females***  80.2 (27.6) | Health-Related Fitness: Fitnessgram (1 mile walk/run, curl-ups, trunk lifts, push-ups)  Performance-Related Fitness: American Alliance for Health, Physical Education, Recreation and Dance) AAHPERD Youth Fitness Test (standing long jump, grip strength) | T2 Fitness (1 mile walk/ run only) categorically defined as Upper, Medium, and Lower at T2. | ANOVA | |  | | **MC (T1) 🡪HRF (T2)**  **MC and fitness**  ***Males***  F = 7.21  ***Females***  F = 2.90  **Upper vs Medium Fitness**  ***Males***  p = 0.00**  ***Females***  p = 0.36  **Medium vs Lower Fitness**  ***Males***  p = 1.00  ***Females***  p = 0.88  **Upper vs Lower Fitness**  ***Males***  p = 0.02*  ***Females***  p = 0.06 | | Girls with upper and middle levels of fitness at time 2 were not more skilled at time 1 than those with lower level fitness at time 1.  Boys with lower motor competence at 6 years old were less aerobically fit at age 10 years.  Boys with the highest level of fitness at age 10 were significantly more skilled at age 6 than those with medium or lower fitness. There was not a difference in skills at age 6 years between those with middle and low fitness levels at age 10. | |
| [33] dos Santos et al., 2018 | Portugal | | N/A | 4 (T1 to T2 = 1 year  T2 to T3 = 1 year  T3 to T4 = 1 year) | 245 (123 M, 122 F) | T1 = 6  T2 = 7  T3 = 8  T4 = 9 | KTK  *Product* | **Total Score**  ***Males***  T1: 111.0 (31.1)  T2: 140.5 (36.6)  T3: 164.6 (37.0)  T4: 182.1 (39.8)  ***Females***  T1: 99.5 (27.2)  T2: 130.9 (30.7)  T3: 157.8 (34.2)  T4: 174 (38.6) | Fitnessgram (health related) and the American Alliance for Health, Physical Education, Recreation, and Dance (performance related)  1-mile run/walk, curl-ups, push-ups, trunk lifts, standing long jump, handgrip strength | **1 Mile Run**  ***Males***  T1: 13.1 (2.7)  T2: 13.8 (4.0)  T3: 13.4 (3.6)  T4: 12.3 (2.7)  ***Females***  T1: 14.2 (2.7)  T2: 14.6 (3.5)  T3: 14.3 (3.1)  T4: 13.7 (2.4)  **Push-Up**  ***Males***  T1: 9.3 (8.8)  T2: 10.9 (8.8)  T3: 16.0 (11.0)  T4: 16.0 (13.3)  ***Females***  T1: 6.8 (7.1)  T2: 9.0 (7.3)  T3: 13.4 (12.2)  T4: 10.2 (8.9)  **Curl-Up**  ***Males***  T1: 12.9 (18.8)  T2: 12.6 (15.3)  T3: 25.7 (33.5)  T4: 26.7 (2.6)  ***Females***  T1: 12.3 (15.2)  T2: 11.5 (14.1)  T3: 20.2 (21.7)  T4: 21.0 (21.7)  **Trunk Lift**  ***Males***  T1: 26.3 (8.3)  T2: 26.8 (6.0)  T3: 29.3 (5.4)  T4: 26.7 (25.6)  ***Females***  T1: 27.2 (6.6)  T2: 28.3 (6.3)  T3: 31.0 (5.6)  T4: 33.8 (7.8)  **Standing Long Jump**  ***Males***  T1: 95.9 (18.5)  T2: 107.0 (22.1)  T3: 111.1 (23.7)  T4: 116.3 (38.4)  ***Females***  T1: 88.6 (15.1)  T2: 102.3 (21.5)  T3: 105.8 (20.0)  T4: 104.7 (38.5)  **Handgrip**  ***Males***  T1: 107.0 (22.1)  T2: 12.1 (2.7)  T3: 13.5 (3.0)  T4: 15.7 (3.3)  ***Females***  T1: 8.2 (1.7)  T2: 10.2 (2.1)  T3: 11.6 (2.7)  T4: 13.4 (2.9) | Multilevel Modeling with Repeated Measures | |  | | **HRF🡪MC**  **Standing Long Jump**  Estimate = 0.02 (SE = 0.007)***  **Shuttle Run**  Estimate = 0.03 (SE = 0.007)**  **Handgrip**  Estimate = 0.06 (SE = 0.01)***  **One Mile Run/Walk**  Estimate = 0.02 (SE = 0.007)**  **Curl-Ups**  Estimate = 0.008 (SE 0.006)  **Push-Ups**  Estimate = 0.004 (SE = 0.007)  **Trunk Lift**  Estimate = 0.008 (0.007)** | | Fitness measures including standing long jump, 50-yard dash, shuttle run, handgrip, one mile walk/run, and trunk lift were associated with change in MC over time. Curl-ups and push-ups were not associated with change in MC over time. | |
| [51] Fransen et al., 2014 | Belgium | | N/A | 2 (2 years) | 501 (268 M, 233 F) | 8.2 (1.2)  T1 Age Cohort 1: 6-7.99  T1 Age Cohort 2: 8.00-9.99 | KTK  *Product* | NR-participants stratified into tertiles based on level of MC | BOT-2 (sit-ups, push-ups)  EUROFIT (sit and reach, handgrip, standing long jump, 20 meter shuttle run | ***T1 only***  **Total**  **Sit and Reach**  21.0 (5.5)  **Sit-Ups**  17.8 (7.3)  **Push-Ups**  21.9 (5.5)  **Handgrip**  16.1 (4.3)  **Standing Long Jump**  119.5 (20.3**)**  **20 Meter Shuttle Run**  4.0 (2.0)  T1 | Repeated-measures Multiple analysis of covariance |  | | **MC 🡪HRF**  **Sit-up**  **MC**  ***Cohort 1***  F = 7.37**  ***Cohort 2***  F = 14.37**  **Time x Sit-Up MC**  ***Cohort 1***  F = 0.09  ***Cohort 2***  F = 1.09  **Push-up**  **MC**  ***Cohort 1***  F = 19.30**  ***Cohort 2***  F = 28.73**  **Time x Push-Up MC**  ***Cohort 1***  F = 0.41  ***Cohort 2***  F = 1.48  **Sit and reach**  **MC**  ***Cohort 1***  F = 4.93**  ***Cohort 2***  F = 3.84*  **Time x Sit-and-Reach MC**  ***Cohort 1***  F = 0.85  ***Cohort 2***  F = 0.32  **Handgrip**  **MC**  ***Cohort 1***  F = 6.08**  ***Cohort 2***  F = 0.48  **Time x Handgrip MC**  ***Cohort 1***  F = 0.13  ***Cohort 2***  F = 1.33  **Standing long jump**  **MC**  ***Cohort 1***  F = 61.45**  ***Cohort 2***  F = 59.20**  **Time x Standing Long Jump MC**  ***Cohort 1***  F = 0.37  ***Cohort 2***  F = 2.28  **20 meter shuttle run**  **MC**  ***Cohort 1***  F = 11.85**  ***Cohort 2***  F = 23.85**  **Time x 20-meter shuttle run MC**  ***Cohort 1***  F = 1.26  ***Cohort 2***  F = 0.11 | | Children with higher motor competence had better physical fitness than children with low motor competence. These differences remained constant over time for all fitness measures. | |  |
| [75] Haugen et al., 2018 | Norway | | N/A | 4 (T1 to T2 = 1 year  T2 to T3 = 5 years  T3 to T4 = 3 years) | 49 (17 M, 32 F) | T1: 5-6 | KTK  *Product* | **Total Scores**  ***Males***  T1: 92.4 (10.5)  T2: 97.1 (11.0)  T3: 96.5 (15.9)  T4: 119.9 (13.6)  ***Females***  T1: 88.5 (12.8)  T2: 96.3 (12.6)  T3: 94.8 (18.9)  T4: 108.9 (19.0) | *T1, T3, and T4 only*  Physical Fitness Test (standing broad jump, two-legged jump, one legged leap, medicine ball push, climbing wall bars, reduced Cooper test, throwing [T1 and T2] replaced with push-up test [T3 and T4]; Fjortoft, Pedersen, Sigmundsson, & Vereijken, 2003) | **Standing Long jump**  ***Males***  T1: 1.3(0.2)  T3: 1.7 (0.2)  T4: 2.1 (0.2)  ***Females***  T1: 1.2(0.2)  T3: 1.7 (0.2)  T4: 1.8 (0.2)  **Two legged jump**  ***Males***  T1: 4.2(0.6)  T3: 3.1 (0.4)  T4: 2.5 (0.3)  ***Females***  T1: 4.1(0.7)  T3: 3.1 (0.5)  T4: 2.8 (2.8)  **One legged leap**  ***Males***  T1: 3.5(0.6) T3: 2.7 (0.3)  T4: 2.2 (0.3)  ***Females***  T1: 3.7(0.6)  T3: 2.6 (0.3)  T4: 2.5 (2.5)  **Throwing ball**  ***Males***  T1: 11.3(3.7)  ***Females***  T1: 6.5(1.7)  **Push-ups**  ***Males***  T3: 26.0(5.7)  T4: 25.8(9.2)  ***Females***  T3: 21.0(3.9)  T4: 31.3 (12.4)  **Medicine Ball Push**  ***Males***  T1: 3.2(0.6)  T3: 6.6 (0.7)  T4: 9.1 (1.0)  ***Females***  T1: 2.5(0.4)  T3: 6.2 (1.0)  T4: 7.2 (1.1)  **Wall Climb**  ***Males***  T1: 12.1(3.6)  T3: 4.3 (0.9)  T4: 3.9 (1.2)  ***Females***  T1: 13.8(2.8)  T3: 4.3 (0.8)  T4: 4.3 (0.8)  **Reduced Cooper Test**  ***Males***  T1: 804 (155.1)  T3: 1012 (108.6)  T4: 1076 (111.2)  ***Females***  T1: 787 (150.4)  T3: 934 (102.2)  T4: 1003 (77.6) | Correlation | |  | | **MC🡪HRF**  **T1 MC and T3 Fitness**  r = 0.60**  **T1 MC and T4 Fitness**  r = 0.52**  **T2 MC and T3 Fitness**  r = 0.55**  **T2 MC and T4 Fitness**  r = 0.46**  **T3 MC and T4 Fitness**  r = 0.70** | | Higher MC at 5-6 years of age was significantly correlated with higher physical fitness 6 and 10 years later compared to children with lower MC. | |
| [40] Henrique et al., 2018 | Portugal | | N/A | 4 (1 year) | 245 (123 M, 122 F) | T1: 6.46– 9.46 | KTK  *Product* | **Walking backwards**  ***Males***  T1: 29.0 (13.8)  T2: 37.9 (13.9)  T3: 43.3 (13.7)  T4: 48.3 (13.0)  ***Females***  T1: 28.6 (15.5)  T2: 36.6 (14.7)  T3: 40.5 (11.5)  T4: 46.6 (13.7)  **Jumping sideways**  ***Males***  T1: 32.0 (9.8)  T2: 37.7 (11.0)  T3: 44.6 (13.9)  T4: 54.7 (13.2)  ***Females***  T1: 28.6 (8.9)  T2: 36.0 (9.8)  T3: 43.9 (13.6)  T4: 52.5 (12.0)  **Hopping for height**  ***Males***  T1: 18.6 (11.8)  T2: 27.5 (16.0)  T3: 38.0 (17.9)  T4: 43.5 (18.5)  ***Females***  T1: 15.3 (10.1)  T2: 23.0 (13.6)  T3: 35.3 (17.4)  T4: 38.4 (17.8)  **Moving sideways**  ***Males***  T1: 30.5 (5.0)  T2: 36.9 (6.5)  T3: 40.3 (5.5)  T4: 41.9 (7.6)  ***Females***  T1: 27.6 (5.2)  T2: 35.7 (6.5)  T3: 39.0 (7.2)  T4: 40.4 (6.9)  **Motor quotient**  ***Males***  T1: 94.9 (13.9)  T2: 98.8 (16.1)  T3: 97.0 (16.3)  T4: 88.0 (17.4)  ***Females***  T1: 81.7 (14.3) T2: 89.5 (15.0)  T3: 92.9 (16.6)  T4: 79.2 (16.9) | Fitnessgram test battery and AAHPER Youth Fitness Test (1 mile run/walk, push-ups, curl-ups, trunk lift, standing long jump, handgrip) | **1-mile run/walk**  ***Upper Canal***  T1: 12.3(2.3)  ***Lower Canal***  T1: 14.9(3.0)  **Push-ups**  ***Upper Canal***  T1: 9.1(10.8)  ***Lower Canal***  T1: 7.6 (7.1)  **Curl-Ups**  ***Upper Canal***  T1: 29.0(22.7)  ***Lower Canal***  T1: 5.5(12.5)  **Trunk Lifts**  ***Upper Canal***  T1: 31.0(8.3)  ***Lower Canal***  T1: 25.3(6.3)  **Standing Long Jump**  ***Upper Canal***  T1: 104.8 (19.0)  ***Lower Canal***  T1: 81.8(12.5)  **Handgrip Strength**  ***Upper Canal***  T1: 10.3(2.1)  ***Lower Canal***  T1: 8.8 (2.1) | T-test | |  | | **HRF🡪MC (T1)**  **1-mile run/walk**  t = 4.25**  **Push-ups**  t = -0.77*  **Curl-Ups**  t = -5.98**  **Trunk Lifts**  t = -3.53**  **Standing Long Jump**  t = -6.57**  **Handgrip Strength**  t = -3.03** | | Individuals who persistently stayed in the upper canal (i.e., high MC) across four years had higher levels of fitness age 6 years of age.  Individuals who were consistently in the lower canal (i.e., low MC) had worse fitness performances at 6-years of age. | |
| [36] Jaakkola, Yli-Piipari, Huhtiniemi, et al., 2019 | Finland | | N/A | 2 (1 year) | 491 (216 M, 275 F) | *T1:* 11.3^2^ (0.3)  *T2:* 12.3 (0.3) | 5‐leaps test (locomotor), throwing‐catching combination test (manipulative), two‐legged jumping from side to side test (balance/stability)  *Product* | **5-leaps test**  T1: 7.8(0.9)  T2: 8.37(1.1)  **Throwing-catching**  T1: 12.6(4.8)  T2: 14.3(4.3)  **Two-legged jumping**  T1: 36.6(6.5)  T2: 40.4(7.1) | 20 meter shuttle run, curl-ups, push-ups | **20 Meter Shuttle Run**  ***Males***  T2 = 42.94 (22.91)  ***Females***  T2 = 38.69 (18.02)  **Curl-Ups**  ***Males***  T2 = 40.69 (22.28)  ***Females***  T2 = 39.76 (21.40)  **Push-ups**  ***Males***  T2 = 18.10 (13.69)  ***Females***  T2 = 24.31 (11.94) | Cross-lagged Structural Equation Model panel analysis | |  | | **MC (T1) 🡪HRF (T2)**  **Balance/Stability and Muscular Fitness**  ***Males***  Pathway removed and beta not reported  ***Females***  β = 0.14*  **Balance/Stability and Cardiorespiratory Endurance**  ***Males***  Pathway removed and beta not reported  ***Females***  Pathway removed and beta not reported  **Object Control and Muscular Fitness**  ***Males***  Pathway removed and beta not reported  ***Females***  Pathway removed and beta not reported  **Object Control and Cardiorespiratory Endurance**  ***Males***  Pathway removed and beta not reported  ***Females***  Pathway removed and beta not reported  **Locomotor and Muscular Fitness**  ***Males***  β = 0.19*  ***Females***  β = 0.11*  **Locomotor and Cardiorespiratory Endurance**  ***Males***  Pathway removed and beta not reported  ***Females***  Pathway removed and beta not reported  **HRF (T1) 🡪MC (T2)**  **Muscular Fitness and Balance/Stability**  ***Males***  Pathway removed and beta not reported  ***Females***  β = 0.16*  **Cardiorespiratory Endurance and Balance/Stability**  ***Males***  β = 0.18*  ***Females***  β = 0.11*  **Muscular Fitness and Object Control**  ***Males***  Pathway removed and beta not reported  ***Females***  Pathway removed and beta not reported  **Cardiorespiratory Endurance and Object Control**  ***Males***  Pathway removed and beta not reported  ***Females***  Pathway removed and beta not reported  **Muscular Fitness and Locomotor**  ***Males***  Pathway removed and beta not reported  ***Females***  Pathway removed and beta not reported  **Cardiorespiratory Endurance and Locomotor**  ***Males***  β = 0.23*  ***Females***  β = 0.12* | | Locomotor skills measured at Grade 5 predicted muscular fitness (but not cardiovascular endurance) at Grade 6 in both sex groups.  Object control skills in Grade 5 were not a predictor of cardio respiratory endurance or muscular fitness Grade 6 for boys or girls.  Balance/Stability skills at Grade 5 predicted muscular endurance at Grade 6 for girls (but not boys). Balance/Stability skills at Grade 5 did not predict cardiorespiratory endurance for either sex.  Cardiorespiratory endurance in Grade 5 associated with locomotor and Balance/Stability skills at Grade 6 in both boys and girls.  Muscular fitness at Grade 5 not associated with locomotor skills at Grade 6 for either sex.  Muscular fitness at Grade 5 was associated with Balance/Stability skills at Grade 6 for girls but not boys.  Cardiorespiratory endurance or muscular fitness in Grade 5 did not predict future object control competence in Grade 6. | |
| [62] Lima, Bugge, Ersbøll, Stodden, and Andersen (2019) | Denmark | | N/A | 3 (T1 to T2 = 3 years  T2 to T3 = 4 years) | *T1:*696 (369 M, 327 F)  *T2:* 615 (323 M, 292 F)  *T3:* 442 (231 M, 211 F) | *T1:* 6.75 (0.4)  *T2:* 9.59 (1.1)  *T3:* 13.4 (0.3) | KTK  *Product* | **Total Scores**  ***Total***  T1: 119.2 (27.7)  T2: 195.2 (34.6)  T3: 249.4 (29.4)  ***Males***  T1: 120.1 (28.4)  T2: 194.8 (34.9)  T3: 251.4 (29.9)  ***Females***  T1: 118.2 (26.8)  T2: 195.6 (34.4)  T3: 247.3 (28.8) | VO_2peak_ (continuous running on a treadmill)  T1 and T2: AMIS 2001 Cardiopulmonary Function Test System  T3: COSMED K4b^2^ | **VO_2peak_**  ***Total***  T1: 46.7 (6.0)  T2: 49.1 (7.1)  T3: 49.3 (8.7)  ***Males***  T1: 48.5 (5.9)  T2: 51.8 (6.8)  T3: 53.2 (8.4)  ***Females***  T1: 44.8 (5.4)  T2: 45.9 (6.2)  T3: 45.2 (7.0) | Multilevel Linear Regressions | |  | | **MC 🡪HRF**  ***Males***  0.34 Z-scores, 95% CI: 0.27-0.40.  ***Females***  0.27 Z-scores, 95% CI: 0.20-0.33.  **HRF🡪MC**  ***Males***  0.24 Z-scores, 95% CI: 0.18-0.30).  ***Females***  0.25 Z-scores, 95% CI: 0.18-0.32) | | For boys, MC influenced the development of VO_2peak_ more than VO_2peak_ influenced the development of MC.  For girls, there was not a marked difference in MC influencing the development of VO_2peak_ or vice versa  Overall, in boys, independent of the direction analysed, there was an increase in the strength of the association from 6 to 9 years-of-age and subsequent maintenance at 13 years-of-age.  Overall in girls, the strength of the association that MC had on the development of VO2peak was relatively stable during the follow-up period (0.24 ≤ ˇ ≥ 0.30). | |
|  | | **Intervention Studies** | | | | | | | | | | | | | | | | |
| [59] Cohen et al., 2015 | Australia | | *Dose:* 12 month intervention; detailed overview not in current article  *Framework/Theory:* Socioecological Framework  *Approach:*  (1) Teacher professional learning  (2) Student leadership  (3) School committee and physical activity policies  (4) Provision of physical activity equipment  (5) Parental engagement (newsletters, parent evening and FMS homework)  (6) School-community sport and physical activity links | 2 (1 year) | 460 (212 M, 248 F) | 8.5 (0.6) | TGMD-2  *Process* | NR; Cohen et al. 2014 | 20 Meter Shuttle Run | NR; Cohen et al. 2014 | Multilevel linear regressions | |  | | **Intervention🡪MC🡪Cardiorespiratory Endurance**  **Locomotor**  B = 0.74*  **Object Control**  B = 0.45  **Total Skill**  B = 1.19* | | The effect of the intervention on cardiorespiratory endurance was mediated by both locomotor skills and total skills. Object control skills did not mediate the effects of the intervention on cardiorespiratory fitness. | |
| * Reported within article, p < 0.05  ** Reported within article, p < 0.01  *** Reported within article, p <0.001  ^1^ = Significance levels of results not reported  ^2^ = Mean age is unclear as it is reported differently in three sections of manuscript  Note.  F = Females  HRF= Health-related fitness  KTK = Körperkoordinationstest Für Kinder  M = Male  MC = Motor competence  N/A = Not Applicable  SD = Standard deviation  TGMD = Test of Gross Motor Development | | | | | | | | | | | | | | | | | | |
