# Supplementary material for: Through the Looking Glass: A Systematic Review of Longitudinal Evidence, Providing New Insight for Motor Competence and Health
Source: Sports Med. 2021 Aug 31;52(4):875–920. doi: 10.1007/s40279-021-01516-8 (PMC8938405; doi:10.1007/s40279-021-01516-8)
Supplement: Supplementary file 7 — Supplementary file7 (DOCX 33 kb) [file 40279_2021_1516_MOESM7_ESM.docx]

| **Supplementary Table 7. Health-Related Fitness Mediating the Motor Competence and Physical Activity Relationship Results** | | | | | | | | | | | | | |
| --- | --- | --- | --- | --- | --- | --- | --- | --- | --- | --- | --- | --- | --- |
| ***Mediation Studies*** | | | | | | | | | | | | | |
| **Study** | **Country** | **Timepoints # (Duration)** | **Sample #**  ***(M, F)*** | **Age (SD)** | **MC measure** | **MC scores at each timepoint**  ***Mean (SD)*** | **Fitness measure** | **Fitness scores at each timepoint**  ***M (SD)*** | **PA measure**  ***Objective/Subjective***  **Duration of measurement** | **PA scores at each timepoint**  ***M (SD)*** | **Analysis** | **Pathway tested and values** | **Overall findings** |
| [34] Britton et al., 2019 | Ireland | 2 (1 year) | 224 (110 M, 114 F) | 12.3 (0.0) | TGMD-3 (kick, catch, overhand throw, one- and two-hand strike, run, skip, horizonal jump) *Process*  Victoria Department of Education Training Manual (vertical jump)  *Process*  MABC-2 (two-board balance, zigzag hop, backward walking toe to heel)  *Product* | **Locomotor skills**  ***Males***  *T1:* 29.6 (3.5)  *T2:* 31.1 (2.8)  ***Females*** *T1:* 29.2 (3.6)  *T2:* 29.5 (3.3)  **Object control skills**  ***Males***  *T1:* 33.2 (4.2)  *T2:* 36.4 (2.3)  ***Females*** *T1:* 26.1 (7.0)  *T2:* 30.5 (4.6)  **Balance/Stability**  ***Males***  *T1:* 36.6 (8.1)  *T2:* 38.6 (6.7)  ***Females***  *T1:* 36.4 (8.7)  *T2:* 39.2 (6.9) | 20 meter shuttle run, horizontal and vertical jump, push-ups, curl ups  (methods derived from Fitnessgram, Eurofit test battery, and the HELENA study; Ortega, Artero, et al., 2008) | **Composite Score**  ***Males***  T1: 1.6 (3.1)  T2: 1.8 (3.5)    ***Females***  T1: -1.4 (2.4)  T2: -1.6 (2.7) | Accelerometer (ActiGraph GT1M, GT3X, GT3X+, wGT3X-BT); MVPA mins  *Objective,* 7 days during waking hours | **MVPA Minutes**  ***Males***  *T1:* 54.4 (26.3)  *T2:* 44.2 (19.2)    ***Females*** *T1:* 46.3 (16.0)  *T2:* 37.1 (11.7) | SEM | **PA🡪HRF🡪MC**  **PA(T1)🡪HRF (T1)**  β = 0.36***  **HRF (T1)🡪MC (T2)**  **Object Control**  β = 0.38***  **Locomotor**  β = 0.37***  **Balance/Stability**  β = 0.38***  **MC🡪HRF🡪PA**  **MC(T1)🡪HRF (T1)**  **Object Control**  β = 0.30***  **Locomotor**  β = 0.26***  **Balance/Stability**  β = 0.25***  **HRF(T1)🡪PA (T2)**  β = 0.43*** | Reciprocal relationships between MC and PA were mediated by HRF. HRF was a mediator regardless of the MC subscale explored. Pathways were stronger from PA to MC, as opposed to MC to PA. |
| [72] Burns et al., 2018 | USA | 1 | 84 (44 M, 40 F) | 11.6 (0.6) | TGMD-3  *Process* | **Locomotor Skills**  ***Males***  39.0 (6.3)  ***Females***  36.1 (5.8)  ***Total***  37.9 (6.1)  **Ball Skills**  ***Males***  48.9 (6.2)  ***Females***  39.4 (5.2)  ***Total***  41.2 (7.2)  **Total Scores**  ***Males***  88.1 (10.9)  ***Females***  74.9 (7.8)    ***Total***  80.5 (10.9) | 20 meter shuttle run | **Cardiorespiratory Fitness**  ***Males***  35.3 (20.9)  ***Females***  22.0 (19.4)  ***Total***  26.4 (19.3) | Pedometer  (Yamax Digi Walker CW600); steps  *Objective,* 5 school days (8am-3pm) | **School Day Steps**  ***Males***  4214 (1435)  ***Females***  3376 (1579)  ***Total***  3681 (1577) | SEM | **PA🡪Cardiorespiratory endurance🡪MC (total scores)**  **PA🡪 Cardiorespiratory endurance**  B = 0.07*  β = 0.50*  **Cardiorespiratory endurance🡪 MC**  B = 0.14*  β = 0.28*  **PA🡪MC**  B = 0.01  β = 0.13 | The association between PA and overall MC is fully mediated by cardiorespiratory endurance. |
| [74] Jaakkola, Huhtiniemi, Salin, et al., 2019 | Finland | 1 | 422 (176 M, 246 F) | 11.3 (0.3) | Leaping test, throwing-catching combination test, two-legged jumping from side to side  *Product* | **MC sum scores^a^**  ***Males***  0.15 (0.82)  ***Females***  0.06 (0.75)  **5‐leaps test**  ***Males***  7.8 (0.9)  ***Females***  7.8 (0.9)  **Throwing‐catching combination test**  ***Males***  12.5 (4.9)  ***Females***  9.9 (5.0)  **Two‐legged jumping from side to side test**  ***Males***  74.3 (12.9)  ***Females***  77.6 (12.5) | 20 m shuttle run , abdominal muscle en- durance test, push‐up tests | **Sum Z-Scores**  ***Males***  0.07 (1.02)  ***Females***  0.20 (0.96)  **20 m Shuttle Run**  ***Males***  41.5 (20.5)  ***Females***  34.0 (15.3)  **Abdominal Muscles Endurance**  ***Males***  38.8 (21.4)  ***Females***  41.6 (23.6)  **Push-Ups**  ***Males***  18.5 (11.6)  ***Females***  27.6 (11.7) | Accelerometer (ActiGraph GT3X+); MVPA  *Objective,* 7 days during waking hours | **MVPA Minutes**  ***Males***  64.2 (24.7)  ***Females***  55.3 (21.0) | SEM | **MC🡪 HRF🡪 PA**  **MC🡪 HRF**  β = 0.68*  **HRF🡪PA**  β = 0.26*  **MC🡪 PA (boys only)**  β = 0.29*  **MC🡪 HRF🡪 PA (girls)**  β = 0.19***  **MC🡪 HRF🡪 PA (boys)**  β = 0.20***  **PA🡪 HRF🡪 MC**  **PA🡪 HRF**  β = 0.43*  **HRF🡪 MC**  β = 0.56*  **PA🡪 MC (boys only)**  β = 0.15*  **PA🡪 HRF🡪 MC (girls)**  β = 0.21***  **PA🡪 HRF🡪 MC (boys)**  β = 0.25*** | HRF (a standardized physical  fitness sum variable) mediated the relationship from MC to MVPA and from MVPA to MC for both boys and girls.  For boys, results showed that MC and MVPA were directly and reciprocally related to one another. Despite the presence of a mediated (and reciprocal) path in both boys and girls, there was a full mediation for girls (for whom, the direct path MC to PA and PA to MC was no longer significant), but partial mediation for boys (for whom, the direct path MC to PA and PA to MC remained significant after accounting for the mediated path. |
| [69] Khodaverdi et al., 2015 | Iran | 1 | 352 (352 F) | 8.7 (0.3) | TGMD-2  *Process* | **Total Scores**  76.3 (9.3)  **Locomotor skills**  41.9 (6.6)  **Object control skills**  34.3 (5.5) | Fitnessgram  (90◦ push-up, curl-up, back-saver sit and reach)  600-yard running/walking test (McSwegin et al., 1989) | **Push-Up** 12.7 (7.6)  **Curl-Up** 27.1 (16.8)  **Back-saver sit and reach** 31.0 (4.9)  **600-yard running/ walking** 226.6 (39.5) | Physical Activity Questionnaire for Older Children (Faghihimani et al., 2010); scale of 1-5 (1 indicates low PA)  *Subjective,* 7 day recall of MVPA | 3.3 (0.9) | Preacher and Hayes Bootstrapping method | **MC🡪HRF🡪PA**  **(only locomotor, run/walk)**  **Locomotor MC🡪 HRF**  b = -1.37**  **HRF🡪 PA**  b = -0.34***  **LocomotorMC🡪 PA**  b = 0.32***  **Indirect effect**  b = 0.28 (significant, but level unknown)  Object control skills not explored as mediators, as significant correlation with fitness not found | Results demonstrate that fitness (using 600-yard test) partially mediates the relationship between locomotor skills and physical activity.  There were no significant mediation results between object control skills and PA. Fitness (600- yard test) was the only HRF component included as a mediator in the full model. |
| [37] Lima et al. (2017) | Denmark | 3 (T1 to T2 = 3 years,  T2 to T3 = 4 years) | *T1 =* 696  *T2 =* 617  *T3 =* 513 | *T1:* 6.8 (0.4)  *T2:* 9.6 (1.1)  *T3:* 13.4 (0.3) | KTK  *Product* | **KTK Total Score**  ***Total***  T1: 119.2 (27.7)  T2: 195.2 (34.6)  T3: 249.4 (29.4)  ***Males***  T1: 120.1 (28.4)  T2: 194.8 (34.9)  T3: 251.4 (29.9)  ***Females***  T1: 118.2 (26.8)  T2: 195.6 (34.4)  T3: 247.3 (28.8) | VO_2peak_ (continuous running on a treadmill)  T1 and T2: AMIS 2001 Cardiopulmonary Function Test System  T3: COSMED K4b^2^ | **VO_2peak_**  ***Total***  T1: 46.7 (6.0)  T2: 49.1 (7.1)  T3: 49.3 (8.7)  ***Males***  T1: 48.5 (5.9)  T2: 51.8 (6.8)  T3: 53.2 (8.4)  ***Females***  T1: 44.8 (5.4)  T2: 45.9 (6.2)  T3: 45.2 (7.0) | Accelerometer (ActiGraph 7164 at T1 and ActiGraph GT1M at T2, T3)  *Objective,* 4 days of MVPA and VPA reported | **MVPA**  ***Total***  T1: 76.5 (27.0)  T2: 73.0 (25.5)  T3: 53.2 (24.6)  ***Males***  T1: 82.7 (29.6)  T2: 78.1 (25.8)  T3: 59.9 (26.5)  ***Females***  T1: 69.7 (21.8)  T2: 67.5 (24.1)  T3: 6.0 (20.1)  **VPA**  ***Total***  T1: 28.3 (14.0)  T2: 29.9 (15.1)  T3: 20.8 (13.3)  ***Males***  T1: 31.1 (15.6)  T2: 32.6 (15.8)  T3: 23.3 (14.1)  ***Females*** T1: 25.2 (11.2)  T2: 27.1 (13.8)  T3: 8.0 (11.8) | SEM | **MVPA🡪HRF🡪MC**  β = 0.07*  **VPA🡪HRF🡪MC**  β = 0.09*  **MC🡪HRF🡪MVPA**  β = 0.05*  **MC🡪HRF🡪VPA**  β = 0.06* | Both MVPA and VPA were indirectly associated with MC via fitness mediation.  MC was indirectly associated with both MVPA and VPA through fitness mediation |
| * Reported within article, p < 0.05  ** Reported within article, p < 0.01  *** Reported within article, p <0.001  Note.  F = Females  HRF = Health-related fitness  M = Male  MABC = Movement Assessment Battery for Children  MC = Motor competence  MVPA = Moderate-to-vigorous physical activity  PA = Physical activity  SD = Standard deviation  TGMD = Test of Gross Motor Development | | | | | | | | | | | | | |
