# Supplementary material for: Through the Looking Glass: A Systematic Review of Longitudinal Evidence, Providing New Insight for Motor Competence and Health
Source: Sports Med. 2021 Aug 31;52(4):875–920. doi: 10.1007/s40279-021-01516-8 (PMC8938405; doi:10.1007/s40279-021-01516-8)
Supplement: Supplementary file 8 — Supplementary file8 (DOCX 32 kb) [file 40279_2021_1516_MOESM8_ESM.docx]

Table 8: Risk of bias assessment according to the National Institute of Health and listed according to study design

|  | ***Intervention studies*** |  | | | | | | | | | | | | | |  |
| --- | --- | --- | --- | --- | --- | --- | --- | --- | --- | --- | --- | --- | --- | --- | --- | --- |
| *Reference*  *Number* | *Study* | *Item 1* | *Item 2* | *Item 3* | *Item 4* | *Item 5* | *Item 6* | *Item 7* | *Item 8* | *Item 9* | *Item 10* | *Item 11* | *Item 12* | *Item 13* | *Item 14* | n of Yes |
|  | n(%) of studies with a Yes | 4 (100%) | 3  (75%) | 2  (50%) | 0 (0%) | 0  (0%) | 2  (50%) | 3 (75%) | 3 (75%) | 1 (25%) | 4 (100%) | 3 (75%) | 2 (50%) | 3 (75%) | 4 (100%) |  |
| [59] | Cohen, Morgan, Plotnikoff, Barnett, and Lubans (2015) | Yes | Yes | Yes | No | No | CD | No | CD | Yes | Yes | Yes | Yes | Yes | Yes | 9 |
| [66] | Lander, Mergen, Morgan, Salmon, and Barnett (2019) | Yes | No | No | CD | CD | Yes | Yes | Yes | CD | Yes | Yes | CD | Yes | Yes | 8 |
| [67] | Marouli, Papavasileiou, Dania, and Venetsanou (2016) | Yes | Yes | CD | No | No | No | Yes | Yes | CD | Yes | CD | No | No | Yes | 6 |
| [60] | McGrane, Belton, Fairclough, Powell, and Issartel (2018) | Yes | Yes | Yes | No | No | Yes | Yes | Yes | CD | Yes | Yes | Yes | Yes | Yes | 11 |
|  | ***Mediation studies*** |  | | | | | | | | | | | | | |  |
|  | *Study* | *Item*  *1* | *Item 2* | *Item 3* | *Item 4* | *Item 5* | *Item 6* | *Item 7* | *Item 8* | *Item 9* | *Item 10* | *Item 11* | *Item 12* | *Item 13* | *Item 14* |  |
|  | n(%) of studies with a Yes | 10  (100%) | 9  (90%) | 3  (30%) | 9  (90%) | 9  (90%) | 3  (30%) | 3  (30%) | 8  (80%) | 9  (90%) | 2  (20%) | 10  (100%) | 1  (10%) | 2  (20%) | 6  (60%) |  |
| [34] | Britton, Belton, and Issartel (2019) * | Yes | No | CD | CD | Yes | Yes | Yes | Yes | Yes | Yes | Yes | CD | Yes | Yes | 10 |
| [72] | Burns and Fu (2018) | Yes | Yes | CD | Yes | Yes | No | No | Yes | Yes | No | Yes | CD | CD | Yes | 8 |
| [68] | Chan, Ha, Ng, and Lubans (2019) | Yes | Yes | Yes | Yes | Yes | No | No | Yes | Yes | No | Yes | Yes | CD | Yes | 10 |
| [73] | Crane, Naylor, Cook, and Temple (2015) | Yes | Yes | No | Yes | Yes | No | No | Yes | Yes | No | Yes | CD | CD | Yes | 8 |
| [70] | Fu and Burns (2018) | Yes | Yes | CD | Yes | Yes | No | No | Yes | Yes | No | Yes | CD | CD | Yes | 8 |
| [71] | Gu, Thomas, and Chen (2017) | Yes | Yes | CD | Yes | Yes | No | No | No | Yes | No | Yes | CD | CD | No | 6 |
| [74] | Jaakkola, Huhtiniemi, et al. (2019) | Yes | Yes | CD | Yes | No | No | No | Yes | Yes | No | Yes | CD | CD | No | 6 |
| [38] | Jekauc, Wagner, Herrmann, Hegazy, and Woll (2017) * | Yes | Yes | Yes | Yes | Yes | Yes | Yes | CD | CD | No | Yes | CD | No | No | 8 |
| [69] | Khodaverdi, Bahram, Stodden, and Kazemnejad (2016) | Yes | Yes | No | Yes | Yes | No | No | Yes | Yes | No | Yes | CD | Yes | No | 8 |
| [37] | Lima, Pfeiffer, Larsen, et al. (2017) | Yes | Yes | Yes | Yes | Yes | Yes | Yes | Yes | Yes | Yes | Yes | CD | No | Yes | 12 |
|  | ***Longitudinal studies*** |  | | | | | | | | | | | | | |  |
|  | *Study* | *Item*  *1* | *Item 2* | *Item 3* | *Item 4* | *Item 5* | *Item 6* | *Item 7* | *Item 8* | *Item 9* | *Item 10* | *Item 11* | *Item 12* | *Item 13* | *Item 14* |  |
|  | n(%) of studies with a Yes | 32 (100%) | 26  (81%) | 13  (41%) | 28 (89%) | 16  (50%) | 30  (94%) | 30 (94%) | 29 (91%) | 29 (91%) | 26  (81%) | 29 (91%) | 6 (19%) | 13  (41%) | 27 (84%) |  |
| [42] | Antunes et al. (2016) | Yes | Yes | No | Yes | Yes | Yes | Yes | Yes | Yes | Yes | Yes | CD | Yes | Yes | 12 |
| [39] | Barnett et al. (2016) | Yes | Yes | CD | Yes | Yes | Yes | Yes | Yes | Yes | Yes | Yes | CD | No | Yes | 11 |
| [34] | Britton et al. (2019) * | Yes | No | CD | CD | Yes | Yes | Yes | Yes | Yes | Yes | Yes | CD | Yes | Yes | 10 |
| [48] | Bryant, James, Birch, and Duncan (2014) | Yes | Yes | CD | Yes | No | CD | CD | Yes | Yes | Yes | Yes | CD | Yes | Yes | 9 |
| [61] | Cheng et al. (2016) | Yes | Yes | No | Yes | Yes | Yes | Yes | Yes | Yes | Yes | Yes | CD | No | Yes | 11 |
| [64] | Coppens et al. (2019) | Yes | Yes | Yes | Yes | Yes | Yes | Yes | Yes | No | Yes | No | CD | Yes | Yes | 11 |
| [52] | De Souza et al. (2014) | Yes | Yes | CD | Yes | No | Yes | Yes | Yes | Yes | Yes | Yes | CD | Yes | Yes | 11 |
| [33] | Dos Santos et al. (2018) | Yes | Yes | CD | Yes | No | Yes | Yes | Yes | Yes | Yes | Yes | CD | No | Yes | 10 |
| [51] | Fransen et al. (2014) | Yes | Yes | CD | CD | No | Yes | Yes | Yes | Yes | Yes | Yes | CD | No | Yes | 9 |
| [56] | Gu (2016) | Yes | Yes | CD | Yes | Yes | No | No | Yes | Yes | No | Yes | CD | Yes | No | 8 |
| [55] | Gu, Keller, Weiller-Abels, and Zhang (2018) | Yes | Yes | CD | Yes | Yes | Yes | Yes | Yes | Yes | CD | Yes | CD | Yes | Yes | 11 |
| [75] | Haugen and Johansen (2018) | Yes | No | No | Yes | Yes | Yes | Yes | Yes | Yes | Yes | Yes | No | CD | No | 9 |
| [58] | Henrique et al. (2016) | Yes | CD | Yes | Yes | CD | Yes | Yes | Yes | Yes | Yes | Yes | CD | No | Yes | 10 |
| [40] | Henrique et al. (2018) | Yes | CD | CD | Yes | CD | Yes | Yes | Yes | Yes | Yes | Yes | CD | CD | Yes | 9 |
| [41] | Herrmann, Heim, and Seelig (2017) | Yes | Yes | Yes | Yes | CD | Yes | Yes | CD | Yes | Yes | Yes | CD | Yes | Yes | 11 |
| [35] | Jaakkola, Hakonen, et al. (2019) | Yes | Yes | Yes | Yes | CD | Yes | Yes | Yes | Yes | Yes | Yes | CD | CD | No | 10 |
| [36] | Jaakkola, Yli-Piipari, et al. (2019) | Yes | Yes | CD | Yes | Yes | Yes | Yes | Yes | Yes | Yes | Yes | CD | Yes | Yes | 12 |
| [47] | Jaakkola, Yli‐Piipari, Huotari, Watt, and Liukkonen (2016) | Yes | CD | CD | No | CD | Yes | Yes | Yes | Yes | Yes | CD | Yes | CD | Yes | 8 |
| [38] | Jekauc et al. (2017) * | Yes | Yes | Yes | Yes | Yes | Yes | Yes | CD | CD | No | Yes | CD | No | No | 8 |
| [54] | Larsen, Kristensen, Junge, Rexen, and Wedderkopp (2015) | Yes | Yes | Yes | Yes | Yes | Yes | Yes | Yes | Yes | Yes | Yes | Yes | No | Yes | 13 |
| [63] | Lima, Bugge, Pfeiffer, and Andersen (2017) | Yes | Yes | Yes | Yes | CD | Yes | Yes | Yes | Yes | Yes | Yes | Yes | No | Yes | 12 |
| [65] | Lima, Pfeiffer, Bugge, et al. (2017) | Yes | Yes | Yes | Yes | CD | Yes | Yes | Yes | Yes | Yes | Yes | CD | No | Yes | 11 |
| [37] | Lima, Pfeiffer, Larsen, et al. (2017) | Yes | Yes | Yes | Yes | Yes | Yes | Yes | Yes | Yes | Yes | Yes | CD | No | Yes | 12 |
| [62] | Lima, Bugge, Ersbøll, Stodden, and Andersen (2019) | Yes | Yes | Yes | Yes | No | Yes | Yes | Yes | Yes | Yes | Yes | CD | No | Yes | 11 |
| [44] | Lloyd, Saunders, Bremer, and Tremblay (2014) | Yes | Yes | CD | Yes | No | Yes | Yes | CD | Yes | Yes | Yes | CD | No | Yes | 9 |
| [49] | Lopes et al. (2019) | Yes | CD | No | CD | No | Yes | Yes | Yes | Yes | Yes | Yes | CD | No | Yes | 8 |
| [45] | McIntyre, Parker, Chivers, and Hands (2018) | Yes | Yes | No | Yes | Yes | Yes | Yes | Yes | Yes | Yes | Yes | No | Yes | Yes | 12 |
| [43] | Reyes et al. (2019) | Yes | Yes | Yes | Yes | CD | Yes | Yes | Yes | Yes | Yes | Yes | CD | Yes | Yes | 12 |
| [57] | Schmutz et al. (2018) | Yes | Yes | CD | Yes | Yes | Yes | Yes | Yes | Yes | Yes | Yes | CD | Yes | Yes | 12 |
| [50] | Smith, Fisher, and Hamer (2015) | Yes | Yes | Yes | Yes | Yes | Yes | Yes | Yes | CD | CD | CD | Yes | Yes | Yes | 11 |
| [46] | Venetsanou and Kambas (2017) | Yes | Yes | CD | Yes | CD | Yes | Yes | Yes | Yes | CD | Yes | Yes | No | No | 9 |
| [53] | Wagner, Jekauc, Worth, and Woll (2016) | Yes | Yes | Yes | Yes | Yes | Yes | Yes | Yes | Yes | CD | Yes | Yes | CD | Yes | 12 |

Notes. Yes = low risk of bias, No = high risk of bias, CD = could not be determined, *Cross-Listed as both Mediation and Longitudinal

***Controlled intervention studies***: 1. Was study described as randomized, a randomized trial, a randomized clinical trial, or an RCT? 2. Was method of randomization adequate (i.e., randomly generated assignment)? 3. Was treatment allocation concealed? 4. Were study participants and providers blinded to treatment group assignment? 5. Were the people assessing the outcomes blinded to the participants' group assignments? 6. Were the groups similar at baseline on important characteristics that could affect outcomes? 7. Was the overall drop-out rate from the study at endpoint 20% or lower of the number allocated to treatment? 8. Was the differential drop-out rate (between treatment groups) at endpoint 15 percentage points or lower? 9. Was there high adherence to the intervention protocols for each treatment group? 10. Were other interventions avoided or similar in the groups? 11. Were outcomes assessed using valid and reliable measures, implemented consistently across all study participants? 12. Did authors report that the sample size was sufficiently large to be able to detect a difference in the main outcome between groups with at least 80% power? 13. Were outcomes reported or subgroups analysed pre-specified (i.e., identified before analyses were conducted)? 14. Were all randomized participants analysed in the group to which they were originally assigned, i.e., did they use an intention-to-treat analysis?

***Observational cohort, longitudinal and mediation studies***: 1. Was the research question or objective clearly stated? 2. Was the study population clearly specified and defined? 3. Was the participation rate of eligible persons at least 50%? 4. Were all the subjects selected or recruited from the same or similar populations (including the same time period)? Were inclusion and exclusion criteria for being in the study pre-specified and applied uniformly to all participants? 5. Was a sample size justification, power description, or variance and effect estimates provided? 6. For the analyses, were the exposure(s) of interest measured prior to the outcome(s) being measured? 7. Was the timeframe sufficient so that one could reasonably expect to see an association between exposure and outcome if it existed? 8. For exposures that can vary in amount or level, did the study examine different levels of the exposure as related to the outcome (e.g., categories of exposure, or exposure measured as continuous variable)? 9. Were the exposure measures (independent variables) clearly defined, valid, reliable, and implemented consistently across all study participants? 10. Was the exposure(s) assessed more than once over time? 11. Were the outcome measures (dependent variables) clearly defined, valid, reliable, and implemented consistently across all study participants? 12. Were the outcome assessors blinded to the exposure status of participants? 13. Was loss to follow-up after baseline 20% or less? 14. Were key potential confounding variables measured and adjusted statistically for their impact on the relationship between exposure(s) and outcome(s)?
